# Phylogenetic analysis and genetic evolution of porcine respiratory coronavirus in Guangxi province, Southern China from 2022 to 2024

**DOI:** 10.3389/fmicb.2025.1625343

**Published:** 2025-07-10

**Authors:** Yuwen Shi, Kaichuang Shi, Yanwen Yin, Shuping Feng, Feng Long, Wenjun Lu, Sujie Qu, Yan Ma, Jiakang He

**Affiliations:** ^1^Guangxi Key Laboratory of Animal Breeding, Disease Control and Prevention, College of Animal Science and Technology, Guangxi University, Nanning, China; ^2^Nanning Kedi Biotechnology Co., Ltd, Nanning, China; ^3^Guangxi Center for Animal Disease Control and Prevention, Nanning, China

**Keywords:** porcine respiratory coronavirus, phylogenetic analysis, genetic evolution, recombination, gene sequence

## Abstract

Porcine respiratory coronavirus (PRCV) is an important coronavirus to cause respiratory syndrome in pigs. To analyze the genetic and evolutional characteristics of PRCV in Guangxi province, southern China, a total of 6,267 clinical samples were collected from different pig farms, harmless treatment plants and abattoirs in Guangxi province during 2022–2024. Seventeen positive samples of PRCV were selected to amplify and analyze the S, M, and N gene sequences. The results showed that the positivity rate of PRCV was 1.13% (71/6,267) using RT-qPCR. The homology analysis revealed that the nucleotide (amino acid) identity were 98.2–100% (96.6–100%) among the obtained 17 S, M, and N gene sequences, and 91.3–98.1% (88.8–98.0%) among the obtained strains and the reference strains from different countries. The phylogenetic analysis indicated that all PRCV strains could be divided into two groups, tentatively designated group I and group II, and each group was further divided into different clades. The strains obtained from Guangxi province distributed in group I, and formed an independent clade. They were most closely related to PRCV 137 isolate 86/135308 strain from the United Kingdom basing on the S, M, and N gene sequences. The sequence analysis revealed that all PRCV strains from Guangxi province obtained in this study showed 672 nt/224 aa deletion in the 5′ region of S gene, and there existed 37 amino acid mutations in different regions in S gene of different PCRV strains. Recombination analysis of S gene sequences did not find any recombination event. The Bayesian analysis indicated that all PRCV strains could be divided into Group I and II, and the obtained Guangxi strains belonged to group I. The population size of circulating PRCV strains kept slightly upward trend since its discovery in 1984 until 2010, showed a steady downward trend until 2020, and then a slight increase. The results suggested that the PRCV strains circulating in Guangxi province originated from Europe, and have evolved independently with genetic diversity. These findings enriched the epidemiological data of PRCV, and provided new information on the prevalence and genetic evolution of PRCV in Guangxi province of China.

## 1 Introduction

Six coronaviruses have been confirmed to cause diseases in pigs. They include porcine epidemic diarrhea virus (PEDV), porcine transmissible gastroenteritis virus (TGEV), porcine hemagglutinating encephalomyelitis virus (PHEV), porcine respiratory coronavirus (PRCV), porcine deltacoronavirus (PDCoV), swine acute diarrhea syndrome coronavirus (SADS-CoV) (Turlewicz-Podbielska and Pomorska-Mól, 2021). Of which, PRCV is the only coronavirus that has affinity to the respiratory system and cause respiratory disease. PRCV belongs to the *Alphacoronavirus* genus in the *coronaviridae* family (Woo et al., 2023). It was first discovered in Belgium in 1984 (Pensaert et al., 1986). Thereafter, PRCV has been found to be prevalent in different countries (Turlewicz-Podbielska and Pomorska-Mól, 2021), such as the United States of American (USA) (Vaughn et al., 1994; Wesley et al., 1997), Canada (Elazhary et al., 1992), Korea (Chae et al., 2000), Japan (Miyazaki et al., 2010), Germany (Kaden et al., 2009), Italy (Ferrara et al., 2022), Poland (Antas and Olech, 2024). PRCV antibody in serum samples from pigs was first reported in China in 1996 (Zhang et al., 1996). To date, PRCV has been reported in different provinces, such as Hebei, Jiangxi, Heilongjiang, Shandong, Hunan, and Guangxi provinces in China (Ding et al., 2025; Ma et al., 2024; Shi et al., 2025; Sun et al., 2023). Due to the increasing number of reports on PRCV in different countries, PRCV has gradually attracted more and more interest and attention.

PRCV is an enveloped, single-stranded, positive-sense RNA virus with approximately 28 kb genome size. It is recognized as a natural mutant of TGEV, with a deletion of 621–681 nucleotide (nt) in the 5′ region of S gene, and shows 96% genomic homology to TGEV (Turlewicz-Podbielska and Pomorska-Mól, 2021; Wesley et al., 1997). PRCV contains two large open reading frames (ORFs) at the 5′ end of the genome, ORF1a, and 1b, which encode nonstructural proteins pp1a and pp1ab. The remaining ORFs encode structural and non-structural proteins in the order as follows: spike (S), ORF3a, ORF3b, envelope (E), membrane (M), nucleocapsid (N), and non-structural proteins 7 (NP7) (Grellet et al., 2022; Turlewicz-Podbielska and Pomorska-Mól, 2021; Wesley et al., 1997). Among them, S, M, and N proteins act as important roles. S protein has the highest molecular weight and protrudes to form rods on the surface of the viral particle. S protein shows the functions of mediating the attachment of virions to the host cell receptor, involving in cell-to-cell fusion, inducing neutralizing antibodies, and bearing virulence determinants (Bosch et al., 2003; Beniac et al., 2006; Laude et al., 1993). M protein is an abundant protein. Its carboxyl terminus is embedded within the core of the viral particle, and this is essential for maintaining the core structure of the viral particle while mediating the transmembrane transport of nutrients (Kuo et al., 2016; Laude et al., 1993; Wong and Saier, 2021). N protein mediates to the viral genome and forms the nucleocapsid, a helical structure with a protective effect. This is essential for the formation of complexes with genome RNA during the assembly of the virus. Meanwhile, N protein is highly conserved and has strong immunogenicity, which is an important basis for the development of coronavirus vaccines and drugs (Cong et al., 2020; McBride et al., 2014). Therefore, S, M, and N genes are important research hotspots on molecular epidemiology for PRCV.

TGEV mainly causes gastrointestinal disease, which is characterized by acute diarrhea, vomiting and dehydration in pigs. Newborn piglets have a high mortality rate due to this disease (Chen et al., 2023a,b). Unlike TGEV, PRCV infection usually causes mild or subclinical respiratory infection symptoms. It shows little or no replication in the intestinal tract of infected pigs, and it usually enters the respiratory tract and replicates in large numbers, causing upper and lower respiratory tract disease (Ding et al., 2025; Wesley et al., 1997). It invades type 1 and type 2 pneumocytes, resulting in acute lung injury (Li et al., 2024a,b; Wesley et al., 1997). At the same time, macrophages and neutrophils infiltrate the alveoli, causing continuous irritation of the lung tissue, and eventually leading to severe interstitial pneumonia (Cox et al., 1990; Laude et al., 1993; Van Reeth et al., 1999). Therefore, the serious cases in the infected pigs usually show clinical signs of dyspnoea, sneezing, wheezing, coughing, fever, anorexia, and growth retardation (Halbur et al., 2003; Laude et al., 1993; Vannier, 1990; Wesley et al., 1997). Furthermore, co-infections of PRCV with other respiratory pathogens, such as swine influenza virus (SIV), and porcine reproductive and respiratory syndrome virus (PRRSV), can exacerbate the clinical symptoms and pathological changes, resulting in more severe economic losses (Bedsted et al., 2024a,b; Jung et al., 2009; Renukaradhya et al., 2010; Van Reeth and Pensaert, 1994).

Even if PRCV has been reported worldwide (Antas and Olech, 2024; Ferrara et al., 2022; Ma et al., 2024; Shi et al., 2025; Sun et al., 2023; Turlewicz-Podbielska and Pomorska-Mól, 2021), the information on phylogenetic analysis and genetic evolution of this virus is still limited. In China, there is even less information on this virus. In this study, the clinical samples were tested using RT-qPCR to investigate the prevalence of PRCV in Guangxi province in southern China; the S, M, and N genes of PRCV in positive samples were amplified and sequenced; Sequence comparison, phylogenetic analysis, recombinant analysis, and Bayesian temporal dynamics analysis were performed to explore genetic and evolutional characteristics of this virus, which enrichs the epidemiological data on PRCV.

## 2 Materials and methods

### 2.1 Clinical samples

Between July 2022 and March 2024, 6,267 clinical samples were collected from 14 death-pig harmless treatment plants, 15 pig farms, and 46 abattoirs in 14 cities in Guangxi province of China (Table 1). The clinical samples included 1,680 nasal swab samples, 4,587 tissue samples (each sample includes the trachea, lung, tonsil, and lymph node from the same pig, and was considered as one sample when tested) (Table 2). The collected samples were sent to the laboratory immediately after collection and stored at −80°C until use.

**Table 1:** The samples collected from different regions in Guangxi province.

**Region**	**Harmless treatment plant**		**Pig farm**		**Abattoir**		**Total**	
	**Sample**	**Positive (%)**	**Sample**	**Positive (%)**	**Sample**	**Positive (%)**	**Sample**	**Positive (%)**
Nanning	169	11 (6.51%)	200	0	2,047	33 (1.61%)	2,416	44 (1.82%)
Liuzhou	91	0	0	0	20	0	111	0
Guilin	0	0	0	0	94	0	94	0
Wuzhou	0	0	0	0	20	0	20	0
Beihai	0	0	0	0	20	0	20	0
Yulin	198	0	51	0	244	0	493	0
Guigang	0	0	41	0	80	0	121	0
Qinzhou	0	0	0	0	20	0	20	0
Laibin	0	0	140	12 (8.57%)	260	2 (0.77%)	400	14 (3.50%)
Hezhou	56	1 (1.79%)	150	4 (2.67%)	537	0	743	5 (0.67%)
Baise	0	0	37	0	660	8 (1.21%)	697	8 (1.15%)
Hechi	0	0	0	0	56	0	56	0
Congzuo	0	0	0	0	777	0	777	0
Fangchenggang	0	0	0	0	299	0	299	0
Total	514	12 (2.33%)	619	16 (2.58%)	5,134	43 (0.84%)	6,267	71 (1.13%)

**Table 2:** The different kinds of samples collected in Guangxi province.

**Region**	**Nasal swab**		**Tissue**		**Total**	
	**Sample**	**Positive (%)**	**Sample**	**Positive (%)**	**Sample**	**Positive (%)**
Nanning	1,345	28 (2.08%)	1,071	16 (1.49%)	2,416	44 (1.82%)
Liuzhou	0	0	111	0	111	0
Guilin	0	0	94	0	94	0
Wuzhou	0	0	20	0	20	0
Beihai	0	0	20	0	20	0
Yulin	0	0	493	0	493	0
Guigang	0	0	121	0	121	0
Qinzhou	0	0	20	0	20	0
Laibin	140	12 (8.57%)	260	2 (0.77%)	400	14 (3.50%)
Hezhou	195	5 (2.56%)	548	0	743	5 (0.67%)
Baise	0	0	697	8 (1.15%)	697	8 (1.15%)
Hechi	0	0	56	0	56	0
Congzuo	0	0	777	0	777	0
Fangchenggang	0	0	299	0	299	0
Total	1,680	45 (2.68%)	4,587	26 (0.57%)	6,267	71 (1.13%)

The tissue samples were put into 2.0 mL EP tubes with 1.0 mL of phosphate buffer solution (PBS, pH 7.2). The tissues were homogenized, frozen-and-thawed 3 times, and then centrifuged (12,000 rpm/min, 4°C) for 5 min. The nasal swab samples were put into 2.0 mL EP tubes with 1.0 mL of PBS (pH7.2), vortexed for 30 s, and centrifuged (12,000 rpm/min, 4°C) for 2 min. The nucleic acids were extracted from 200 μL supernatants of clinical samples using MiniBEST Viral DNA/RNA Nucleic Acid Extraction Kit Ver5.0 (TaKaRa, Dalian, China), and stored at −80°C until use.

The extracted nucleic acids were used to detect PRCV using the previously reported RT-qPCR established in our laboratory (Ma et al., 2024). Then, basing on their source from different cities in Guangxi province, sampling time in different seasons of year, and detection Ct values which were ≤ 30, the PRCV positive samples were selected to perform sequence analysis.

### 2.2 Primer design

To amplify and analyze the PRCV S, M, and N gene sequences, the PRCV genome sequences of the representative strains of PRCV were downloaded from NCBI GenBank (https://www.ncbi.nlm.nih.gov/nucleotide, accessed on 20 August 2021). The multiple sequence alignments were preformed, and the specific primers were designed for amplification of the whole gene sequences of PRCV S, M, and N genes (Table 3).

**Table 3:** The specific primers used to amplify PRCV S, M, and N genes.

**Gene**	**Primer**	**Primer sequence (5^′^ → 3^′^)**	**Product size (bp)**	**Annealing temperature (°C)**
S	PRCV–S1–F	CAAGTGTCGTTGTAACAACGC	874	54
	PRCV–S1–R	ACGCTTCAGTGTACGATGTGT		
	PRCV–S2–F	TGACCACTGGTGATAGTGACG	858	53
	PRCV–S2–R	GCGTTACAGAGTAGATAACACCAT		
	PRCV–S3–F	ACAGGACACTACTTAGTGGCTTA	866	54
	PRCV–S3–R	GTCAGCTATGTCATAACCACCT		
	PRCV–S4–F	GGTTGTAACATCTGGTTTAGGTAC	926	53
	PRCV–S4–R	CCACTCTAGGCTGATACATAGT		
	PRCV–S5–F1	ACTTGTCGTTAAAGATGTCCAG	511	53
	PRCV–S5–R1	ACCTGTACTACAACAGCAAAAT		
	PRCV–S5–F2	ACTTGCCATTCTYATTGACAAC	736	53
	PRCV–S5–R2	ATGCTGAACTCTGGGKAATAGT		
M	PRCV–M–F	ATGGAGCACTCCTTGTTTGAACT	858	59
	PRCV–M–R	CCAACTAACACGTTGTCCCT		
N	PRCV–N1–F	CTCAACAGAGGCAAGAACTGA	718	54
	PRCV–N1–R	TTTAGAACGAGAGCGTTGCTG		
	PRCV–N2–F	AGGATGACAGTGTAGAACAAGCT	617	54
	PRCV–N2–R	CATGGAGGAGGACGAGCAT		

In the degenerated primers, Y represents T or C, and K represents T or G.

### 2.3 Amplification and sequencing of S, M, and N genes

A total of 17 PRCV positive samples, which were detected using the established RT-qPCR (Ma et al., 2024), were selected for gene sequence analysis. The extracted nucleic acids were reverse transcribed into cDNA using PrimeScript II 1st Strand cDNA Synthesis Kit (TaKaRa, Dalian, China). The cDNAs were used as templates to amplify the PRCV S, M, and N genes. The total volume of the amplification system was 50 μL, which included 25 μL of Premix Taq™ (TaKaRa, Dalian, China), 1 μL of forward/reverse primer each (25 μM), 5 μL of viral cDNA, and 18 μL of distilled water. The amplification procedure was 94°C for 2 min; 35 cycles of 98°C for 10 s, 53°C−59°C (S2/S4/S5: 53°C; S1/S3/N1/N2: 54°C; M: 59°C) for 20 s, and 72°C for 60 s; 72°C for 10 min.

The PCR products were confirmed using electrophoresis, and purified using MiniBEST Nucleic Acid Extraction Kit (TaKaRa, Dalian, China). The purified products were ligated into pMD18–T Vector (TaKaRa, Dalian, China) at 16°C for 12–14 h, then transformed into *DH5*α competent cells (TaKaRa, Dalian, China) and cultured at 37°C in a shaking incubator with 500 rpm for 1 h. Then, 100 μL of the bacterial solution was coated to a LB solid medium and cultured at 37°C for 20–24 h. The positive clones were cultured in LB solution, and sent for sequencing (IGE Biotechnology, Guangzhou, China). The fragment sequences were spliced, and the whole S, M, and N gene sequences were obtained, then further verified through NCBI BLAST analysis (https://blast.ncbi.nlm.nih.gov/Blast.cgi, accessed on 1 April 2024).

### 2.4 Homology analysis of S, M, and N genes

The PRCV S, M, and N gene sequences of the reference strains were downloaded from NCBI GenBank (https://www.ncbi.nlm.nih.gov/nucleotide, accessed on 15 April 2025) (Supplementary Table 1). Only the strains that their full sequences of S, M, and/or N genes could be obtained were included in this study, and were used as the representative strains for reference strains to perform sequence comparison, phylogenetic analysis, recombinant analysis, and Bayesian temporal dynamics analysis. The S, M, and N gene sequences of the obtained strains in this study (Supplementary Table 2), together with those of the reference strains, were imported into the Bioedit v.7.2.5 software (https://bioedit.software.informer.com/download, accessed on 15 April 2025) for nucleotide (nt) and amino acid (aa) homology analysis.

### 2.5 Phylogenetic analysis of S, M, and N genes

The PRCV S, M, and N gene sequences of the representative strains were downloaded from NCBI GenBank (https://www.ncbi.nlm.nih.gov/nucleotide, accessed on 15 April 2025), and used as reference sequences (Supplementary Table 1). The DNAstar 7.0 software (https://www.dnastar.com/software, accessed on 15 April 2025) was used to compare the reference sequences with the obtained PRCV S, M, and N gene sequences (Supplementary Table 2). The accession numbers of the reference sequences and the obtained sequences in this study are provided in the Supplementary Tables 1, 2. The comparison results were included in the MEGA X 10.2.6 software (https://www.megasoftware.net/archived_version_active_download, accessed on 15 April 2025). The phylogenetic trees were constructed after 1,000 bootstrap replicates using the maximum likelihood method, then visualized using the Interactive Tree of Life (iTOL) (https://itol.embl.de, accessed on 15 April 2025) to access optimal details of phylogenetic trees.

### 2.6 Sequence analysis of S gene

The nucleotide and deduced amino acid sequences of PRCV S gene were analyzed using Bioedit v.7.2.5 software (https://bioedit.com, accessed on 15 April 2025). The PRCV 137 isolate 86/135308 strain (GenBank accession number: OM830320.1) and TGEV Virulent Purdue strain (GenBank accession number: DQ811789.2) (Supplementary Table 3) were selected as representative strains, which were used to compare with the obtained strains in this study for analyzing the variation sites in S gene.

In addition, the selection pressure analysis of the PRCV S gene was performed using datamonkey software (http://www.datamonkey.org, accessed on 8 June 2025). There were four models for site selection pressure analysis, including Mixed Effects Model of Evolution (MEME), Fixed Effects Likelihood (FEL), Fast Unconstrained Bayesian App Roximation (FUBAR), and Single-Likelihood Ancestor Counting (SLAC). When at least three models in these four methods had the same results, and the *p*-value of MEME, FEL and SLAC was 0.1 and the *p*-value of FUBAR was 0.9, the site was considered as at the selection state.

### 2.7 Recombination analysis

The obtained S, M, and N gene sequences, together with those of 43 PRCV reference sequences (Supplementary Table 1), were used to analyze possible recombination events. Seven algorithms in the Recombination Detection Program (RDP4) software (https://www.bioinf.manchester.ac.uk/recombination/programs, accessed on 15 April 2025) were employed in the detection procedure, including RDP, Bootscan, Chimaera, GENECONV, MaxChi, SiScan, and 3Seq. Only when at least five of these methods were supported could a recombination event be considered possible. The significance of the recombination event was further assessed by the *P*-value to determine whether recombination was indeed present. Then, the SimPlot 3.5.1 software (https://github.com/Stephane-S/Simplot PlusPlus, accessed on 15 April 2025) was further used to validate the potential recombination sequences.

### 2.8 Bayesian temporal dynamics analysis

The reference sequences were analyzed using MEGA X 10.2.6 software (https://www.megasoftware.net/archived_version_active_download, accessed on 15 April 2025). The obtained sequences were aligned using BEAST v1.10.4 software (http://beast.community, accessed on 15 April 2025) with the method of Bayesian clustering analysis, according to the best models of GTR, the uncorrelated relaxed clock and the Bayesian skyline. Bayesian Markov chain Monte Carlo (MCMC) was used to determine the gene dispersion time, and the number of iterations was set to 200 million steps, with a burn-in of 10%. In Tracer v1.6 software (https://github.com/beast-dev/tracer/releases/latest, accessed on 15 April 2025), the convergence of all parameters was determined to be ESS>200. Finally, the maximum clade credibility (MCC) was visualized using FigTree software (http://beast.community/figTree, accessed on 15 April 2025) to analyze the evolutionary relationship and temporal dynamics between sequences.

## 3 Results

### 3.1 Detection results of the clinical samples

**Figure 1:**
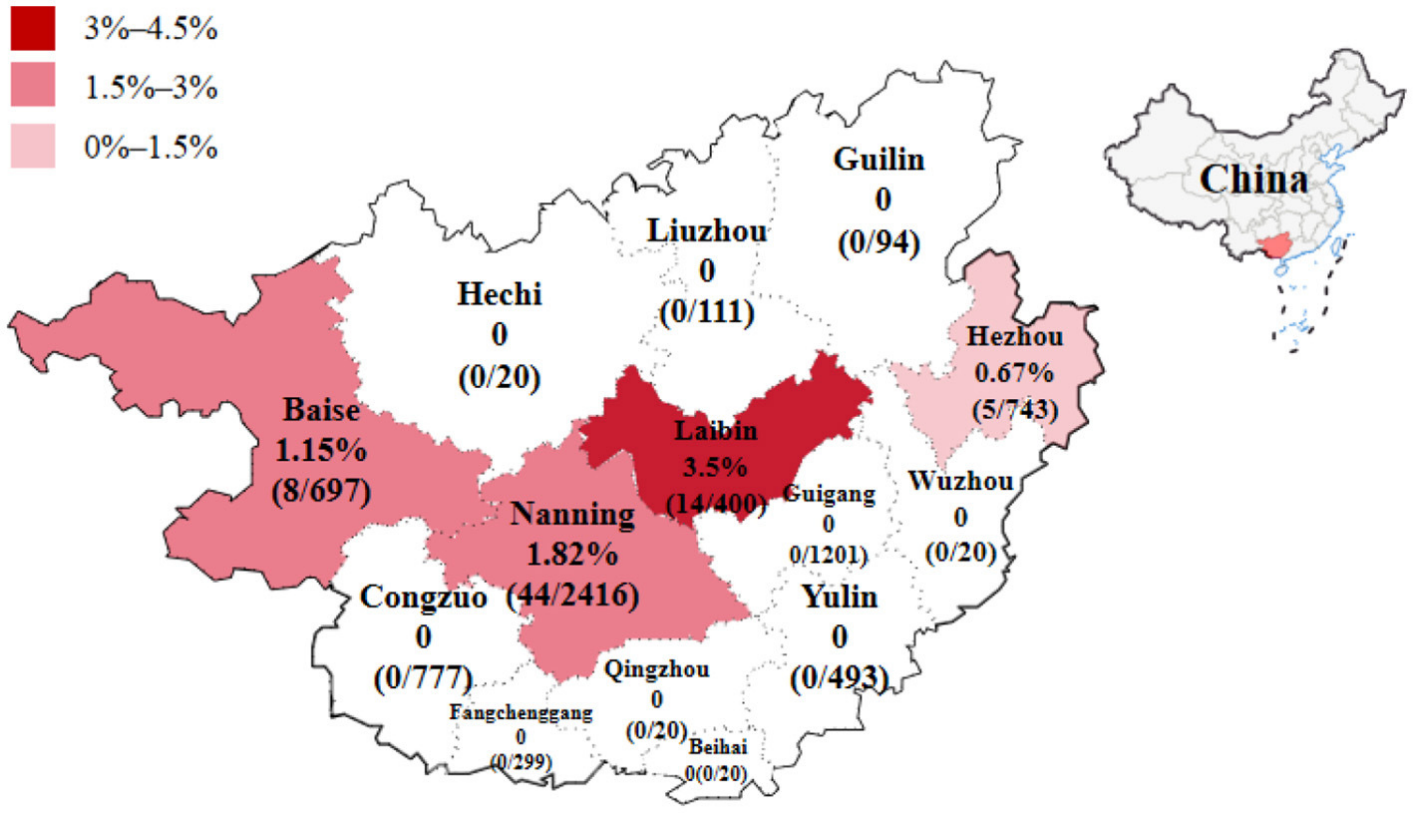
The distribution of PRCV positive samples in different regions of Guangxi province in China.

The 6,267 clinical samples collected from Guangxi province during 2022–2024 were tested for PRCV using the reported RT-qPCR (Ma et al., 2024). The results indicated that 71 samples were positive for PRCV, with a detection rate of 1.13% (71/6,267). Of the positive samples, 45 (45/1,680, 2.68%) samples were nasal swab samples, and 26 (26/4,587, 0.57%) samples were tissue samples; 16 (16/619, 2.58%) samples came from pig farms, 12 (12/514, 2.33%) samples came from harmless treatment plants, and 43 (43/5,134, 0.84%) samples came from abattoirs (Table 4). The positive samples came from Laibin, Nanning, Baise, and Hezhou city in Guangxi province, with a positivity rate of 3.50%, 1.82%, 1.15%, and 0.67%, respectively (Tables 1, 2). The distribution of PRCV positive samples in different regions of Guangxi province is shown in Figure 1.

**Table 4:** The PRCV positive samples collected from different sources in Guangxi province.

**Source**	**Nasal swab**		**Tissue**		**Total**	
	**Sample**	**Positive (%)**	**Sample**	**Positive (%)**	**Sample**	**Positive (%)**
Harmless treatment plant	51	7(13.73%)	463	5 (1.08%)	514	12 (2.33%)
Pig farm	342	11 (3.22%)	277	5 (1.81%)	619	16 (2.58%)
Abattoir	1,287	27 (2.10%)	3,847	16 (0.42%)	5,134	43 (0.84%)
Total	1,680	45 (2.68%)	4,587	26 (0.57%)	6,267	71 (1.13%)

A total of 17 PRCV positive samples were used to amplify and sequence S, M, and N genes. Finally, 17 S (3,678 nucleotides in length), 17 M (789 nucleotides in length), and 17 N (1,149 nucleotides in length) gene sequences were obtained in this study, and submitted to GenBank under the accession numbers of PQ204794-PQ204810 for S gene, PQ204811-PQ204827 for M gene, and PQ204828-PQ204844 for N gene (Supplementary Table 2).

### 3.2 Homology of S, M, and N gene sequences

The S, M, and N gene sequences of the 17 obtained PRCV strains showed nucleotide (nt) and amino acid (aa) homology of 98.2–100% and 98.2–100%, 96.6–100% and 96.6–100%, 97.6–99.9% and 96.9–99.7% among the obtained strains, respectively. The obtained PRCV S, M, and N gene sequences showed nucleotide and amino acid homology of 93.1–97.0% and 93.5–98.0%, 93.1–98.1% and 90.5–97.7%, 91.3–96.3% and 88.8–94.8% with the reference strains, respectively. The S, M, and N genes showed the highest nucleotide (amino acid) homology of 97.0% (98.0%), 98.1% (97.7%), and 96.3% (94.8%), respectively, with the reference strain 137 isolate 86/135308 from the United Kindom (UK) (Table 5).

**Table 5:** Sequence homology among the obtained and reference sequences.

	**Sequence homology among the obtained strains**		**Sequence homology among the obtained strains and reference strains**	
	**nt**	**aa**	**nt**	**aa**
S	98.2%−100%	98.2%−100%	93.1%−97.0%	93.5%−97.7%
M	96.6%−100%	96.6%−100%	93.1%−98.1%	90.5%−97.7%
N	97.6%−99.9%	96.9%−99.7%	91.3%−96.3%	88.8%−95.0%

### 3.3 Phylogenetic analysis of S, M, and N gene sequences

#### 3.3.1 S gene

**Figure 2:**
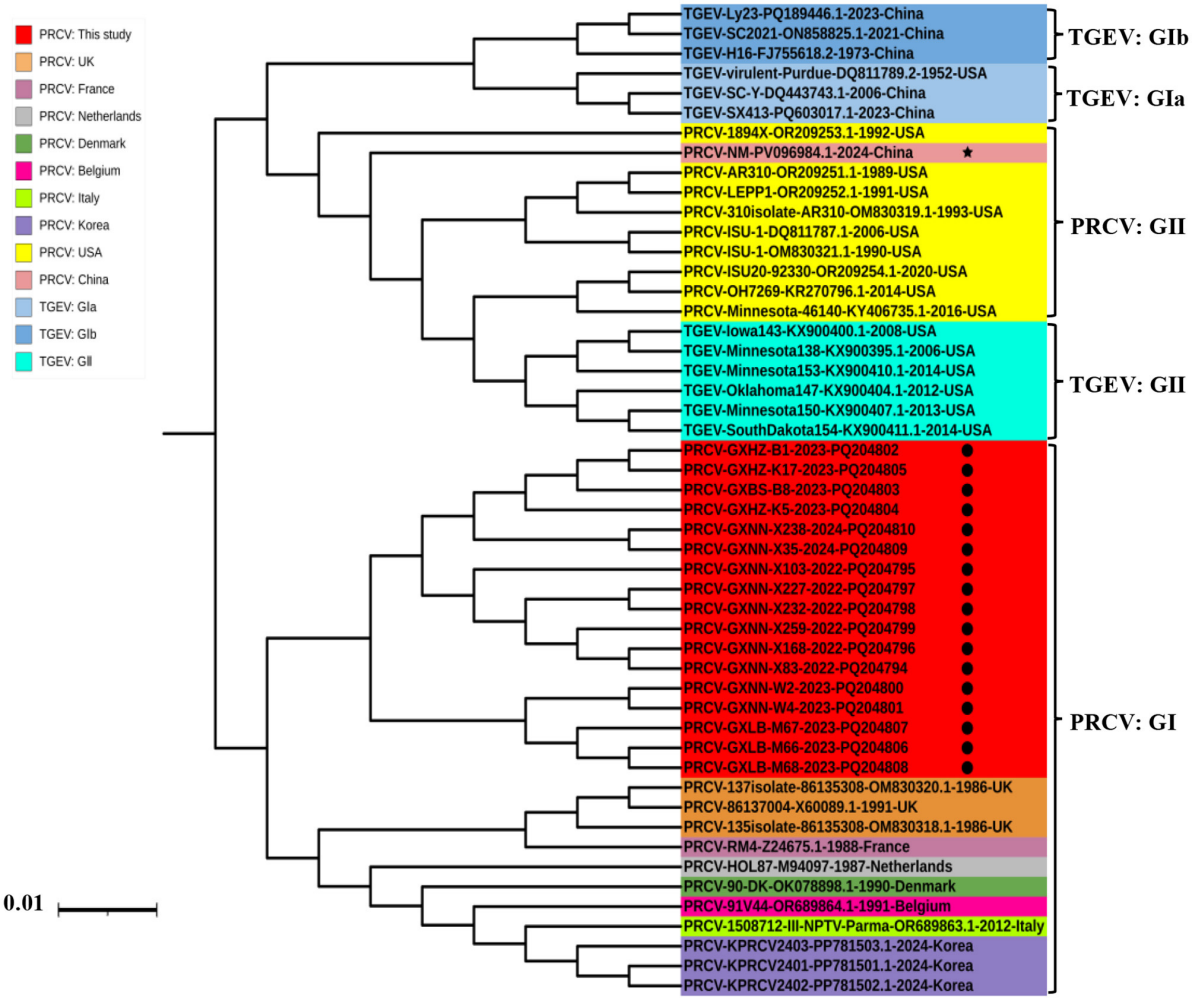
The phylogenetic tree basing on PRCV S gene nucleotide sequences. The black spots indicate the PRCV strains obtained in this study, and the black five-pointed star indicates another PRCV strain from China.

The phylogenetic tree was constructed basing on the S gene sequences of 17 obtained PRCV strains and the 21 PRCV reference strains from different countries (Figure 2). The results revealed that all the PRCV strains were divided into two groups, tentatively designated group I and group II. Of which, the PRCV strains from Europe and Asia distributed in group I, and the PRCV strains from America and China distributed in group II. The PRCV strains obtained in this study formed a separate clade, which belonged to group I (Figure 2). The Guangxi strains were more close to the European strains than to the American strains.

#### 3.3.2 M gene

**Figure 3:**
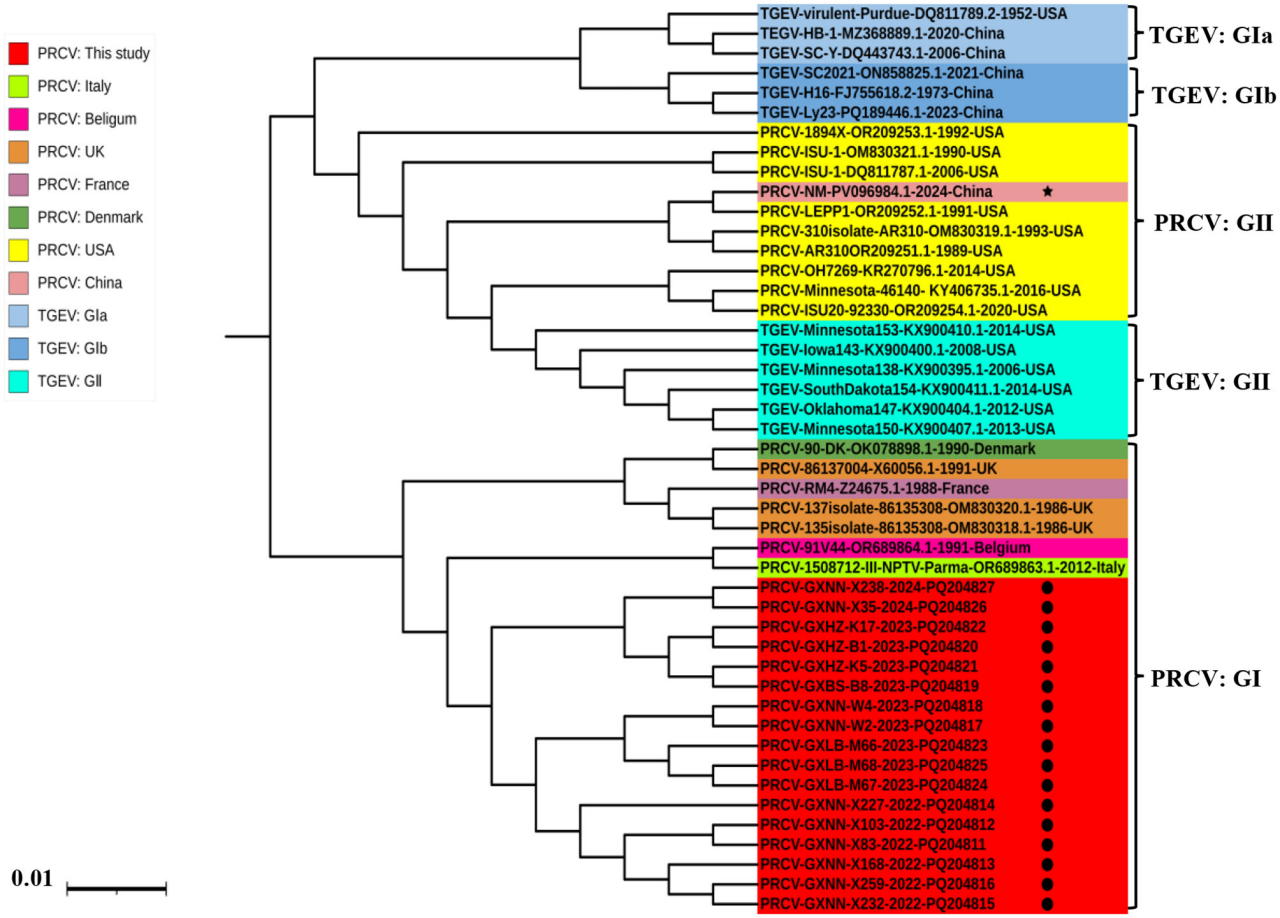
The phylogenetic tree basing on PRCV M gene nucleotide sequences. The black spots indicate the PRCV strains obtained in this study, and the black five-pointed star indicates another PRCV strain from China.

The phylogenetic tree was constructed basing on the M gene sequences of 17 obtained PRCV strains and 17 PRCV reference strains from different countries (Figure 3). The results revealed that all PRCV strains could be divided into two groups, tentatively designated group I and group II. Similar to that of S gene, the Guangxi strains belonged to group I, and were closely related to the European strains, with the closest genetic relationship to 91V44 strain (GenBank accession number: OR689864.1) from Belgian.

#### 3.3.3 N gene

**Figure 4:**
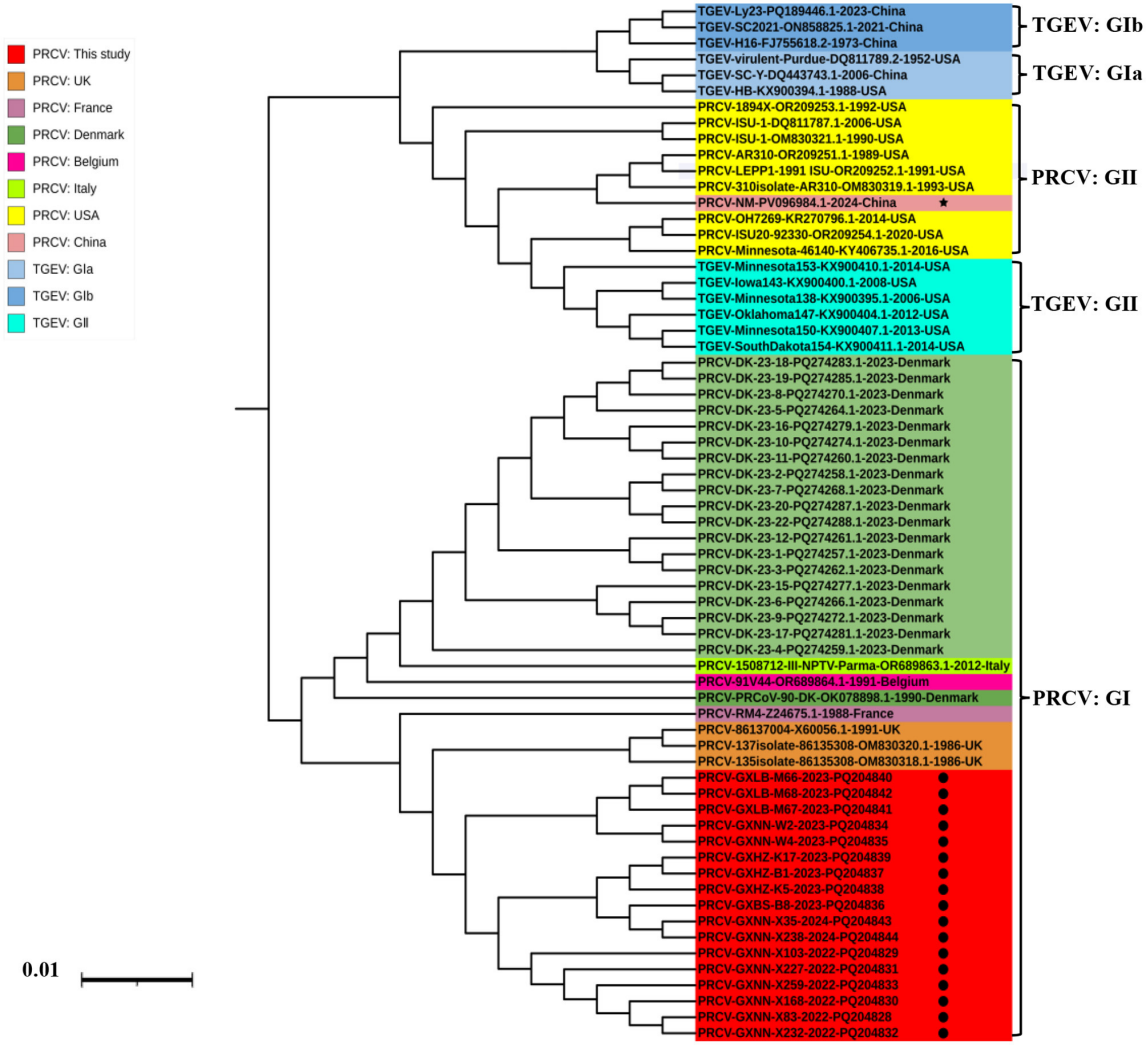
The phylogenetic tree basing on PRCV N gene nucleotide sequences. The black spots indicate the PRCV strains obtained in this study, and the black five-pointed star indicates another PRCV strain from China.

The phylogenetic tree was constructed basing on the N gene sequences of 17 obtained PRCV strains and 36 PRCV reference strains from different countries (Figure 4). The results demonstrated that all PRCV strains could be divided into two groups, tentatively designated group I and group II. The PRCV obtained strains in this study were formed a separate clade, which belonged to group I. The Guangxi strains were closely related to the European strains, with the closest to 137 isolate 86/135308 strain from UK.

### 3.4 Sequence analysis of S gene

#### 3.4.1 Sequence analysis of the 5' region in S gene

**Figure 5:**
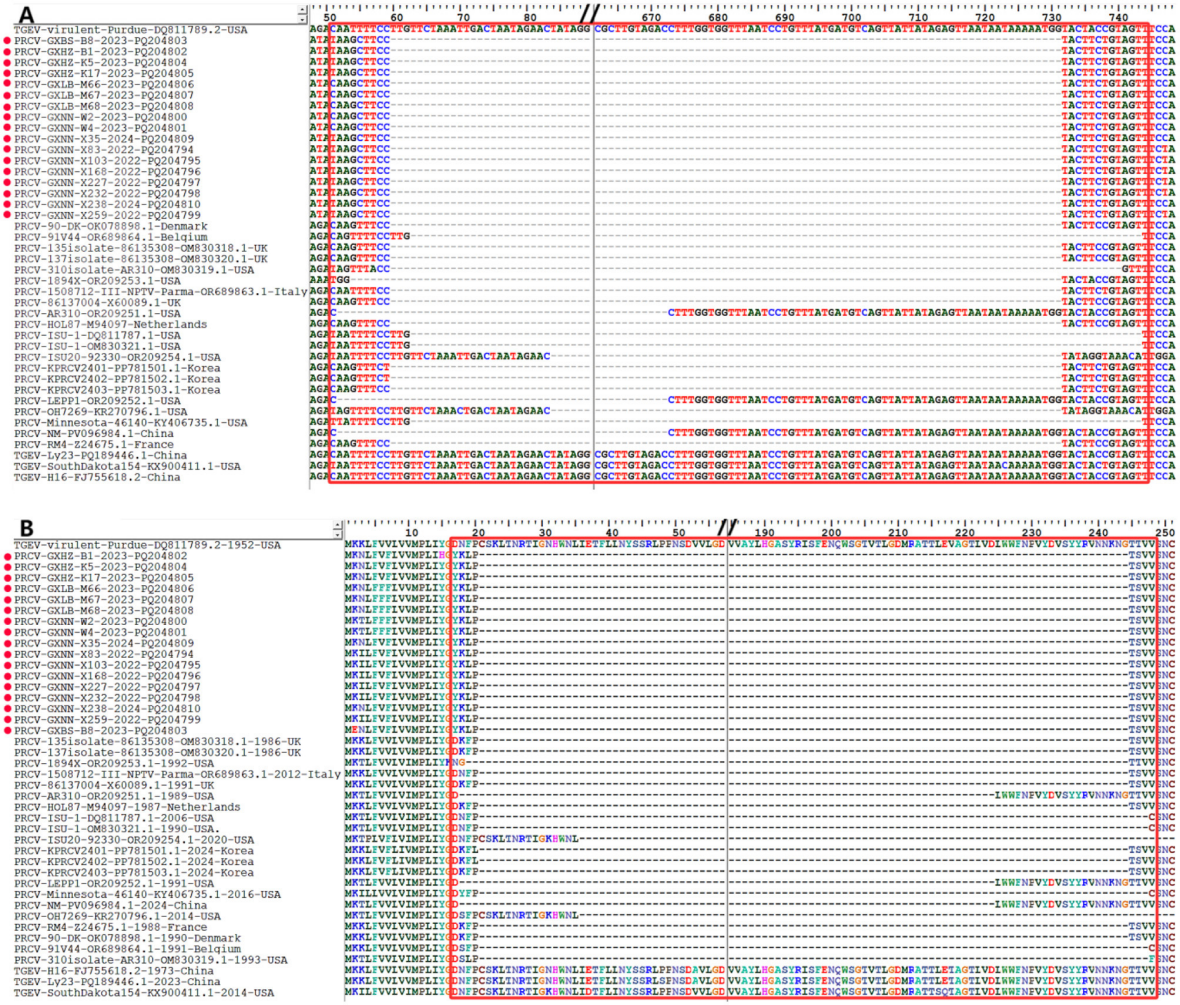
The deletion of nucleotides and amino acids in the 5′ region of PRCV S gene. **(A)** Nucleotide deletion in S gene. **(B)** Amino acid deletion in S gene. The red spots indicate the PRCV strains obtained in this study.

The S gene nucleotide and amino acid sequences of the 17 obtained PRCV strains and the 21 PRCV reference strains were compared with those of the S gene of the TGEV reference strain Virulent Purdue (GenBank accession number: DQ811789.2) using Bioedit software. The results showed that all PRCV strains had deletions of 621–681 nt (207–227 aa) between nucleotide sites (amino acid residue sites) of 52–743 nt (18–247 aa) compared with TGEV Virulent Purdue strain (Figure 5). All the obtained strains from Guangxi province in this study showed 672 nt (224 aa) deletion at the nucleotide sites (amino acid residue sites) of 60–731 nt (21–244 aa).

#### 3.4.2 Sequence analysis of S gene

The 137 isolate 86/135308 strain (GenBank accession number: OM830320.1) from UK was used as the representative strain for sequence analysis. The S gene nucleotide sequences of 17 obtained strains and the 21 reference strains were compared with those of 137 isolate 86/135308 strain. The results showed that all PRCV strains had mutations in different regions in S gene (Supplementary Figure 1). Of the 3,678 nucleotides in S gene, there were 153 nucleotide sites that more than four strains of the 17 obtained PRCV strains showed mutations (Supplementary Table 4), which resulted in 37 aa mutations in different regions in S gene (Supplementary Figure 2). The 37 mutation sites of amino acids in the 17 obtained strains are summarized in Table 6.

**Table 6:** The amino acid mutation information on S gene of the 17 obtained PRCV strains.

**Point**	**Mutation**	**Point**	**Mutation**	**Point**	**Mutation**
3	K → N/T/I	603	D → N	1,014	A → D
6	V → F	604	A → S	1,245	D → N
7	V → F	618	D → Y	1,286	V → I
17	D → Y	654	F → V	1,326	F → L
19	F → L	748	I → V/G	1,340	A → T
285	I → L	772	I → T	1,351	D → E
456	G → V	789	T → N	1,407	C → F
458	S → G	794	I → F	1,411	L → M/I
501	A → S	796	S → I	1,423	C → L/F
502	N → T	814	K → N	1,443	Y → F
505	N → D	927	E → D	1,445	P → L
562	M → V	934	K → N		
595	K → R/G	949	Y → H		

Under the influence of natural selection, evolution progresses toward adaptation to the environment. Therefore, the selection pressure of the S protein was investigated. The selection pressure analysis was performed using four models of MEME, FEL, FUBAR, and SLAC. The results indicated that no positive selection pressure site was detected using the SLAC model, while the positive selection pressure was detected by the MEME, FUBAR, and FEL models at the 18, 387, and 565 sites (Table 7).

**Table 7:** The analysis of positive selection pressure on the S protein of PRCV.

**Site**	***P*** **-value**		
	**MEME**	**FUBAR**	**FEL**
18	0.025	0.996	0.029
387	0.043	0.931	0.030
565	0.027	0.978	0.022

### 3.5 Recombination analysis

**Figure 6:**
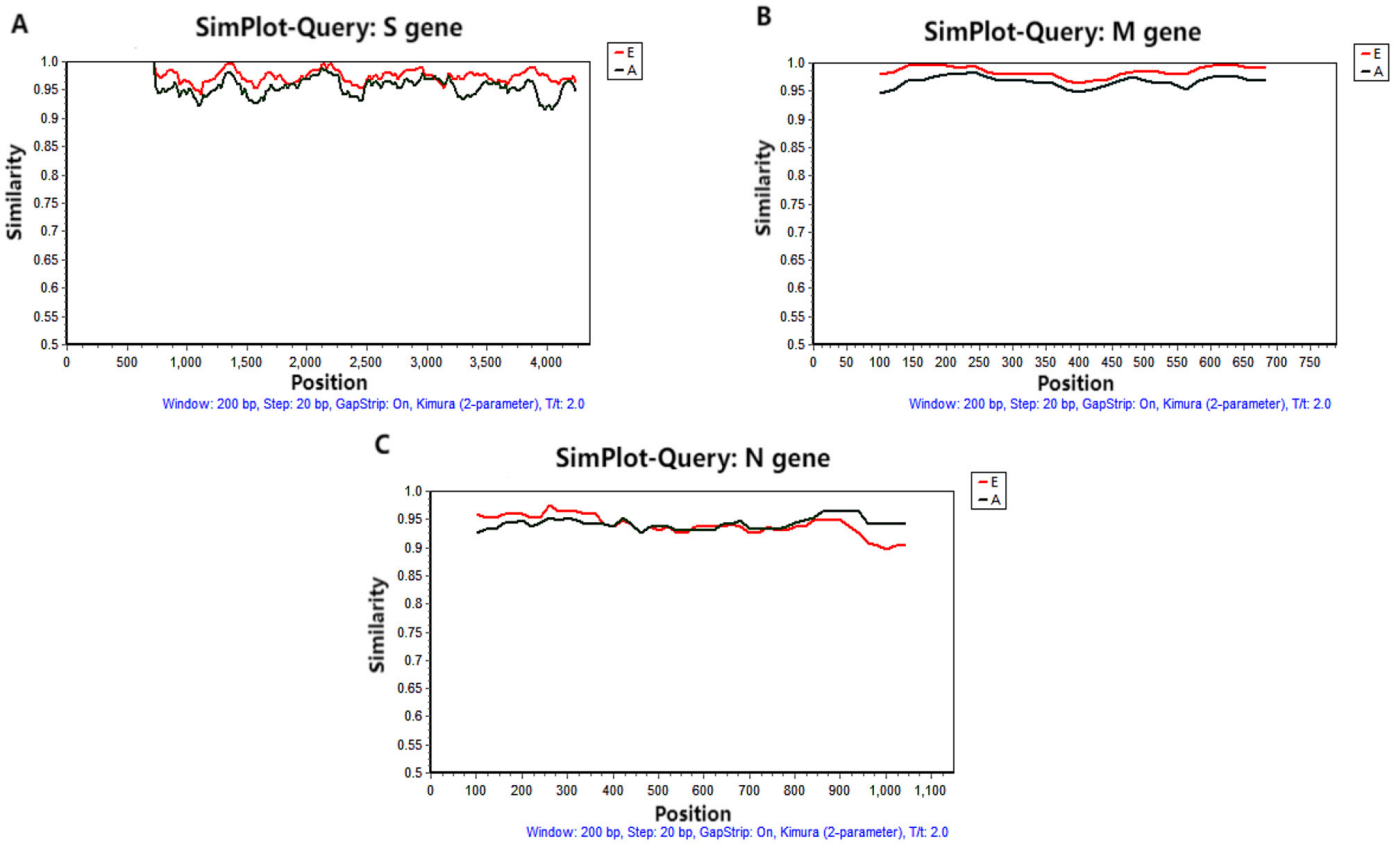
Recombination analysis of S **(A)**, M **(B)**, and N **(C)** genes. The red line is the European reference strain. The black line is the American reference strain.

Recombination analyses of the S, M, and N gene sequences of the 17 PRCV strains obtained in this study and the 43 PRCV reference strains did not find any significant recombination event (Figure 6).

### 3.6 Bayesian temporal dynamic analysis

**Figure 7:**
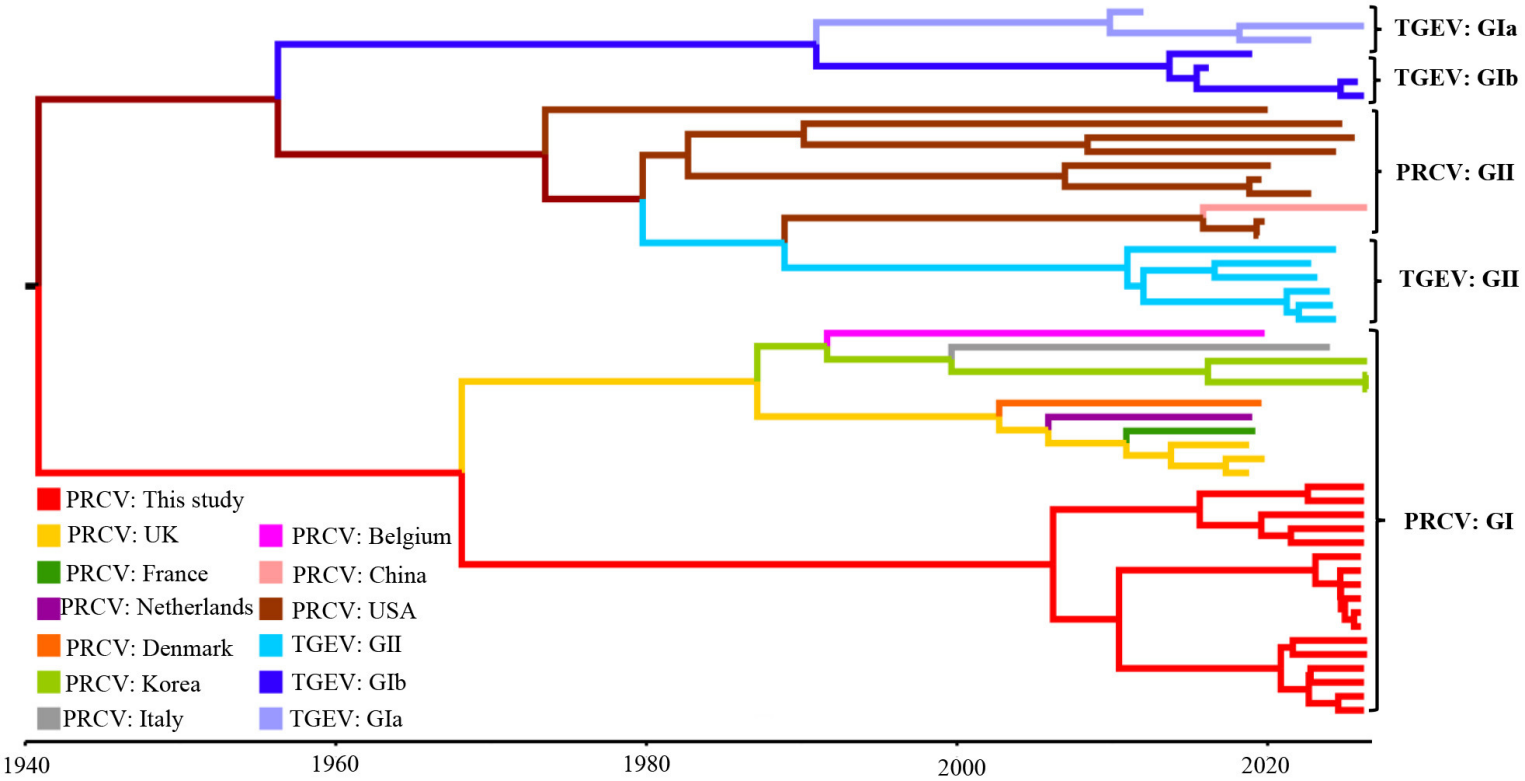
The Maximum clade credibility (MCC) tree of the PRCV S gene sequences.

The construction of the MCC tree of the S gene based on the temporal scale showed that all the PRCV strains were divided into two groups, tentatively designated group I and group II. The PRCV strains from Europe and Asia distributed in group I, and the PRCV strains from America and China distributed in group II. The PRCV strains obtained in this study belonged to group I, and formed an independent clade (Figure 7). The obtained strains were highly similar to the European PRCV strains, with the highest homology to the European strain 137 isolate 86/135308 from UK.

**Figure 8:**
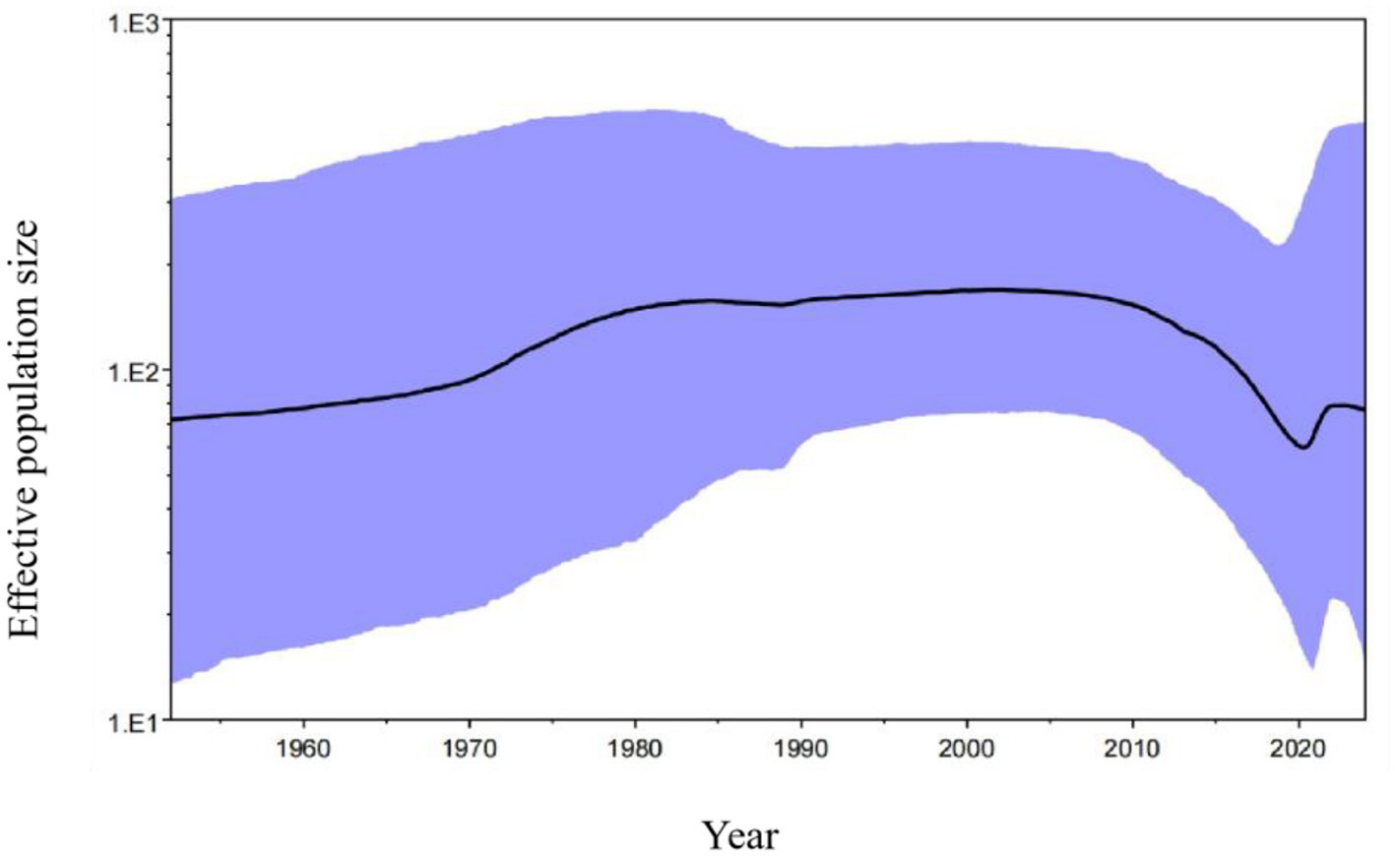
The Bayesian skyline of PRCV S gene. The black line indicates the median estimate of the effective population size, and the purple region indicates the corresponding 95% confidence interval.

Basing on the Bayesian skyline results, the PRCV effective population size expanded slowly after the first report in 1980s, and level off to 2010. Then, it shrank rapidly until about 2020, and appeared to level up in recent years (Figure 8).

### 3.7 Evolution rates of S, M, and N genes

The S, M, and N gene sequences of the obtained strains and the reference strains were analyzed using the uncorrelated relaxed clock, the GTR, HEY, GTR, and the Bayesian skyline models in the BEAST v1.10.4 software. The results demonstrated that the average evolutionary rates of S, M, and N genes were 3.68 × 10^−4^, 2.35 × 10^−4^, and 6.09 × 10^−4^ substitution/site/year (s/s/y), respectively (Table 8).

**Table 8:** The evolutionary rates of S, M, and N genes.

**Gene**	**Mean evolutionary rate (s/s/y)**	**95% HPD (s/s/y)**
S	3.68 × 10^−4^	2.02 × 10^−4^-5.55 × 10^−4^
M	2.35 × 10^−4^	1.30 × 10^−4^-3.47 × 10^−4^
N	6.09 × 10^−4^	4.34 × 10^−4^-7.78 × 10^−4^

## 4 Discussion

Since PRCV was first discovered in Belgium in 1984, it has been reported in many countries around the world (Antas and Olech, 2024; Ferrara et al., 2022; Ma et al., 2024; Shi et al., 2025; Sun et al., 2023; Turlewicz-Podbielska and Pomorska-Mól, 2021). In Asia, the pig-level and the farm-level seroprevalence of PRCV in Korea were 53.14% (237/446) and 61.36% (54/88) during 1998–1999, and 41.14% (144/350) and 68.57% (48/70) reported in 2024 (Chae et al., 2000; Kim et al., 2024). The pig-level and the farm-level seroprevalence of PRCV in Japan during 2005–2007 were 7.62% (206/2,703) and 15.20% (26/171), and the sera from a Japanese PRCV-positive pig farm showed 90.18% (101/112) positivity rate of antibodies against PRCV (Miyazaki et al., 2010; Usami et al., 2008). In Europe, the sera from wild boar populations in Germany during 1997–2005 showed a seroprevalence rate of 7.87% for PRCV (Kaden et al., 2009). The PRCV antibodies were detected in 0.67% (3/444) serum samples from wild boar in Italy during 2016–2017, and 0.91% (4/438) serum samples from domestic pigs in Italy reported in 2023 (Ferrara et al., 2022, 2023). The nasal swabs from 55 respiratory disease cases in Spain during 2017–2019 showed positive for PRCV in 52.73% (29/55) cases and 48.0% (419/873) individual pigs (Martín-Valls et al., 2022). The serum samples from Poland during 2021–2024 showed 12.20% (101/828) positivity rate for anti-PRCV antibodies, while the nasal swabs and stool samples showed 6.22% (31/498) positivity rate for PRCV RNAs (Antas and Olech, 2024). In Denmark, antibodies against PRCV were detected in 74.91% (191/255) of pig sera collected during 2021–2022, and PRCV nucleic acids were detected in 35.35% (117/331) in nasal swab samples collected during 2021–2023 (Bedsted et al., 2024a,b). In America, the serum samples from 22 pig herds in Iowa State of USA in 1995 showed a positivity rate of 59.37% (206/347) for PRCV antibodies (Wesley et al., 1997). PRCV RNA was detected in 0.40% (5/1,245) of the lung homogenate samples from pigs in USA in 2020 (Rawal et al., 2023). PRCV infection can lead to respiratory tract damage and cause other respiratory diseases, but the clinical signs are usually mild or subclinical, and the morbidity and mortality are relative low (Halbur et al., 2003; Keep et al., 2022; Laude et al., 1993; Turlewicz-Podbielska and Pomorska-Mól, 2021; Vannier, 1990). Therefore, relatively few research reports have been conducted on PRCV, while compared with other swine coronaviruses. However, co-infections of PRCV with other respiratory pathogens, such as PRRSV, and SIV, can exacerbate the clinical signs and pathological changes, resulting in more severe economic losses (Bedsted et al., 2024a,b; Jung et al., 2009; Renukaradhya et al., 2010; Van Reeth and Pensaert, 1994). Therefore, PRCV has gradually been received high attention in various countries in recent years.

PRCV was first reported in China in 1996 (Zhang et al., 1996). Since then, it has been reported in some provinces in China, including Hebei, Jiangxi, Heilongjiang, Shandong, Hunan, and Guangxi provinces (Ding et al., 2025; Ma et al., 2024; Shi et al., 2025; Sun et al., 2023). In this study, 6,267 clinical samples were collected from pig farms, harmless treatment plants, and abattoirs. The positivity rate of PRCV was 1.13% (71/6,267) using RT-qPCR for detection, showing a low prevalence in Guangxi province. These positive samples came from pig farms, harmless treatment plants, and abattoirs in four cities, i.e., Laibin, Nanning, Baise and Hezhou, in Guangxi province. The results of the present study are consistent with other previous reports (Antas and Olech, 2024; Bedsted et al., 2024a,b; Ferrara et al., 2022, 2023; Ma et al., 2024; Martín-Valls et al., 2022; Shi et al., 2025; Sun et al., 2023; Turlewicz-Podbielska and Pomorska-Mól, 2021). To date, there have few reports on PRCV in China (Ding et al., 2025; Ma et al., 2024; Shi et al., 2025; Sun et al., 2023; Zhang et al., 1996), so limited information is known about the prevalence of PRCV in China. The results in this study confirmed that PRCV is circulating in pig herds in China, and further study is necessary to obtain more detailed and accurate information on the prevalent situations.

The S, M, and N genes of 17 PRCV positive samples were amplified and sequenced. Then, homology analysis, phylogenetic analysis and recombination analysis were performed basing on the obtained gene sequences and the downloaded reference sequences. The sequence analysis showed that all PRCV strains have 621–681 nt/207–227 aa deletion in the 5′ region of S gene while compared with TGEV S gene. The PRCV strains obtained in this study had 672 nt/224 aa deletion in the 5′ region of S gene, and had 37 aa mutations at different regions of S gene. These results are consistent with other strains from different countries (Antas and Olech, 2024; Bedsted et al., 2024a,b; Kim et al., 2000, 2024; Rasschaert et al., 1990; Rawal et al., 2023; Vaughn et al., 1994; Wang and Zhang, 2017). The obtained PRCV S, M, and N gene sequences showed high homology of 97.6%−100% (nt)/96.6%−100%% (aa), and have homology of 91.3%−98.1%% (nt)/88.5%−97.7% (aa) with the reference strains from different countries, indicating that the circulating PRCV strains showed genetic diversity in pig herds. No recombination event was found in the S, M, and N gene sequences, suggesting that recombination might not be the main mechanism for genetic variation in PRCV. The average evolution rates of S, M, and N genes were estimated as 3.68 × 10^−4^, 2.35 × 10^−4^, and 6.09 × 10^−4^ s/s/y, indicating that the evolution rates of three genes fluctuate with highly concordant amplitudes with relatively slow genetic evolution. In the previous reports, the average evolution rates of S/M/N genes of PDCoV, PEDV, and PHEV in Guangxi province were estimated as 1.91 × 10^−3^/8.32 × 10^−4^/1.14 × 10^−3^, 1.53 × 10^−3^/1.52 × 10^−4^/1.08 × 10^−3^, and 2.66 × 10^−4^/5.44 × 10^−4^/3.11 × 10^−4^ s/s/y, respectively (Li et al., 2024a,b; Shi et al., 2024a,b), indicating that PRCV was relative stable compared with other swine coronaviruses of PDCoV and PEDV, while was similar to PHEV. The relative evolutionary stability of PRCV might be attributed to the relatively low immune pressure. At present, no commercial vaccine for PRCV is available, and no vaccination has been performed. The prevalence rate of natural infection of PRCV is still relatively low (Ma et al., 2024), and the proportion of pigs with antibodies against PRCV in the pig population is very low in Guangxi province. Therefore, the PRCV keep relatively stability due to the low immune pressure.

The phylogenetic analysis revealed that all PRCV strains could be divided into two groups (I, and II), and this is the first time to tentatively designate all PRCV strains into two groups, and further divide into different clades. The TGEV strains can be divided into groups I (subgroup Ia, and Ib), and II (Chen et al., 2023a,b), and the PRCV strains can be referred to the grouping of TGEV and be divided into groups I, and II. Other scientists have reported similar results previously (Antas and Olech, 2024; Bedsted et al., 2024a,b; Chen et al., 2019; Ding et al., 2025; Rawal et al., 2023; Sun et al., 2023). The strains from America and China distributed in group II, the strains from Europe distributed in group I, and the strains from Asia distributed in both group I and group II. Interestingly, the obtained strains were closely related to the European strains, and formed a separate clade, indicating that the strains from Guangxi province might be originally derived from Europe, evolved independently after introducing into Guangxi province, and showed geographically evolutional characteristics. Guangxi province is located in South China and borders Vietnam. The live pigs raised in Guangxi province can not only meet the needs of the province, but also be exported to other provinces, with almost no live pigs imported from abroad or other provinces in China (except for breeding pigs). Therefore, after the virus is introduced, it almost spreads in a closed loop within the Guangxi pig population. In addition, Guangxi province is located in the subtropical region, with hot, rainy and humid weather throughout the year, and has its unique climate characteristics. After introduction, PRCV are usually circulate and evolve in a closed loop in Guangxi region for a long time, forming unique genetic evolution characteristics and possessing unique genomic features. Such independent evolution of PRCV has also been reported in Denmark, Japan and Korea (Bedsted et al., 2024a,b; Kim et al., 2024; Usami et al., 2008). It is noteworthy that one strain from China (NM Strain, GenBank accession number: PV096084.1) distributed in group II, suggesting that Chinese strain might also be derived from America. This is consistent with the source of imported breeding pigs from abroad in China. China has introduced breeding pigs from both America and Europe, and PRCV might accompany the transmission of breeding pigs from America and Europe to China, resulting in the complexity of the PRCV strains circulating in China. In the previous reports, the PRCV strains from Korea and Japan were closely related to the European strains and distributed into the same clade (Kim et al., 2024; Usami et al., 2008), which is similar to the situation of Guangxi strains obtained in this study.

The Bayesian analysis revealed that all PRCV strains could be divided into groups I and II, which was similar to the results of the constructed phylogenetic trees. The PRCV strains from Guangxi province formed a separate clade, and might be a newly emerged clade. The PRCV strains circulating in Guangxi province had geographically evolutional characteristics, which is similar to other swine coronaviruses of PDCoV, PEDV, and PHEV (Bai et al., 2023; Li et al., 2024a,b; Shi et al., 2024a,b). The Bayesian skyline analysis revealed that the PRCV in Guangxi province has also spread since it was discovered in Belgium in 1984, and slowly upward trend until 2010, showed a steady downward trend until 2020, and followed by a slight increase afterward. These results had similarities with the results of the Bayesian skyline performance mapped in the previous reports for PDCoV, PEDV, and PHEV in Guangxi province (Bai et al., 2023; Li et al., 2024a,b; Shi et al., 2024a,b), and for PDCoV and PEDV in other provinces of China (He et al., 2020, 2022; Yan et al., 2022).

At present, no commercial vaccine against PRCV is available. Since PRCV usually cause mild or subclinical respiratory infection and low mortality (Halbur et al., 2003; Vannier et al. 1990; Wesley et al., 1997), which leads to less attention and research on PRCV. The occurrence of novel coronaviruses (COVID-2019) in humans since the end of 2019 has led to a renewed increase in attention to coronaviruses as pathogens (Choi et al., 2025; Embrett et al., 2025). PRCV, known as the only swine coronavirus to cause respiratory disease, has attracted more and more attention. The epidemiological surveillance and understanding of genetic characteristics of PRCV is great significant to prevent and control this disease. This study enriches the molecular epidemiological data on PRCV in Guangxi province, which is helpful for the subsequent study of the pathogenesis of PRCV, vaccine development, and prevention and control strategies. It is necessary to continuously monitor and track the prevalence and genetic variation of PRCV in order to effectively prevent and control PRCV.

## 5 Conclusions

PRCV is the only swine coronavirus that has affinity to the respiratory system and induces respiratory disease. PRCV showed a positive rate of 1.13% in the clinical samples from Guangxi province during 2022–2024. The PRCV strains from Guangxi province had 472 nt/224 aa deletion in the 5′ region of S gene, and had variations in some regions in S gene. All PRCV strains from different countries were divided into group I and group II. All the obtained strains from Guangxi province distributed in group I, and formed a separate clade. PRCV effective population size expanded slowly from 1980s to 2010, and showed a slow shrinking trend until about 2020, and then appeared to level up in recent years. This study enriches the molecular epidemiological data on PRCV in Guangxi province, which is helpful for the subsequent study on pathogenesis, vaccine development, and prevention and control strategies of PRCV. To the best of our knowledge, this is the first report on the molecular epidemiology and genetic evolution on PRCV in South China. This research provides new insights into the prevalence and genetic diversity of PRCV, and suggests the urgent need for viral surveillance.

## Data Availability

The datasets presented in this study can be found in online repositories. The names of the repository/repositories and accession number(s) can be found in the article/Supplementary material.
