# Supplementary material for: Phylogenetic analysis and genetic evolution of porcine respiratory coronavirus in Guangxi province, Southern China from 2022 to 2024
Source: Front Microbiol. 2025 Jul 10;16:1625343. doi: 10.3389/fmicb.2025.1625343 (PMC12287051; doi:10.3389/fmicb.2025.1625343)
Supplement: Supplementary file 1 [file Data_Sheet_1.docx]

| Majority ATGAAAAAATTATTTGTGTTCTTGGTTGTAATGCCATTGATTTATGGAGACAAGTTTCCXXXXXXXXXXXXXXXXXXXXXXXXXXXXXXXXXXXXXXXXXXXXXXXXXXXXXXXXXXXXXXXXXXXXXXXXXXXXXXXXXXXXXXXXXXXXX |
| --- |
| ---------+---------+---------+---------+---------+---------+---------+---------+---------+---------+---------+---------+---------+---------+---------+-- |
| 10 20 30 40 50 60 70 80 90 100 110 120 130 140 150 |
| ---------+---------+---------+---------+---------+---------+---------+---------+---------+---------+---------+---------+---------+---------+---------+-- |
| PRCV-137isolate-86135308-OM830320.1-UK ..................G........................................--------------------------------------------------------------------------------------------- 152 |
| PRCV-GXBS-B8-2023-PQ204803 ...G....T........C..............................T.T...C....--------------------------------------------------------------------------------------------- 152 |
| PRCV-GXHZ-B1-2023-PQ204802 ........T........C........................C.....T.T...C....--------------------------------------------------------------------------------------------- 152 |
| PRCV-GXHZ-K5-2023-PQ204804 ........T........C..............................T.T...C....--------------------------------------------------------------------------------------------- 152 |
| PRCV-GXHZ-K17-2023-PQ204805 ........T........C..............................T.T...C....--------------------------------------------------------------------------------------------- 152 |
| PRCV-GXLB-M66-2023-PQ204806 ........T......T.C..T...........................T.....C....--------------------------------------------------------------------------------------------- 152 |
| PRCV-GXLB-M67-2023-PQ204807 ........T......T.C..TC..........................T.....C....--------------------------------------------------------------------------------------------- 152 |
| PRCV-GXLB-M68-2023-PQ204808 ........T......T.C..T...........................T.....C....--------------------------------------------------------------------------------------------- 152 |
| PRCV-GXNN-W2-2023-PQ204800 .......CT......T.C..T...........................T.....C....--------------------------------------------------------------------------------------------- 152 |
| PRCV-GXNN-W4-2023-PQ204801 .......CT......T.C..T...........................T.....C....--------------------------------------------------------------------------------------------- 152 |
| PRCV-GXNN-X35-2024-PQ204809 ........T........C..............................T.T...C....--------------------------------------------------------------------------------------------- 152 |
| PRCV-GXNN-X83-2022-PQ204794 .......TT........C........................ .....T.T...C....--------------------------------------------------------------------------------------------- 152 |
| PRCV-GXNN-X103-2022-PQ204795 .......TT........C..............................T.T...C....--------------------------------------------------------------------------------------------- 152 |
| PRCV-GXNN-X168-2022-PQ204796 .......TT........C..............................T.T...C....--------------------------------------------------------------------------------------------- 152 |
| PRCV-GXNN-X227-2022-PQ204797 .......TT........C..............................T.T...C....--------------------------------------------------------------------------------------------- 152 |
| PRCV-GXNN-X232-2022-PQ204798 .......TT........C..............................T.T...C....--------------------------------------------------------------------------------------------- 152 |
| PRCV-GXNN-X238-2024-PQ204810 ........T........C..............................T.T...C....--------------------------------------------------------------------------------------------- 152 |
| PRCV-GXNN-X259-2022-PQ204799 .......TT........C..............................T.T...C....--------------------------------------------------------------------------------------------- 152 |
| PRCV-90-DK-OK078898.1-Denmark ..................G........................................--------------------------------------------------------------------------------------------- 152 |
| PRCV-91V44-OR689864.1-Belgium ..................G.................................GT.....TTG------------------------------------------------------------------------------------------ 152 |
| PRCV-135isolate-86135308-OM830318.1-UK ..................G........................................--------------------------------------------------------------------------------------------- 152 |
| PRCV-310isolate-AR310-OM830319.1-USA .......C..........G.T......A......................T.GT..A..--------------------------------------------------------------------------------------------- 152 |
| PRCV-1894X-OR209253.1-USA .......C..........G.T......A.................AA.A.TGG--------------------------------------------------------------------------------------------------- 152 |
| PRCV-1508712-III-NPTV-Parma-OR689863.1-Italy..................G..................................T.....--------------------------------------------------------------------------------------------- 152 |
| PRCV-86137004-X60089.1-UK ..................G........................................--------------------------------------------------------------------------------------------- 152 |
| PRCV-AR310-OR209251.1-USA .......C..........G.T......A.......................----------------------------------------------------------------------------------------------------- 152 |
| PRCV-HOL87-M94097-Netherlands ..................G........................................--------------------------------------------------------------------------------------------- 152 |
| PRCV-ISU-1-DQ811787.1-USA .......C..........G.T......A......................T..T.....TTG------------------------------------------------------------------------------------------ 152 |
| PRCV-ISU-1-OM830321.1-USA .......C..........G.T......A......................T..T.....TTG------------------------------------------------------------------------------------------ 152 |
| PRCV-ISU20-92330-OR209254.1-USA .......C.CC.C.......TC.T...A......................T..T.....TTGTTCTAAATTGACTAATAGAAC--------------------------------------------------------------------- 152 |
| PRCV-KPRCV2401-PP781501.1-Korea ........................A.................................T--------------------------------------------------------------------------------------------- 152 |
| PRCV-KPRCV2402-PP781502.1-Korea ........................A.................................T--------------------------------------------------------------------------------------------- 152 |
| PRCV-KPRCV2403-PP781503.1-Korea ........................A..................................--------------------------------------------------------------------------------------------- 152 |
| PRCV-LEPP1-OR209252.1-USA .......C..........G.T......A.......................----------------------------------------------------------------------------------------------------- 152 |
| PRCV-OH7269-KR270796.1-USA .......C.C....C...G.T......A......................T.GT.....TTGTTCTAAACTGACTAATAGAAC--------------------------------------------------------------------- 152 |
| PRCV-Minnesota-46140-KY406735.1-USA .......T....C.....G.T......A.......T..............TT.T.....TTG------------------------------------------------------------------------------------------ 152 |
| PRCV-NM-PV096984.1-China .......C..........G.T......A.......................----------------------------------------------------------------------------------------------------- 152 |
| PRCV-RM4-Z24675.1-France ..................G........................................--------------------------------------------------------------------------------------------- 152 |
| TGEV-virulent-Purdue-DQ811789.2-USA .........C........G.T.....C..........................T.....TTGTTCTAAATTGACTAATAGAACTATAGGCAACCATTGGAATCTCATTGAAACCTTCCTTCTAAACTATAGTAGTAGGTTACCACCTAATTC 152 |
| TGEV-Ly23-PQ189446.1-China ..................G.T................................T.....TTGTTCTAAATTGACTAATAGAACTATAGGTAACCATTGGAATCTCATTGAAACCTTCCTTCTAAATTATAGTAGTAGGTTATCACCTAATTC 152 |
| TGEV-SouthDakota154-KX900411.1-USA .......T.C........G.T......A......................T..T.....TTGTTCTAAATTGACTAATAGAACTATAGGTAAACATTGGAATCTTATTGATACCTTTCTTATAAATTATAGTAGCAGGTTACCACCTAATTC 152 |
| TGEV-H16-FJ755618.2-China ..................G.T................................T.....TTGTTCTAAATTGACTAATAGAACTATAGGTAACCATTGGAATCTCATTGAAACCTTCCTTCTAAATTATAGTAGTAGGTTACCACCTAATTC 152 |
|  |
| Majority XXXXXXXXXXXXXXXXXXXXXXXXXXXXXXXXXXXXXXXXXXXXXXXXXXXXXXXXXXXXXXXXXXXXXXXXXXXXXXXXXXXXXXXXXXXXXXXXXXXXXXXXXXXXXXXXXXXXXXXXXXXXXXXXXXXXXXXXXXXXXXXXXXXXXXXX |
| -------+---------+---------+---------+---------+---------+---------+---------+---------+---------+---------+---------+---------+---------+---------+---- |
| 160 170 180 190 200 210 220 230 240 250 260 270 280 290 300 |
| -------+---------+---------+---------+---------+---------+---------+---------+---------+---------+---------+---------+---------+---------+---------+---- |
| PRCV-137isolate-86135308-OM830320.1-UK -------------------------------------------------------------------------------------------------------------------------------------------------------- 304 |
| PRCV-GXBS-B8-2023-PQ204803 -------------------------------------------------------------------------------------------------------------------------------------------------------- 304 |
| PRCV-GXHZ-B1-2023-PQ204802 -------------------------------------------------------------------------------------------------------------------------------------------------------- 304 |
| PRCV-GXHZ-K5-2023-PQ204804 -------------------------------------------------------------------------------------------------------------------------------------------------------- 304 |
| PRCV-GXHZ-K17-2023-PQ204805 -------------------------------------------------------------------------------------------------------------------------------------------------------- 304 |
| PRCV-GXLB-M66-2023-PQ204806 -------------------------------------------------------------------------------------------------------------------------------------------------------- 304 |
| PRCV-GXLB-M67-2023-PQ204807 -------------------------------------------------------------------------------------------------------------------------------------------------------- 304 |
| PRCV-GXLB-M68-2023-PQ204808 -------------------------------------------------------------------------------------------------------------------------------------------------------- 304 |
| PRCV-GXNN-W2-2023-PQ204800 -------------------------------------------------------------------------------------------------------------------------------------------------------- 304 |
| PRCV-GXNN-W4-2023-PQ204801 -------------------------------------------------------------------------------------------------------------------------------------------------------- 304 |
| PRCV-GXNN-X35-2024-PQ204809 -------------------------------------------------------------------------------------------------------------------------------------------------------- 304 |
| PRCV-GXNN-X83-2022-PQ204794 -------------------------------------------------------------------------------------------------------------------------------------------------------- 304 |
| PRCV-GXNN-X103-2022-PQ204795 -------------------------------------------------------------------------------------------------------------------------------------------------------- 304 |
| PRCV-GXNN-X168-2022-PQ204796 -------------------------------------------------------------------------------------------------------------------------------------------------------- 304 |
| PRCV-GXNN-X227-2022-PQ204797 -------------------------------------------------------------------------------------------------------------------------------------------------------- 304 |
| PRCV-GXNN-X232-2022-PQ204798 -------------------------------------------------------------------------------------------------------------------------------------------------------- 304 |
| PRCV-GXNN-X238-2024-PQ204810 -------------------------------------------------------------------------------------------------------------------------------------------------------- 304 |
| PRCV-GXNN-X259-2022-PQ204799 -------------------------------------------------------------------------------------------------------------------------------------------------------- 304 |
| PRCV-90-DK-OK078898.1-Denmark -------------------------------------------------------------------------------------------------------------------------------------------------------- 304 |
| PRCV-91V44-OR689864.1-Belgium -------------------------------------------------------------------------------------------------------------------------------------------------------- 304 |
| PRCV-135isolate-86135308-OM830318.1-UK -------------------------------------------------------------------------------------------------------------------------------------------------------- 304 |
| PRCV-310isolate-AR310-OM830319.1-USA -------------------------------------------------------------------------------------------------------------------------------------------------------- 304 |
| PRCV-1894X-OR209253.1-USA -------------------------------------------------------------------------------------------------------------------------------------------------------- 304 |
| PRCV-1508712-III-NPTV-Parma-OR689863.1-Italy-------------------------------------------------------------------------------------------------------------------------------------------------------- 304 |
| PRCV-86137004-X60089.1-UK -------------------------------------------------------------------------------------------------------------------------------------------------------- 304 |
| PRCV-AR310-OR209251.1-USA -------------------------------------------------------------------------------------------------------------------------------------------------------- 304 |
| PRCV-HOL87-M94097-Netherlands -------------------------------------------------------------------------------------------------------------------------------------------------------- 304 |
| PRCV-ISU-1-DQ811787.1-USA -------------------------------------------------------------------------------------------------------------------------------------------------------- 304 |
| PRCV-ISU-1-OM830321.1-USA -------------------------------------------------------------------------------------------------------------------------------------------------------- 304 |
| PRCV-ISU20-92330-OR209254.1-USA -------------------------------------------------------------------------------------------------------------------------------------------------------- 304 |
| PRCV-KPRCV2401-PP781501.1-Korea -------------------------------------------------------------------------------------------------------------------------------------------------------- 304 |
| PRCV-KPRCV2402-PP781502.1-Korea -------------------------------------------------------------------------------------------------------------------------------------------------------- 304 |
| PRCV-KPRCV2403-PP781503.1-Korea -------------------------------------------------------------------------------------------------------------------------------------------------------- 304 |
| PRCV-LEPP1-OR209252.1-USA -------------------------------------------------------------------------------------------------------------------------------------------------------- 304 |
| PRCV-OH7269-KR270796.1-USA -------------------------------------------------------------------------------------------------------------------------------------------------------- 304 |
| PRCV-Minnesota-46140-KY406735.1-USA -------------------------------------------------------------------------------------------------------------------------------------------------------- 304 |
| PRCV-NM-PV096984.1-China -------------------------------------------------------------------------------------------------------------------------------------------------------- 304 |
| PRCV-RM4-Z24675.1-France -------------------------------------------------------------------------------------------------------------------------------------------------------- 304 |
| TGEV-virulent-Purdue-DQ811789.2-USA AGATGTGGTGTTAGGTGATTATTTTCCTACTGTACAACCTTGGTTTAATTGCATTCGCAATGATAGTAATGACCTTTATGTTACACTGGAAAATCTTAAAGCATTGTATTGGGATTATGCTACAGAAAATATCACTTGGAATCACAGACAAC 304 |
| TGEV-Ly23-PQ189446.1-China AGATGCGGTGTTAGGTGATTATTTTCCTACTGTACAACCTTGGTTTAATTGCATTCGCAATAATAGTAATGACCTTTATGTTACATTGGAAAATCTTAAAGCATTGTATTGGGATTATGCTATAGAAAATATCACTTCGAATCACAAACAAC 304 |
| TGEV-SouthDakota154-KX900411.1-USA AGATGTGGTGTTAGGTGATTATTTTCCTACTGTACAACCTTGGTTTAATTGTATTCGCAATGATAGTAATGACCTTTATGTTACATTGGAAAATCTTAAAGCATTGTATTGGGATTATGCTACAGAAAATATCACTGCGAATCACAGACAAC 304 |
| TGEV-H16-FJ755618.2-China AGATGCGGTGTTAGGTGATTATTTTCCTACTGTACAACCTTGGTTTAATTGCATTCGCAATAATAGTAATGACCTTTATGTTACATTGGAAAATCTTAAAGCATTGTATTGGGATTATGCTATAGAAAATATCACTTCGAATCACAAACAAC 304 |
|  |
| Majority XXXXXXXXXXXXXXXXXXXXXXXXXXXXXXXXXXXXXXXXXXXXXXXXXXXXXXXXXXXXXXXXXXXXXXXXXXXXXXXXXXXXXXXXXXXXXXXXXXXXXXXXXXXXXXXXXXXXXXXXXXXXXXXXXXXXXXXXXXXXXXXXXXXXXXXX |
| -----+---------+---------+---------+---------+---------+---------+---------+---------+---------+---------+---------+---------+---------+---------+------ |
| 310 320 330 340 350 360 370 380 390 400 410 420 430 440 450 |
| -----+---------+---------+---------+---------+---------+---------+---------+---------+---------+---------+---------+---------+---------+---------+------ |
| PRCV-137isolate-86135308-OM830320.1-UK -------------------------------------------------------------------------------------------------------------------------------------------------------- 456 |
| PRCV-GXBS-B8-2023-PQ204803 -------------------------------------------------------------------------------------------------------------------------------------------------------- 456 |
| PRCV-GXHZ-B1-2023-PQ204802 -------------------------------------------------------------------------------------------------------------------------------------------------------- 456 |
| PRCV-GXHZ-K5-2023-PQ204804 -------------------------------------------------------------------------------------------------------------------------------------------------------- 456 |
| PRCV-GXHZ-K17-2023-PQ204805 -------------------------------------------------------------------------------------------------------------------------------------------------------- 456 |
| PRCV-GXLB-M66-2023-PQ204806 -------------------------------------------------------------------------------------------------------------------------------------------------------- 456 |
| PRCV-GXLB-M67-2023-PQ204807 -------------------------------------------------------------------------------------------------------------------------------------------------------- 456 |
| PRCV-GXLB-M68-2023-PQ204808 -------------------------------------------------------------------------------------------------------------------------------------------------------- 456 |
| PRCV-GXNN-W2-2023-PQ204800 -------------------------------------------------------------------------------------------------------------------------------------------------------- 456 |
| PRCV-GXNN-W4-2023-PQ204801 -------------------------------------------------------------------------------------------------------------------------------------------------------- 456 |
| PRCV-GXNN-X35-2024-PQ204809 -------------------------------------------------------------------------------------------------------------------------------------------------------- 456 |
| PRCV-GXNN-X83-2022-PQ204794 -------------------------------------------------------------------------------------------------------------------------------------------------------- 456 |
| PRCV-GXNN-X103-2022-PQ204795 -------------------------------------------------------------------------------------------------------------------------------------------------------- 456 |
| PRCV-GXNN-X168-2022-PQ204796 -------------------------------------------------------------------------------------------------------------------------------------------------------- 456 |
| PRCV-GXNN-X227-2022-PQ204797 -------------------------------------------------------------------------------------------------------------------------------------------------------- 456 |
| PRCV-GXNN-X232-2022-PQ204798 -------------------------------------------------------------------------------------------------------------------------------------------------------- 456 |
| PRCV-GXNN-X238-2024-PQ204810 -------------------------------------------------------------------------------------------------------------------------------------------------------- 456 |
| PRCV-GXNN-X259-2022-PQ204799 -------------------------------------------------------------------------------------------------------------------------------------------------------- 456 |
| PRCV-90-DK-OK078898.1-Denmark -------------------------------------------------------------------------------------------------------------------------------------------------------- 456 |
| PRCV-91V44-OR689864.1-Belgium -------------------------------------------------------------------------------------------------------------------------------------------------------- 456 |
| PRCV-135isolate-86135308-OM830318.1-UK -------------------------------------------------------------------------------------------------------------------------------------------------------- 456 |
| PRCV-310isolate-AR310-OM830319.1-USA -------------------------------------------------------------------------------------------------------------------------------------------------------- 456 |
| PRCV-1894X-OR209253.1-USA -------------------------------------------------------------------------------------------------------------------------------------------------------- 456 |
| PRCV-1508712-III-NPTV-Parma-OR689863.1-Italy-------------------------------------------------------------------------------------------------------------------------------------------------------- 456 |
| PRCV-86137004-X60089.1-UK -------------------------------------------------------------------------------------------------------------------------------------------------------- 456 |
| PRCV-AR310-OR209251.1-USA -------------------------------------------------------------------------------------------------------------------------------------------------------- 456 |
| PRCV-HOL87-M94097-Netherlands -------------------------------------------------------------------------------------------------------------------------------------------------------- 456 |
| PRCV-ISU-1-DQ811787.1-USA -------------------------------------------------------------------------------------------------------------------------------------------------------- 456 |
| PRCV-ISU-1-OM830321.1-USA -------------------------------------------------------------------------------------------------------------------------------------------------------- 456 |
| PRCV-ISU20-92330-OR209254.1-USA -------------------------------------------------------------------------------------------------------------------------------------------------------- 456 |
| PRCV-KPRCV2401-PP781501.1-Korea -------------------------------------------------------------------------------------------------------------------------------------------------------- 456 |
| PRCV-KPRCV2402-PP781502.1-Korea -------------------------------------------------------------------------------------------------------------------------------------------------------- 456 |
| PRCV-KPRCV2403-PP781503.1-Korea -------------------------------------------------------------------------------------------------------------------------------------------------------- 456 |
| PRCV-LEPP1-OR209252.1-USA -------------------------------------------------------------------------------------------------------------------------------------------------------- 456 |
| PRCV-OH7269-KR270796.1-USA -------------------------------------------------------------------------------------------------------------------------------------------------------- 456 |
| PRCV-Minnesota-46140-KY406735.1-USA -------------------------------------------------------------------------------------------------------------------------------------------------------- 456 |
| PRCV-NM-PV096984.1-China -------------------------------------------------------------------------------------------------------------------------------------------------------- 456 |
| PRCV-RM4-Z24675.1-France -------------------------------------------------------------------------------------------------------------------------------------------------------- 456 |
| TGEV-virulent-Purdue-DQ811789.2-USA GGTTAAACGTAGTCGTTAATGGATACCCATACTCCATCACAGTTACAACAACCCGCAATTTTAATTCTGCTGAAGGTGCTATTATATGCATTTGTAAGGGCTCACCACCTACTACCACCACAGAATCTAGTTTGACTTGCAATTGGGGTAGT 456 |
| TGEV-Ly23-PQ189446.1-China GGTTAAACGTAGTCGTTAATGGATACCCATACTCCATCACAGTTACAACAACCCGCAATTTTAATTCTGCTGAAGGTGCTATTATATGCATTTGCAAGGGCTCACCACCTACTACCACCACAGAATCTAGTTTGACTTGCAATTGGGGTAGT 456 |
| TGEV-SouthDakota154-KX900411.1-USA GGTTAAACGTAGTCGTTAATGGATACCCATACTCCATCACAGTTACAACAACCCGCAATTTTAATTCTGCTGAAGGTGCTATTATATGCATTTGTAAGGGTTCACCACCTACTACCACCACCGAGTCTAGTCTGACTTGCAATTGGGGTAGT 456 |
| TGEV-H16-FJ755618.2-China GGTTAAACGTAGTCGTTAATGGATACCCATACTCCATCACAGTTACAACAACCCGCAATTTTAATTCTGCTGAAGGTGCTATTATATGCATTTGCAAGGGCTCACCACCTACTACCACCACAGAATCTAGTTTGACTTGCAATTGGGGTAGT 456 |
|  |
| Majority XXXXXXXXXXXXXXXXXXXXXXXXXXXXXXXXXXXXXXXXXXXXXXXXXXXXXXXXXXXXXXXXXXXXXXXXXXXXXXXXXXXXXXXXXXXXXXXXXXXXXXXXXXXXXXXXXXXXXXXXXXXXXXXXXXXXXXXXXXXXXXXXXXXXXXXX |
| ---+---------+---------+---------+---------+---------+---------+---------+---------+---------+---------+---------+---------+---------+---------+-------- |
| 460 470 480 490 500 510 520 530 540 550 560 570 580 590 600 |
| ---+---------+---------+---------+---------+---------+---------+---------+---------+---------+---------+---------+---------+---------+---------+-------- |
| PRCV-137isolate-86135308-OM830320.1-UK -------------------------------------------------------------------------------------------------------------------------------------------------------- 608 |
| PRCV-GXBS-B8-2023-PQ204803 -------------------------------------------------------------------------------------------------------------------------------------------------------- 608 |
| PRCV-GXHZ-B1-2023-PQ204802 -------------------------------------------------------------------------------------------------------------------------------------------------------- 608 |
| PRCV-GXHZ-K5-2023-PQ204804 -------------------------------------------------------------------------------------------------------------------------------------------------------- 608 |
| PRCV-GXHZ-K17-2023-PQ204805 -------------------------------------------------------------------------------------------------------------------------------------------------------- 608 |
| PRCV-GXLB-M66-2023-PQ204806 -------------------------------------------------------------------------------------------------------------------------------------------------------- 608 |
| PRCV-GXLB-M67-2023-PQ204807 -------------------------------------------------------------------------------------------------------------------------------------------------------- 608 |
| PRCV-GXLB-M68-2023-PQ204808 -------------------------------------------------------------------------------------------------------------------------------------------------------- 608 |
| PRCV-GXNN-W2-2023-PQ204800 -------------------------------------------------------------------------------------------------------------------------------------------------------- 608 |
| PRCV-GXNN-W4-2023-PQ204801 -------------------------------------------------------------------------------------------------------------------------------------------------------- 608 |
| PRCV-GXNN-X35-2024-PQ204809 -------------------------------------------------------------------------------------------------------------------------------------------------------- 608 |
| PRCV-GXNN-X83-2022-PQ204794 -------------------------------------------------------------------------------------------------------------------------------------------------------- 608 |
| PRCV-GXNN-X103-2022-PQ204795 -------------------------------------------------------------------------------------------------------------------------------------------------------- 608 |
| PRCV-GXNN-X168-2022-PQ204796 -------------------------------------------------------------------------------------------------------------------------------------------------------- 608 |
| PRCV-GXNN-X227-2022-PQ204797 -------------------------------------------------------------------------------------------------------------------------------------------------------- 608 |
| PRCV-GXNN-X232-2022-PQ204798 -------------------------------------------------------------------------------------------------------------------------------------------------------- 608 |
| PRCV-GXNN-X238-2024-PQ204810 -------------------------------------------------------------------------------------------------------------------------------------------------------- 608 |
| PRCV-GXNN-X259-2022-PQ204799 -------------------------------------------------------------------------------------------------------------------------------------------------------- 608 |
| PRCV-90-DK-OK078898.1-Denmark -------------------------------------------------------------------------------------------------------------------------------------------------------- 608 |
| PRCV-91V44-OR689864.1-Belgium -------------------------------------------------------------------------------------------------------------------------------------------------------- 608 |
| PRCV-135isolate-86135308-OM830318.1-UK -------------------------------------------------------------------------------------------------------------------------------------------------------- 608 |
| PRCV-310isolate-AR310-OM830319.1-USA -------------------------------------------------------------------------------------------------------------------------------------------------------- 608 |
| PRCV-1894X-OR209253.1-USA -------------------------------------------------------------------------------------------------------------------------------------------------------- 608 |
| PRCV-1508712-III-NPTV-Parma-OR689863.1-Italy-------------------------------------------------------------------------------------------------------------------------------------------------------- 608 |
| PRCV-86137004-X60089.1-UK -------------------------------------------------------------------------------------------------------------------------------------------------------- 608 |
| PRCV-AR310-OR209251.1-USA -------------------------------------------------------------------------------------------------------------------------------------------------------- 608 |
| PRCV-HOL87-M94097-Netherlands -------------------------------------------------------------------------------------------------------------------------------------------------------- 608 |
| PRCV-ISU-1-DQ811787.1-USA -------------------------------------------------------------------------------------------------------------------------------------------------------- 608 |
| PRCV-ISU-1-OM830321.1-USA -------------------------------------------------------------------------------------------------------------------------------------------------------- 608 |
| PRCV-ISU20-92330-OR209254.1-USA -------------------------------------------------------------------------------------------------------------------------------------------------------- 608 |
| PRCV-KPRCV2401-PP781501.1-Korea -------------------------------------------------------------------------------------------------------------------------------------------------------- 608 |
| PRCV-KPRCV2402-PP781502.1-Korea -------------------------------------------------------------------------------------------------------------------------------------------------------- 608 |
| PRCV-KPRCV2403-PP781503.1-Korea -------------------------------------------------------------------------------------------------------------------------------------------------------- 608 |
| PRCV-LEPP1-OR209252.1-USA -------------------------------------------------------------------------------------------------------------------------------------------------------- 608 |
| PRCV-OH7269-KR270796.1-USA -------------------------------------------------------------------------------------------------------------------------------------------------------- 608 |
| PRCV-Minnesota-46140-KY406735.1-USA -------------------------------------------------------------------------------------------------------------------------------------------------------- 608 |
| PRCV-NM-PV096984.1-China -------------------------------------------------------------------------------------------------------------------------------------------------------- 608 |
| PRCV-RM4-Z24675.1-France -------------------------------------------------------------------------------------------------------------------------------------------------------- 608 |
| TGEV-virulent-Purdue-DQ811789.2-USA GAGTGCAGGTTAAACCATAAGTTCCCTATATGTCCTTCTAATTCAGAGGCAAATTGTGGTAATATGCTGTATGGCCTACAATGGTTTGCAGATGAGGTTGTTGCTTATTTACATGGTGCTAGTTACCGTATTAGTTTTGAAAATCAATGGTC 608 |
| TGEV-Ly23-PQ189446.1-China GAGTGCAGGTTAAACCATAAGTTCCCTATATGTCCTTCTAATTCAGAGGCAAATTGTGGTAATATGCTGTATGGCCTACAATGGTTTGCAGATGCGGTTGTTGCTTATTTACATGGTGCTAGTTACCGTATTAGTTTTGAAAATCAATGGTC 608 |
| TGEV-SouthDakota154-KX900411.1-USA GAGTGCAGGTTGAACCATAAGTTCCCTATATGTCCTTCTAATTCTGAGGCAAATTGTGGTAGTATGTTATATGGCTTACAATGGTCTGCAGATGCGGTTGTTGCTTATTTACATGGTGCCAGTTACCGTATTAGTTTTGAAAATCAATGGTC 608 |
| TGEV-H16-FJ755618.2-China GAGTGCAGGTTAAACCATAAGTTCCCTATATGTCCTTCTAATTCAGAGGCAAATTGTGGTAATATGCTGTATGGCCTACAATGGTTTGCAGATGCGGTTGTTGCTTATTTACATGGTGCTAGTTACCGTATTAGTTTTGAAAATCAATGGTC 608 |
|  |
| Majority XXXXXXXXXXXXXXXXXXXXXXXXXXXXXXXXXXXXXXXXXXXXXXXXXXXXXXXXXXXXXXXXXXXXXXXXXXXXXXXXXXXXXXXXXXXXXXXXXXXXXXXXXXXXXXXXXXXXXXXXXXXTACTTCTGTAGTTTCCAATTGCAXXXXXX |
| -+---------+---------+---------+---------+---------+---------+---------+---------+---------+---------+---------+---------+---------+---------+---------+ |
| 610 620 630 640 650 660 670 680 690 700 710 720 730 740 750 760 |
| -+---------+---------+---------+---------+---------+---------+---------+---------+---------+---------+---------+---------+---------+---------+---------+ |
| PRCV-137isolate-86135308-OM830320.1-UK ---------------------------------------------------------------------------------------------------------------------------......C................------ 760 |
| PRCV-GXBS-B8-2023-PQ204803 ---------------------------------------------------------------------------------------------------------------------------.......................------ 760 |
| PRCV-GXHZ-B1-2023-PQ204802 ---------------------------------------------------------------------------------------------------------------------------.......................------ 760 |
| PRCV-GXHZ-K5-2023-PQ204804 ---------------------------------------------------------------------------------------------------------------------------.......................------ 760 |
| PRCV-GXHZ-K17-2023-PQ204805 ---------------------------------------------------------------------------------------------------------------------------.......................------ 760 |
| PRCV-GXLB-M66-2023-PQ204806 ---------------------------------------------------------------------------------------------------------------------------.......................------ 760 |
| PRCV-GXLB-M67-2023-PQ204807 ---------------------------------------------------------------------------------------------------------------------------.......................------ 760 |
| PRCV-GXLB-M68-2023-PQ204808 ---------------------------------------------------------------------------------------------------------------------------.......................------ 760 |
| PRCV-GXNN-W2-2023-PQ204800 ---------------------------------------------------------------------------------------------------------------------------.......................------ 760 |
| PRCV-GXNN-W4-2023-PQ204801 ---------------------------------------------------------------------------------------------------------------------------.......................------ 760 |
| PRCV-GXNN-X35-2024-PQ204809 ---------------------------------------------------------------------------------------------------------------------------.......................------ 760 |
| PRCV-GXNN-X83-2022-PQ204794 ---------------------------------------------------------------------------------------------------------------------------...............T.......------ 760 |
| PRCV-GXNN-X103-2022-PQ204795 ---------------------------------------------------------------------------------------------------------------------------...............T.......------ 760 |
| PRCV-GXNN-X168-2022-PQ204796 ---------------------------------------------------------------------------------------------------------------------------...............T.......------ 760 |
| PRCV-GXNN-X227-2022-PQ204797 ---------------------------------------------------------------------------------------------------------------------------...............T.......------ 760 |
| PRCV-GXNN-X232-2022-PQ204798 ---------------------------------------------------------------------------------------------------------------------------...............T.......------ 760 |
| PRCV-GXNN-X238-2024-PQ204810 ---------------------------------------------------------------------------------------------------------------------------.......................------ 760 |
| PRCV-GXNN-X259-2022-PQ204799 ---------------------------------------------------------------------------------------------------------------------------...............T.......------ 760 |
| PRCV-90-DK-OK078898.1-Denmark ---------------------------------------------------------------------------------------------------------------------------......C................------ 760 |
| PRCV-91V44-OR689864.1-Belgium ---------------------------------------------------------------------------------------------------------------------------------------...........------ 760 |
| PRCV-135isolate-86135308-OM830318.1-UK ---------------------------------------------------------------------------------------------------------------------------......C................------ 760 |
| PRCV-310isolate-AR310-OM830319.1-USA ------------------------------------------------------------------------------------------------------------------------------------GT............------ 760 |
| PRCV-1894X-OR209253.1-USA ---------------------------------------------------------------------------------------------------------------------------....A.C................------ 760 |
| PRCV-1508712-III-NPTV-Parma-OR689863.1-Italy---------------------------------------------------------------------------------------------------------------------------.......................ACGTCA 760 |
| PRCV-86137004-X60089.1-UK ---------------------------------------------------------------------------------------------------------------------------......C................------ 760 |
| PRCV-AR310-OR209251.1-USA ----------------------------------------------------------------CTTTGGTGGTTTAATCCTGTTTATGATGTCAGTTATTATAGAGTTAATAATAAAAATGG....A.C................------ 760 |
| PRCV-HOL87-M94097-Netherlands ---------------------------------------------------------------------------------------------------------------------------......C................------ 760 |
| PRCV-ISU-1-DQ811787.1-USA ---------------------------------------------------------------------------------------------------------------------------------------...........------ 760 |
| PRCV-ISU-1-OM830321.1-USA ---------------------------------------------------------------------------------------------------------------------------------------...........------ 760 |
| PRCV-ISU20-92330-OR209254.1-USA ---------------------------------------------------------------------------------------------------------------------------..TAGG.AA.CA..GG...CT..------ 760 |
| PRCV-KPRCV2401-PP781501.1-Korea ---------------------------------------------------------------------------------------------------------------------------.......................------ 760 |
| PRCV-KPRCV2402-PP781502.1-Korea ---------------------------------------------------------------------------------------------------------------------------.......................------ 760 |
| PRCV-KPRCV2403-PP781503.1-Korea ---------------------------------------------------------------------------------------------------------------------------.......................------ 760 |
| PRCV-LEPP1-OR209252.1-USA ----------------------------------------------------------------CTTTGGTGGTTTAATCCTGTTTATGATGTCAGTTATTATAGAGTTAATAATAAAAATGG....A.C................------ 760 |
| PRCV-OH7269-KR270796.1-USA ---------------------------------------------------------------------------------------------------------------------------..TAGG.AA.CA..GG...CT..------ 760 |
| PRCV-Minnesota-46140-KY406735.1-USA ---------------------------------------------------------------------------------------------------------------------------------------...........------ 760 |
| PRCV-NM-PV096984.1-China ----------------------------------------------------------------CTTTGGTGGTTTAATCCTGTTTATGATGTCAGTTATTATAGAGTTAATAATAAAAATGG....A.C................------ 760 |
| PRCV-RM4-Z24675.1-France ---------------------------------------------------------------------------------------------------------------------------......C................------ 760 |
| TGEV-virulent-Purdue-DQ811789.2-USA TGGCACTGTCACACTTGGTGATATGCGTGCGACAACATTAGAAGTCGCTGGCACGCTTGTAGACCTTTGGTGGTTTAATCCTGTTTATGATGTCAGTTATTATAGAGTTAATAATAAAAATGG....A.C................------ 760 |
| TGEV-Ly23-PQ189446.1-China TGGCACTGTTACACTTGGTGATATGCGTGCGACTACATTAGAAACCGCTGGCACGCTTGTAGACCTTTGGTGGTTTAATCCTGTTTATGATGTCAGTTATTATAGAGTTAATAATAAAAATGG....A.C................------ 760 |
| TGEV-SouthDakota154-KX900411.1-USA TGGCACTGTTACACTTGGTGATATGCGTGCGACTACGTCGCAAACCGCTGGCACGCTTGTAGACCTTTGGTGGTTTAATCCTGTTTATGATGTCAGTTATTATAGAGTTAATAACAAAAATGG....A..................------ 760 |
| TGEV-H16-FJ755618.2-China TGGCACTGTTACACTTGGTGATATGCGTGCGACTACATTAGAAACCGCTGGCACGCTTGTAGACCTTTGGTGGTTTAATCCTGTTTATGATGTCAGTTATTATAGAGTTAATAATAAAAATGG....A.C................------ 760 |
|  |
| Majority XXXCTGATCAATGTGCTAGTTATGTGGCTAATGTTTTTACTACACAGCCAGGAGGCTTTATACCATCAGATTTTAGTTTTAATAATTGGTTCCTCCTAACTAATAGCTCCACGTTGGTTAGTGGTAAATTAGTTACCAAACAGCCTCTATTA |
| ---------+---------+---------+---------+---------+---------+---------+---------+---------+---------+---------+---------+---------+---------+---------+-- |
| 770 780 790 800 810 820 830 840 850 860 870 880 890 900 910 |
| ---------+---------+---------+---------+---------+---------+---------+---------+---------+---------+---------+---------+---------+---------+---------+-- |
| PRCV-137isolate-86135308-OM830320.1-UK ---.........................................................................................A...............................C........................... 912 |
| PRCV-GXBS-B8-2023-PQ204803 ---...........................................A............................................T...T................T....................................... 912 |
| PRCV-GXHZ-B1-2023-PQ204802 ---...........................................A............................................T...T................T....................................... 912 |
| PRCV-GXHZ-K5-2023-PQ204804 ---......G....................................A............................................T...T................T....................................... 912 |
| PRCV-GXHZ-K17-2023-PQ204805 ---...........................................A............................................T...T................T....................................... 912 |
| PRCV-GXLB-M66-2023-PQ204806 ---.......................A...................A................................................T..........T.....T...................................T... 912 |
| PRCV-GXLB-M67-2023-PQ204807 ---.......................A...................A.........C......................................T..........T.....T...................................T... 912 |
| PRCV-GXLB-M68-2023-PQ204808 ---.......................A...................A................................................T..........T.....T...................................T... 912 |
| PRCV-GXNN-W2-2023-PQ204800 ---.................................C.........A................................................T..........T.....T...................................T... 912 |
| PRCV-GXNN-W4-2023-PQ204801 ---...........................................A................................................T..........T.....T...................................T... 912 |
| PRCV-GXNN-X35-2024-PQ204809 ---........................................G..A............................................T...T................T....................................... 912 |
| PRCV-GXNN-X83-2022-PQ204794 ---...........................................A.....G.........................................TT................T....................C.................. 912 |
| PRCV-GXNN-X103-2022-PQ204795 ---...........................................A.....G.........................................TT................T....................C.................. 912 |
| PRCV-GXNN-X168-2022-PQ204796 ---...........................................A.....G.........................................TT................T....................C.................. 912 |
| PRCV-GXNN-X227-2022-PQ204797 ---...........................................A.....G.........................................TT................T....................C.................. 912 |
| PRCV-GXNN-X232-2022-PQ204798 ---...........................................A.....G.........................................TT.......C........T....................C.................. 912 |
| PRCV-GXNN-X238-2024-PQ204810 ---...........................................A............................................T...T................T....................................... 912 |
| PRCV-GXNN-X259-2022-PQ204799 ---...........................................A.....G..........................................T................T....................C.................. 912 |
| PRCV-90-DK-OK078898.1-Denmark ---..................................................................................................................................................... 912 |
| PRCV-91V44-OR689864.1-Belgium ---....................................................T................................................................................A............... 912 |
| PRCV-135isolate-86135308-OM830318.1-UK ---..................................................................................................................................................... 912 |
| PRCV-310isolate-AR310-OM830319.1-USA ---...............................C...GT......................................................T..................................................AT..... 912 |
| PRCV-1894X-OR209253.1-USA ---...............................C...........................................................TT........................................A........AT..... 912 |
| PRCV-1508712-III-NPTV-Parma-OR689863.1-ItalyCAT.C....................AA.C..........................T.....................................................T............................C............. 912 |
| PRCV-86137004-X60089.1-UK ---.........................................................................................A...............................C........................... 912 |
| PRCV-AR310-OR209251.1-USA ---...............................C...GT......................................................T..................................................AT..... 912 |
| PRCV-HOL87-M94097-Netherlands ---..................................................................................................................................................... 912 |
| PRCV-ISU-1-DQ811787.1-USA ---...............................C...GT......................................................T..................................................AT..... 912 |
| PRCV-ISU-1-OM830321.1-USA ---...............................C...GT......................................................T..................................................AT..... 912 |
| PRCV-ISU20-92330-OR209254.1-USA ---.......G....................C......GT...............T......................................T.................T................................AT..... 912 |
| PRCV-KPRCV2401-PP781501.1-Korea ---.C..................T...............................T..................................................T..T..T..................C......C............. 912 |
| PRCV-KPRCV2402-PP781502.1-Korea ---.C..................T...............................T..................................................T..T..T..................C......C............. 912 |
| PRCV-KPRCV2403-PP781503.1-Korea ---.C..................................................T..C.........A.....................................T..T.....................C......C............. 912 |
| PRCV-LEPP1-OR209252.1-USA ---...............................C...GT......................................................T..................................................AT..... 912 |
| PRCV-OH7269-KR270796.1-USA ---.......G....................C......GT...............T......................................T.................A................................AT..... 912 |
| PRCV-Minnesota-46140-KY406735.1-USA ---.........T............A............GT......A....................GA......................TT.T...............................G............C.....AT..... 912 |
| PRCV-NM-PV096984.1-China ---.C.............................C...GT...............T.........................................................................................AT..... 912 |
| PRCV-RM4-Z24675.1-France ---.......................................T..TA.........................................................................A............................... 912 |
| TGEV-virulent-Purdue-DQ811789.2-USA ---....................................................T......................................T..................................................GT..... 912 |
| TGEV-Ly23-PQ189446.1-China ---...........................................................................................T..................................................GT..... 912 |
| TGEV-SouthDakota154-KX900411.1-USA ---.......G...........................GT......................................................T...........T......................................AT....G 912 |
| TGEV-H16-FJ755618.2-China ---...........................................................................................T..................................................GT..... 912 |
|  |
| Majority GTTAATTGCTTATGGCCAGTCCCTAGCTTTGAAGAAGCAGCTTCTACATTTTGTTTTGAAGGTGCTGACTTTGATCAATGTAATGGTGCTGTTTTAAATAACACTGTAGATGTCATTAGGTTTAACCTTAATTTTACTACAAATGTACAATC |
| -------+---------+---------+---------+---------+---------+---------+---------+---------+---------+---------+---------+---------+---------+---------+---- |
| 920 930 940 950 960 970 980 990 1000 1010 1020 1030 1040 1050 1060 |
| -------+---------+---------+---------+---------+---------+---------+---------+---------+---------+---------+---------+---------+---------+---------+---- |
| PRCV-137isolate-86135308-OM830320.1-UK ..............................................................................................................C......................................... 1064 |
| PRCV-GXBS-B8-2023-PQ204803 ...............................................G....................T................................T.....C.....T...........T.......................... 1064 |
| PRCV-GXHZ-B1-2023-PQ204802 ...............................................G....................T................................T.....C.....T...........T.......................... 1064 |
| PRCV-GXHZ-K5-2023-PQ204804 ...............................T.......T.......G....................T................................T.....C.....T...........T.......................... 1064 |
| PRCV-GXHZ-K17-2023-PQ204805 ....................................................................T................................T.....C.....T...........T.......................... 1064 |
| PRCV-GXLB-M66-2023-PQ204806 ...................................G................................T......................................C.....T...........T.......................... 1064 |
| PRCV-GXLB-M67-2023-PQ204807 ...................................G................................T......................................C.....T...........T.......................... 1064 |
| PRCV-GXLB-M68-2023-PQ204808 ...................................G................................T......................................C.....T...........T.......................... 1064 |
| PRCV-GXNN-W2-2023-PQ204800 ...................................G................................T...............................G......C.....T...........T.......................... 1064 |
| PRCV-GXNN-W4-2023-PQ204801 ...................................G................................T.......................C..............C.....T...........T.......................... 1064 |
| PRCV-GXNN-X35-2024-PQ204809 ...............................................G....................T................................T.....C.....T...........T.......................... 1064 |
| PRCV-GXNN-X83-2022-PQ204794 ....................................................................T......................................C.....T...................................... 1064 |
| PRCV-GXNN-X103-2022-PQ204795 ....................................................................T......................................C.....T...................................... 1064 |
| PRCV-GXNN-X168-2022-PQ204796 ....................................................................T......................................C.....T...................................... 1064 |
| PRCV-GXNN-X227-2022-PQ204797 ....................................................................T......................................C.....T...................................... 1064 |
| PRCV-GXNN-X232-2022-PQ204798 ....C...............................................................T......................................C.....T...................................... 1064 |
| PRCV-GXNN-X238-2024-PQ204810 ...............................................G....................T................................T.....C.....T...........T.......................... 1064 |
| PRCV-GXNN-X259-2022-PQ204799 ....................................................................T......................................C.....T...................................... 1064 |
| PRCV-90-DK-OK078898.1-Denmark ..............................................................................................................C.....C................................... 1064 |
| PRCV-91V44-OR689864.1-Belgium ..............................A...............................................................................C.....C................................... 1064 |
| PRCV-135isolate-86135308-OM830318.1-UK ..............................................................................................................C......................................... 1064 |
| PRCV-310isolate-AR310-OM830319.1-USA ............................................................................................C........T..............C................................... 1064 |
| PRCV-1894X-OR209253.1-USA ............................................................................................C........T........C.....C..............................A.... 1064 |
| PRCV-1508712-III-NPTV-Parma-OR689863.1-Italy..............................A................G................T.............................................C.....C................................... 1064 |
| PRCV-86137004-X60089.1-UK ..............................................................................................................C......................................... 1064 |
| PRCV-AR310-OR209251.1-USA ............................................................................................C........T..............C................................... 1064 |
| PRCV-HOL87-M94097-Netherlands ..............................................................................................................C...........C............................. 1064 |
| PRCV-ISU-1-DQ811787.1-USA ............................................................................................C........T..............C................................... 1064 |
| PRCV-ISU-1-OM830321.1-USA ............................................................................................C........T..............C................................... 1064 |
| PRCV-ISU20-92330-OR209254.1-USA ......................................G.............................T.....C.................C......................................C.................... 1064 |
| PRCV-KPRCV2401-PP781501.1-Korea ..............................A................G..............................................................C.....C................................... 1064 |
| PRCV-KPRCV2402-PP781502.1-Korea ..............................A................G..............................................................C.....C................................... 1064 |
| PRCV-KPRCV2403-PP781503.1-Korea ..C...........................A................G..............................................................C.....C................................... 1064 |
| PRCV-LEPP1-OR209252.1-USA ............................................................................................C........T..............C................................... 1064 |
| PRCV-OH7269-KR270796.1-USA ........T.............................G...................................C..C..............C........T..............C..............C.................... 1064 |
| PRCV-Minnesota-46140-KY406735.1-USA .......................C.........A..........................................................C........T..............C.....C..........................T.. 1064 |
| PRCV-NM-PV096984.1-China ...............................................G............................................C.................C.....C................................... 1064 |
| PRCV-RM4-Z24675.1-France .....................................T........................................................................C......................................... 1064 |
| TGEV-virulent-Purdue-DQ811789.2-USA ...........................................Y...............G.......G..........................................C...........C............................. 1064 |
| TGEV-Ly23-PQ189446.1-China ...................................................................G..........................................C.....C................................... 1064 |
| TGEV-SouthDakota154-KX900411.1-USA ........T...........T.......................C..G.........................................C..C........T..............C................................... 1064 |
| TGEV-H16-FJ755618.2-China ...................................................................G..........................................C.....C................................... 1064 |
|  |
| Majority AGGTAAGGGTGCTACAGTGTTTTCATTGAACACAACGGGTGGTGTCACTCTTGAAATCTCATGTTATAATGATACAGTGAGTGATTCTAGCTTTTCCAGTTACGGTGAAATTCCGTTCGGCGTAACTAATGGACCACGGTACTGTTACGTAC |
| -----+---------+---------+---------+---------+---------+---------+---------+---------+---------+---------+---------+---------+---------+---------+------ |
| 1070 1080 1090 1100 1110 1120 1130 1140 1150 1160 1170 1180 1190 1200 1210 |
| -----+---------+---------+---------+---------+---------+---------+---------+---------+---------+---------+---------+---------+---------+---------+------ |
| PRCV-137isolate-86135308-OM830320.1-UK .......................................................................................G................................................................ 1216 |
| PRCV-GXBS-B8-2023-PQ204803 ..............................T.......................................................................T.................T.................T............. 1216 |
| PRCV-GXHZ-B1-2023-PQ204802 ..............................T.................................C.....................T...............T.................T.................T............. 1216 |
| PRCV-GXHZ-K5-2023-PQ204804 ..............................T..............................................C........................T...........T.....T.................T............. 1216 |
| PRCV-GXHZ-K17-2023-PQ204805 ..............................T.......................................................T...............T.................T.................T............. 1216 |
| PRCV-GXLB-M66-2023-PQ204806 ..............................T..........................T..............C.............................T.................T..................C............ 1216 |
| PRCV-GXLB-M67-2023-PQ204807 ..............................T..........................T..............C.............................T.................T............................... 1216 |
| PRCV-GXLB-M68-2023-PQ204808 ..............................T..........................T..............C.............................T............C....T............................... 1216 |
| PRCV-GXNN-W2-2023-PQ204800 ..............................T...........CA.............T..............C.T...............T...........T.................T....................T.......... 1216 |
| PRCV-GXNN-W4-2023-PQ204801 ..............................T...........CA.............T..............C.T...............T...........T.................T............................... 1216 |
| PRCV-GXNN-X35-2024-PQ204809 ..............................T.......................................................................T.................T.................T............. 1216 |
| PRCV-GXNN-X83-2022-PQ204794 ...........................A............................................C.................T...........T.................T.................T............. 1216 |
| PRCV-GXNN-X103-2022-PQ204795 ...........................A............................................C.................T...........T.................T.................T.....C....... 1216 |
| PRCV-GXNN-X168-2022-PQ204796 ...........................A............................................C.................T...........T.................T.................T............. 1216 |
| PRCV-GXNN-X227-2022-PQ204797 ...........................A............................................C.................T...........T.................T.................T............. 1216 |
| PRCV-GXNN-X232-2022-PQ204798 ...........................A............................................C.................T...........T.................T.................T............. 1216 |
| PRCV-GXNN-X238-2024-PQ204810 ..............................T.......................................................................T.................T.................T............. 1216 |
| PRCV-GXNN-X259-2022-PQ204799 ...........................A............................................C.................T...........T.................T.................T............. 1216 |
| PRCV-90-DK-OK078898.1-Denmark ........................T..............................................................G..T.....................................G....................... 1216 |
| PRCV-91V44-OR689864.1-Belgium .......................................................................................G..T............................................................. 1216 |
| PRCV-135isolate-86135308-OM830318.1-UK .......................................................................................G......G......................................................... 1216 |
| PRCV-310isolate-AR310-OM830319.1-USA ............C.......................................................................C..G...................................G...G........................ 1216 |
| PRCV-1894X-OR209253.1-USA ............C...............................................................T.......C..G...................................G...GG........T.............. 1216 |
| PRCV-1508712-III-NPTV-Parma-OR689863.1-Italy.................................C............................................T...........T..............................A.G.....C...................... 1216 |
| PRCV-86137004-X60089.1-UK .......................................................................................G................................................................ 1216 |
| PRCV-AR310-OR209251.1-USA ............C.......................................................................C..G...................................G...G........................ 1216 |
| PRCV-HOL87-M94097-Netherlands .......................................................................................G..T.......................T..................................... 1216 |
| PRCV-ISU-1-DQ811787.1-USA ............C................................................................G......C..G...................................G...G........................ 1216 |
| PRCV-ISU-1-OM830321.1-USA ............C................................................................G......C..G...................................G...G........................ 1216 |
| PRCV-ISU20-92330-OR209254.1-USA ............C............C.A........A..........T.........T..............C.....A.C............C..........................T..G...GT....................... 1216 |
| PRCV-KPRCV2401-PP781501.1-Korea ..............................T..........................A.................................CG..T..................T......A.G.....C...........T.......... 1216 |
| PRCV-KPRCV2402-PP781502.1-Korea ..............................T..........................A.................................CG..T..................T......A.G.....C...........T.......... 1216 |
| PRCV-KPRCV2403-PP781503.1-Korea ..............................T.............................................A..............CG..T..................T......A.G.....C...................... 1216 |
| PRCV-LEPP1-OR209252.1-USA ............C.......................................................................C..G...................................G...G........................ 1216 |
| PRCV-OH7269-KR270796.1-USA ............C............C.A...................T.........T..............C.....C.C...C..C................................T..G...GT....................... 1216 |
| PRCV-Minnesota-46140-KY406735.1-USA ............C.......................C......T.............T..........................C.TG...................................G...GG....................... 1216 |
| PRCV-NM-PV096984.1-China ............C.......................................................................C..G...................................G...G........................ 1216 |
| PRCV-RM4-Z24675.1-France .......................................................................................G................................................................ 1216 |
| TGEV-virulent-Purdue-DQ811789.2-USA ............C............................................T.........T................C..G.......T...............................G........................ 1216 |
| TGEV-Ly23-PQ189446.1-China ............C........................................................A..............C..G.......................G...............G........................ 1216 |
| TGEV-SouthDakota154-KX900411.1-USA ............C............................................T..........................C..C.TT....A..C.....................T..G...G........................ 1216 |
| TGEV-H16-FJ755618.2-China ............C.......................................................................C..G.......................G...............G........................ 1216 |
|  |
| Majority TCTATAATGGCACAGCTCTTAAGTATCTAGGAACATTACCACCTAGTGTCAAGGAGATTGCTATTAGTAAGTGGGGCCATTTTTATATTAATGGTTACAATTTCTTTAGCACATTTCCTATTGATTGTATATCTTTTAATTTGACTACTGGT |
| ---+---------+---------+---------+---------+---------+---------+---------+---------+---------+---------+---------+---------+---------+---------+-------- |
| 1220 1230 1240 1250 1260 1270 1280 1290 1300 1310 1320 1330 1340 1350 1360 |
| ---+---------+---------+---------+---------+---------+---------+---------+---------+---------+---------+---------+---------+---------+---------+-------- |
| PRCV-137isolate-86135308-OM830320.1-UK ........................................................................................................................................................ 1368 |
| PRCV-GXBS-B8-2023-PQ204803 .T...................................................................................................................................................... 1368 |
| PRCV-GXHZ-B1-2023-PQ204802 .T...................................................................................................................................................... 1368 |
| PRCV-GXHZ-K5-2023-PQ204804 .T...................................................................................................................................................... 1368 |
| PRCV-GXHZ-K17-2023-PQ204805 .T...................................................................................................................................................... 1368 |
| PRCV-GXLB-M66-2023-PQ204806 .T....................................................................................................................................................T. 1368 |
| PRCV-GXLB-M67-2023-PQ204807 .T....................................................................................................................................................T. 1368 |
| PRCV-GXLB-M68-2023-PQ204808 .T....................................................................................G...............................................................T. 1368 |
| PRCV-GXNN-W2-2023-PQ204800 .T....................................................................................................................................................T. 1368 |
| PRCV-GXNN-W4-2023-PQ204801 .T....................................................................................................................................................T. 1368 |
| PRCV-GXNN-X35-2024-PQ204809 .T.........................C............................................................................................................................ 1368 |
| PRCV-GXNN-X83-2022-PQ204794 .T...................................................................................................................................................... 1368 |
| PRCV-GXNN-X103-2022-PQ204795 .T...................................................................................................................................................... 1368 |
| PRCV-GXNN-X168-2022-PQ204796 .T...................................................................................................................................................... 1368 |
| PRCV-GXNN-X227-2022-PQ204797 .T...................................................................................................................................................... 1368 |
| PRCV-GXNN-X232-2022-PQ204798 .T...................................................................................................................................................... 1368 |
| PRCV-GXNN-X238-2024-PQ204810 .T...............................................................................................................................................C...... 1368 |
| PRCV-GXNN-X259-2022-PQ204799 .T...................................................................................................................................................... 1368 |
| PRCV-90-DK-OK078898.1-Denmark ........................................................................................................................................................ 1368 |
| PRCV-91V44-OR689864.1-Belgium .G...................................................................................................................................................... 1368 |
| PRCV-135isolate-86135308-OM830318.1-UK ........................................................................................................................................................ 1368 |
| PRCV-310isolate-AR310-OM830319.1-USA ..........................T.............................................................................................................C........C...... 1368 |
| PRCV-1894X-OR209253.1-USA ........................................................................................................................................C........C...... 1368 |
| PRCV-1508712-III-NPTV-Parma-OR689863.1-Italy.T...............................................................................................................................................G...... 1368 |
| PRCV-86137004-X60089.1-UK ........................................................................................................................................................ 1368 |
| PRCV-AR310-OR209251.1-USA ..........................T.............................................................................................................C........C...... 1368 |
| PRCV-HOL87-M94097-Netherlands ........................................................................................................................................................ 1368 |
| PRCV-ISU-1-DQ811787.1-USA ..........................T.............................................................................................................C........C...... 1368 |
| PRCV-ISU-1-OM830321.1-USA ..........................T.............................................................................................................C........C...... 1368 |
| PRCV-ISU20-92330-OR209254.1-USA ..........T........C......T......................T......................................................................................C........C...... 1368 |
| PRCV-KPRCV2401-PP781501.1-Korea ..............T..............................T.................................C.................T...............................................G...... 1368 |
| PRCV-KPRCV2402-PP781502.1-Korea ..............T..............................T.................................C.................T...............................................G...... 1368 |
| PRCV-KPRCV2403-PP781503.1-Korea ..............TT..............................A................................A.................................................................G...... 1368 |
| PRCV-LEPP1-OR209252.1-USA ..........................T.............................................................................................................C........C...... 1368 |
| PRCV-OH7269-KR270796.1-USA .T........T........C..A...T.............................................................................................................C........C...... 1368 |
| PRCV-Minnesota-46140-KY406735.1-USA ..........................T......................T............G.........................................................................C........C..G... 1368 |
| PRCV-NM-PV096984.1-China ..........................T....................................................C........................................................C........C...... 1368 |
| PRCV-RM4-Z24675.1-France ........................................................................................................................................................ 1368 |
| TGEV-virulent-Purdue-DQ811789.2-USA ..........................T......................................................................................................................C...... 1368 |
| TGEV-Ly23-PQ189446.1-China A...........................................GT...................................................................................................C...... 1368 |
| TGEV-SouthDakota154-KX900411.1-USA .............C............T.............................................................................................................C..C.....C.....C 1368 |
| TGEV-H16-FJ755618.2-China ............................................GT...................................................................................................C...... 1368 |
|  |
| Majority GATAGTGACGTCTTCTGGACAATAGCTTACACATCGTACACTGAAGCGTTAGTACAAGTTGAAAACACAGCTATTACAAATGTGACGTATTGTAATAGTTATGTTAATAACATTAAATGCTCTCAACTTACTGCTAATTTGAATAATGGATT |
| -+---------+---------+---------+---------+---------+---------+---------+---------+---------+---------+---------+---------+---------+---------+---------+ |
| 1370 1380 1390 1400 1410 1420 1430 1440 1450 1460 1470 1480 1490 1500 1510 1520 |
| -+---------+---------+---------+---------+---------+---------+---------+---------+---------+---------+---------+---------+---------+---------+---------+ |
| PRCV-137isolate-86135308-OM830320.1-UK ...............................................A........................................................................................................ 1520 |
| PRCV-GXBS-B8-2023-PQ204803 ...G...................................................................................................................T................C.......G....... 1520 |
| PRCV-GXHZ-B1-2023-PQ204802 ...G...................................................................................................................T................C.......G....... 1520 |
| PRCV-GXHZ-K5-2023-PQ204804 ...G...................................................................................................................T................C.......G....... 1520 |
| PRCV-GXHZ-K17-2023-PQ204805 ...G...................................................................................................................T................C.......G....... 1520 |
| PRCV-GXLB-M66-2023-PQ204806 ................................................................................................................................................G....... 1520 |
| PRCV-GXLB-M67-2023-PQ204807 ...................................T..T........A................................................................................................G....... 1520 |
| PRCV-GXLB-M68-2023-PQ204808 .............................................................................................................G..................................G....... 1520 |
| PRCV-GXNN-W2-2023-PQ204800 ................................................................................................................................................G....... 1520 |
| PRCV-GXNN-W4-2023-PQ204801 ................................................................................................................................................G....... 1520 |
| PRCV-GXNN-X35-2024-PQ204809 ...G...................................................................................................................T................C.......G....... 1520 |
| PRCV-GXNN-X83-2022-PQ204794 ...................................T..T........A.......................................................................T............T...C.......G....... 1520 |
| PRCV-GXNN-X103-2022-PQ204795 ..........................................................C............................................................T............T...C.......G....... 1520 |
| PRCV-GXNN-X168-2022-PQ204796 ...................................T..T........A.......................................................................T............T...C.......G....... 1520 |
| PRCV-GXNN-X227-2022-PQ204797 ...................................T..T........A.......................................................................T............T...C.......G....... 1520 |
| PRCV-GXNN-X232-2022-PQ204798 ...................................T..T........A.......................................................................T............T...C.......G....... 1520 |
| PRCV-GXNN-X238-2024-PQ204810 ...................................T..T........A.......................................................................T................C.......G....... 1520 |
| PRCV-GXNN-X259-2022-PQ204799 ...................................T..T........A.......................................................................T............T...C.......G....... 1520 |
| PRCV-90-DK-OK078898.1-Denmark ...............................................A...........................................................C............................................ 1520 |
| PRCV-91V44-OR689864.1-Belgium ..............T................................A...........................................................C............................................ 1520 |
| PRCV-135isolate-86135308-OM830318.1-UK ............................................C..A........................................................................................................ 1520 |
| PRCV-310isolate-AR310-OM830319.1-USA ...........T....................................................................G....................C.................................................. 1520 |
| PRCV-1894X-OR209253.1-USA ...........T.............................C......................................G....................C.....C............................................ 1520 |
| PRCV-1508712-III-NPTV-Parma-OR689863.1-Italy......A.......T................................A........................................................................................................ 1520 |
| PRCV-86137004-X60089.1-UK ...............................................A........................................................................................................ 1520 |
| PRCV-AR310-OR209251.1-USA ...........T....................................................................G....................C.................................................. 1520 |
| PRCV-HOL87-M94097-Netherlands ...............................................A........................................................................................................ 1520 |
| PRCV-ISU-1-DQ811787.1-USA ...........T....................................................................G....................C.................................................. 1520 |
| PRCV-ISU-1-OM830321.1-USA ...........T....................................................................G....................C.................................................. 1520 |
| PRCV-ISU20-92330-OR209254.1-USA ........T..T..........................T.........................................G....................C.....C............................................ 1520 |
| PRCV-KPRCV2401-PP781501.1-Korea ..............T................................A...........................................................C............................................ 1520 |
| PRCV-KPRCV2402-PP781502.1-Korea ..............T................................A...........................................................C............................................ 1520 |
| PRCV-KPRCV2403-PP781503.1-Korea ..............T................................A....................................................................................T................... 1520 |
| PRCV-LEPP1-OR209252.1-USA ...........T....................................................................G....................C.................................................. 1520 |
| PRCV-OH7269-KR270796.1-USA ........T..T....................................................................G....................C........T......................................... 1520 |
| PRCV-Minnesota-46140-KY406735.1-USA ...........T..................................T.................................G....................CA................................................. 1520 |
| PRCV-NM-PV096984.1-China ...........T.........................................................................................C.................................................. 1520 |
| PRCV-RM4-Z24675.1-France ...............................................A........................................................................................................ 1520 |
| TGEV-virulent-Purdue-DQ811789.2-USA ...........T.................T.................A................................G..................C.C.................................................. 1520 |
| TGEV-Ly23-PQ189446.1-China ...........T...................................A................................G....................C.................................................. 1520 |
| TGEV-SouthDakota154-KX900411.1-USA ...........T.....................................................T..............G....................C.................................................. 1520 |
| TGEV-H16-FJ755618.2-China ...........T...................................A................................G....................C.................................................. 1520 |
|  |
| Majority TTATCCTGTTTCTTCAAGTGAAGTTGGTTCTGTCAATAAGAGTGTTGTGTTACTACCTAGCTTTTTGACACATACCATTGTTAACATAACTATTGGTCTTGGTATGAAGCGTAGTGGTTATGGTCAACCCATAGCCTCTACGCTAAGTAACA |
| ---------+---------+---------+---------+---------+---------+---------+---------+---------+---------+---------+---------+---------+---------+---------+-- |
| 1530 1540 1550 1560 1570 1580 1590 1600 1610 1620 1630 1640 1650 1660 1670 |
| ---------+---------+---------+---------+---------+---------+---------+---------+---------+---------+---------+---------+---------+---------+---------+-- |
| PRCV-137isolate-86135308-OM830320.1-UK ................................................................C.........................................................................A............. 1672 |
| PRCV-GXBS-B8-2023-PQ204803 .................................T..C...............T...............................T...................................C............................... 1672 |
| PRCV-GXHZ-B1-2023-PQ204802 .................................T..C...............T...............................T...................................C............................... 1672 |
| PRCV-GXHZ-K5-2023-PQ204804 .................................T..C...............T...............................T...................................C............................... 1672 |
| PRCV-GXHZ-K17-2023-PQ204805 .................................T..C...............T...............................T...................................C............................... 1672 |
| PRCV-GXLB-M66-2023-PQ204806 .................................T..................................................T...................................C............................... 1672 |
| PRCV-GXLB-M67-2023-PQ204807 .................................T..................................................T...................................C............................... 1672 |
| PRCV-GXLB-M68-2023-PQ204808 .................................T..................................................T...................................C............................... 1672 |
| PRCV-GXNN-W2-2023-PQ204800 .................................T..................................................T...................................C..............T................ 1672 |
| PRCV-GXNN-W4-2023-PQ204801 .................................T..................................................T...................................C..............T................ 1672 |
| PRCV-GXNN-X35-2024-PQ204809 .................................T..C...............T...............................T...................................C............................... 1672 |
| PRCV-GXNN-X83-2022-PQ204794 .................................T..C........................C......................T...................................C............................... 1672 |
| PRCV-GXNN-X103-2022-PQ204795 .................................T..C...............................................T...................................C............................... 1672 |
| PRCV-GXNN-X168-2022-PQ204796 .................................T..C...............................................T...................................C............................... 1672 |
| PRCV-GXNN-X227-2022-PQ204797 .................................T..C...............................................T...................................C............................... 1672 |
| PRCV-GXNN-X232-2022-PQ204798 .................................T..C...............................................T...................................C............................... 1672 |
| PRCV-GXNN-X238-2024-PQ204810 .................................T..C.G.............T...............................T...................................C............................... 1672 |
| PRCV-GXNN-X259-2022-PQ204799 .................................T..C...............................................T...................................C............................... 1672 |
| PRCV-90-DK-OK078898.1-Denmark ................................................................C.........................................................................A............. 1672 |
| PRCV-91V44-OR689864.1-Belgium ................................................................C.........................................................................A............. 1672 |
| PRCV-135isolate-86135308-OM830318.1-UK ................................................................C.........................................................................A............. 1672 |
| PRCV-310isolate-AR310-OM830319.1-USA ............................CT...................................AC..G.........................A..........................................A..T.......... 1672 |
| PRCV-1894X-OR209253.1-USA ............................CT...................................AC.TG....................................................................A...T........G 1672 |
| PRCV-1508712-III-NPTV-Parma-OR689863.1-Italy................................................................C.........................................................................A............. 1672 |
| PRCV-86137004-X60089.1-UK ................................................................C.........................................................................A............. 1672 |
| PRCV-AR310-OR209251.1-USA ............................CT...................................AC..G.........................A..........................................A..T.......... 1672 |
| PRCV-HOL87-M94097-Netherlands ................................................................C.........................................................................A............. 1672 |
| PRCV-ISU-1-DQ811787.1-USA ............................CT...................................AC..G....................................................................A..T.......... 1672 |
| PRCV-ISU-1-OM830321.1-USA ............................CT...................................AC..G....................................................................A..T.......... 1672 |
| PRCV-ISU20-92330-OR209254.1-USA ............................CT.............................T...C.AT..G..C.................C..............................AA......T...........T.......... 1672 |
| PRCV-KPRCV2401-PP781501.1-Korea ......................A.....................................T...C....................................................................................... 1672 |
| PRCV-KPRCV2402-PP781502.1-Korea ......................A.....................................T...C....................................................................................... 1672 |
| PRCV-KPRCV2403-PP781503.1-Korea ......................A.....................................T...C....................................................................................... 1672 |
| PRCV-LEPP1-OR209252.1-USA ............................CT...................................AC..G.........................A..........................................A..T.......... 1672 |
| PRCV-OH7269-KR270796.1-USA ............................CT.............................T.....AT..G...........................................................T...........T.......... 1672 |
| PRCV-Minnesota-46140-KY406735.1-USA ............................CT..................................CAC..G..C.....................................T...........................A..TT......... 1672 |
| PRCV-NM-PV096984.1-China ............................CT.............................T....C....G.........................A..........................................A..T.......... 1672 |
| PRCV-RM4-Z24675.1-France ................................................................C.........................................................................A............. 1672 |
| TGEV-virulent-Purdue-DQ811789.2-USA ............................CT...................................AC............................A..........................................A..AT......... 1672 |
| TGEV-Ly23-PQ189446.1-China ............................CT...................................AC.......................................................................A..AT......... 1672 |
| TGEV-SouthDakota154-KX900411.1-USA ...C........C...............CT..............................T....AC..G...........C.............A..........................................AC.TT......... 1672 |
| TGEV-H16-FJ755618.2-China ............................CT...................................AC.......................................................................A..AT......... 1672 |
|  |
| Majority TTACACTACCAATGCAGGATAACAACACCGATGTGTACTGTGTTCGTTCTGACCAATTTTCAGTTTATGTTCATTCTACTTGCAAAAGTGCTTTATGGGACAATGTTTTTAAGCGAAACTGCACGGACGTTTTAGATGCCACAGCTGTTATA |
| -------+---------+---------+---------+---------+---------+---------+---------+---------+---------+---------+---------+---------+---------+---------+---- |
| 1680 1690 1700 1710 1720 1730 1740 1750 1760 1770 1780 1790 1800 1810 1820 |
| -------+---------+---------+---------+---------+---------+---------+---------+---------+---------+---------+---------+---------+---------+---------+---- |
| PRCV-137isolate-86135308-OM830320.1-UK ........................................................................................................................................................ 1824 |
| PRCV-GXBS-B8-2023-PQ204803 ...............................................................................................................G......................A..T.............. 1824 |
| PRCV-GXHZ-B1-2023-PQ204802 .........................T.....................................................................................G......................A..T.............. 1824 |
| PRCV-GXHZ-K5-2023-PQ204804 ...............................................................................................................G......................A..T.............. 1824 |
| PRCV-GXHZ-K17-2023-PQ204805 .........................T.....................................................................................G......................A..T.............. 1824 |
| PRCV-GXLB-M66-2023-PQ204806 ...........G...................................................................................................G.........................T.............. 1824 |
| PRCV-GXLB-M67-2023-PQ204807 ...........G..................................................................................................GG.........................T.............. 1824 |
| PRCV-GXLB-M68-2023-PQ204808 ...........G...................................................................................................G.........................T.............. 1824 |
| PRCV-GXNN-W2-2023-PQ204800 ...........G...................................................................................................G.........................T.............. 1824 |
| PRCV-GXNN-W4-2023-PQ204801 ...........G...................................................................................................G.........................T.............. 1824 |
| PRCV-GXNN-X35-2024-PQ204809 ...............................................................................................................G......................A..T.............. 1824 |
| PRCV-GXNN-X83-2022-PQ204794 .................................................C.............................................................G.A....................A..T.............. 1824 |
| PRCV-GXNN-X103-2022-PQ204795 .................................................C.............................................................G.A....................A..T.............. 1824 |
| PRCV-GXNN-X168-2022-PQ204796 .................................................C.............................................................G.A....................A..T.............. 1824 |
| PRCV-GXNN-X227-2022-PQ204797 .................................................C.............................................................G.A....................A..T.............. 1824 |
| PRCV-GXNN-X232-2022-PQ204798 .................................................C.............................................................G.A....................A..T.............. 1824 |
| PRCV-GXNN-X238-2024-PQ204810 ...............................................................................................................G......................A..T.............. 1824 |
| PRCV-GXNN-X259-2022-PQ204799 .................................................C.............................................................G.A....................A..T.............. 1824 |
| PRCV-90-DK-OK078898.1-Denmark .....................G...........................................C............G.............................................A........................... 1824 |
| PRCV-91V44-OR689864.1-Belgium ................................................................................................................................................T....... 1824 |
| PRCV-135isolate-86135308-OM830318.1-UK ..................C..................................................................................................................................... 1824 |
| PRCV-310isolate-AR310-OM830319.1-USA .........................................A..........T.............................T..................................................................... 1824 |
| PRCV-1894X-OR209253.1-USA ................................A........A..........T.............................T..................................................................... 1824 |
| PRCV-1508712-III-NPTV-Parma-OR689863.1-Italy.....................G...........................................C............G.............................................A........................... 1824 |
| PRCV-86137004-X60089.1-UK ........................................................................................................................................................ 1824 |
| PRCV-AR310-OR209251.1-USA .........................................A..........T.............................T..................................................................... 1824 |
| PRCV-HOL87-M94097-Netherlands ........................................................................................................................................................ 1824 |
| PRCV-ISU-1-DQ811787.1-USA ............C............................A...T......T.............................T...........G......................................................... 1824 |
| PRCV-ISU-1-OM830321.1-USA ............C............................A...T......T.............................T...........G......................................................... 1824 |
| PRCV-ISU20-92330-OR209254.1-USA ................A....................T...A........C.T....A........................T......T......................................A.......A.....T......... 1824 |
| PRCV-KPRCV2401-PP781501.1-Korea ...................C......C.................A.........................................................................T....................T............ 1824 |
| PRCV-KPRCV2402-PP781502.1-Korea ...................C......C.................A.........................................................................T....................T............ 1824 |
| PRCV-KPRCV2403-PP781503.1-Korea ...................C.................................................C................................................T...............A....T............ 1824 |
| PRCV-LEPP1-OR209252.1-USA .........................................A..........T.............................T..................................................................... 1824 |
| PRCV-OH7269-KR270796.1-USA ................A....................T...A........C.T.............................T...........................................................C......... 1824 |
| PRCV-Minnesota-46140-KY406735.1-USA ............CT.............AT........T...A..TT......T...............T.G...........T...................................................T.............C... 1824 |
| PRCV-NM-PV096984.1-China ...................C.....................A........C...............................T..................................................................... 1824 |
| PRCV-RM4-Z24675.1-France ...........................A..............................................................T............................................................. 1824 |
| TGEV-virulent-Purdue-DQ811789.2-USA .C.......................................A...............................................T..............A............................................... 1824 |
| TGEV-Ly23-PQ189446.1-China ....................G....................A.............................................................................................................. 1824 |
| TGEV-SouthDakota154-KX900411.1-USA ...............................C.........A..........T...................T......C..T.....................................................G............... 1824 |
| TGEV-H16-FJ755618.2-China ....................G....................A.............................................................................................................. 1824 |
|  |
| Majority AAAACTGGTACTTGTCCTTTCTCATTTGATAAATTGAACAATTACTTAACTTTTAACAAGTTCTGTTTGTCGTTGAGTCCTGTTGGTGCTAATTGTAAGTTTGATGTAGCTGCCCGTACAAGAACCAATGATCAGGTTGTTAGAAGTTTGTA |
| -----+---------+---------+---------+---------+---------+---------+---------+---------+---------+---------+---------+---------+---------+---------+------ |
| 1830 1840 1850 1860 1870 1880 1890 1900 1910 1920 1930 1940 1950 1960 1970 |
| -----+---------+---------+---------+---------+---------+---------+---------+---------+---------+---------+---------+---------+---------+---------+------ |
| PRCV-137isolate-86135308-OM830320.1-UK ................................................................................C......................................................T................ 1976 |
| PRCV-GXBS-B8-2023-PQ204803 .....C.....C...............T........................C.........................................................C......................................... 1976 |
| PRCV-GXHZ-B1-2023-PQ204802 .....C.....C...............T.........G............................................................T...........C......................................... 1976 |
| PRCV-GXHZ-K5-2023-PQ204804 .....C.....C...............T..................................................................................C......................................... 1976 |
| PRCV-GXHZ-K17-2023-PQ204805 .....C.....C...............T......................................................................T...........C......................................... 1976 |
| PRCV-GXLB-M66-2023-PQ204806 ...........C...............T..................................................................................C......................................... 1976 |
| PRCV-GXLB-M67-2023-PQ204807 ...........C...............T...................................................................C..............C......................................... 1976 |
| PRCV-GXLB-M68-2023-PQ204808 ...........C...............T..................................................................................C......................................... 1976 |
| PRCV-GXNN-W2-2023-PQ204800 ...........C...............T.......................................................C..........................C......................................... 1976 |
| PRCV-GXNN-W4-2023-PQ204801 ...........C...............T.......................................................C..........................C......................................... 1976 |
| PRCV-GXNN-X35-2024-PQ204809 .....C.....C...............T..................................................................................C......................................... 1976 |
| PRCV-GXNN-X83-2022-PQ204794 ...........C...............T..................................................................................C......................................... 1976 |
| PRCV-GXNN-X103-2022-PQ204795 ...........C...............T..................................................................................C......................................... 1976 |
| PRCV-GXNN-X168-2022-PQ204796 ...........C...............T..................................................................................C......................................... 1976 |
| PRCV-GXNN-X227-2022-PQ204797 ...........C...............T..................................................................................C......................................... 1976 |
| PRCV-GXNN-X232-2022-PQ204798 ...........C...............T..................................................................................C......................................... 1976 |
| PRCV-GXNN-X238-2024-PQ204810 .....C.....C...............T..................................................................................C......................................... 1976 |
| PRCV-GXNN-X259-2022-PQ204799 ...........C...............T..................................................................................C......................................... 1976 |
| PRCV-90-DK-OK078898.1-Denmark ........................................................A.....T.................C....................................................................... 1976 |
| PRCV-91V44-OR689864.1-Belgium ........................................................A.......................C....................................................................... 1976 |
| PRCV-135isolate-86135308-OM830318.1-UK ................................................................................C....................................................................... 1976 |
| PRCV-310isolate-AR310-OM830319.1-USA .................................C...........................................................................T.......................................... 1976 |
| PRCV-1894X-OR209253.1-USA ............................................................................A................................T.......................................... 1976 |
| PRCV-1508712-III-NPTV-Parma-OR689863.1-Italy........................................................A.....T.................C....................................................................... 1976 |
| PRCV-86137004-X60089.1-UK .......................................................................................................................................T................ 1976 |
| PRCV-AR310-OR209251.1-USA .................................C...........................................................................T.......................................... 1976 |
| PRCV-HOL87-M94097-Netherlands .......................................................................................................................C...........G.................... 1976 |
| PRCV-ISU-1-DQ811787.1-USA .................................C....T........................................................C.............T.......................................... 1976 |
| PRCV-ISU-1-OM830321.1-USA .................................C....T........................................................C.............T.......................................... 1976 |
| PRCV-ISU20-92330-OR209254.1-USA .................................C....T....................A........T.........T................C........C....TA......................................... 1976 |
| PRCV-KPRCV2401-PP781501.1-Korea .................C......................................A............................................................................................... 1976 |
| PRCV-KPRCV2402-PP781502.1-Korea .................C......................................A............................................................................................... 1976 |
| PRCV-KPRCV2403-PP781503.1-Korea .................C....................T.................A............................................................................................... 1976 |
| PRCV-LEPP1-OR209252.1-USA .................................C...........................................................................T.......................................... 1976 |
| PRCV-OH7269-KR270796.1-USA .................................C..................................T.........T..............................TA......................................... 1976 |
| PRCV-Minnesota-46140-KY406735.1-USA .................................C....T.............................T..........................C.............T........................A................. 1976 |
| PRCV-NM-PV096984.1-China .................................C...........................................................................T.......................................... 1976 |
| PRCV-RM4-Z24675.1-France ................................................................................C....................................................................... 1976 |
| TGEV-virulent-Purdue-DQ811789.2-USA ...................................................................................................................................G.................... 1976 |
| TGEV-Ly23-PQ189446.1-China ............................C........................................................................................................................... 1976 |
| TGEV-SouthDakota154-KX900411.1-USA .................................C..........................C.......T........................................T.......................................... 1976 |
| TGEV-H16-FJ755618.2-China ............................C........................................................................................................................... 1976 |
|  |
| Majority TGTAATATATGAAGAAGGAGACAGCATAGTTGGTGTACCGTCTGACAATAGTGGTTTGCACGATTTGTCAGTGCTACACCTAGATTCATGCACAGATTACAATATATATGGTAGAACTGGTGTTGGTATTATTAGACAAACTAACAGGACGC |
| ---+---------+---------+---------+---------+---------+---------+---------+---------+---------+---------+---------+---------+---------+---------+-------- |
| 1980 1990 2000 2010 2020 2030 2040 2050 2060 2070 2080 2090 2100 2110 2120 |
| ---+---------+---------+---------+---------+---------+---------+---------+---------+---------+---------+---------+---------+---------+---------+-------- |
| PRCV-137isolate-86135308-OM830320.1-UK .......................................................................................G................................................................ 2128 |
| PRCV-GXBS-B8-2023-PQ204803 .......................................T..C............................................................................................................. 2128 |
| PRCV-GXHZ-B1-2023-PQ204802 .......................................T..C............................................................................................................. 2128 |
| PRCV-GXHZ-K5-2023-PQ204804 .......................................T..C............................................................................................................. 2128 |
| PRCV-GXHZ-K17-2023-PQ204805 .......................................T..C............................................................................................................. 2128 |
| PRCV-GXLB-M66-2023-PQ204806 .......................................T..C..T.....................................................T.................................................... 2128 |
| PRCV-GXLB-M67-2023-PQ204807 .......................................T..C..T.....................................................T..................................................A. 2128 |
| PRCV-GXLB-M68-2023-PQ204808 .......................................T..C..T.....................................................T.................................................... 2128 |
| PRCV-GXNN-W2-2023-PQ204800 .......................................T..C..T.......................................................................................................... 2128 |
| PRCV-GXNN-W4-2023-PQ204801 .......................................T..C..T.................................................................A........................................ 2128 |
| PRCV-GXNN-X35-2024-PQ204809 .......................................T..C............................................................................................................. 2128 |
| PRCV-GXNN-X83-2022-PQ204794 .......................................T..C............................................................................................................. 2128 |
| PRCV-GXNN-X103-2022-PQ204795 .......................................T..C............................................................................................................. 2128 |
| PRCV-GXNN-X168-2022-PQ204796 .......................................T..C............................................................................................................. 2128 |
| PRCV-GXNN-X227-2022-PQ204797 .......................................T..C............................................................................................................. 2128 |
| PRCV-GXNN-X232-2022-PQ204798 .......................................T..C............................................................................................................. 2128 |
| PRCV-GXNN-X238-2024-PQ204810 .......................................T..C............................................................................................................. 2128 |
| PRCV-GXNN-X259-2022-PQ204799 .......................................T..C............................................................................................................. 2128 |
| PRCV-90-DK-OK078898.1-Denmark .......................................................................................G................................................................ 2128 |
| PRCV-91V44-OR689864.1-Belgium ..................................A....................................................G................................C............................... 2128 |
| PRCV-135isolate-86135308-OM830318.1-UK .......................................................................................G................................................................ 2128 |
| PRCV-310isolate-AR310-OM830319.1-USA .......................A......A.......................................................................................................................A. 2128 |
| PRCV-1894X-OR209253.1-USA .......................A......G.......................................................................................................................A. 2128 |
| PRCV-1508712-III-NPTV-Parma-OR689863.1-Italy.......................................................................................G................................................................ 2128 |
| PRCV-86137004-X60089.1-UK .......................................................................................G................................................................ 2128 |
| PRCV-AR310-OR209251.1-USA .......................A......A.......................................................................................................................A. 2128 |
| PRCV-HOL87-M94097-Netherlands .......................................................................................G................................................................ 2128 |
| PRCV-ISU-1-DQ811787.1-USA .......................A......A........T.............................................................................C................................A. 2128 |
| PRCV-ISU-1-OM830321.1-USA .......................A......A........T.............................................................................C................................A. 2128 |
| PRCV-ISU20-92330-OR209254.1-USA ...............G.......A......A.....................C.C....................T..........................................................................A. 2128 |
| PRCV-KPRCV2401-PP781501.1-Korea ............................................C..........C..........C....................G................................CT.............................. 2128 |
| PRCV-KPRCV2402-PP781502.1-Korea ............................................C..........C..........C....................G................................CT.............................. 2128 |
| PRCV-KPRCV2403-PP781503.1-Korea .......................................T...............................................G.................................T............................T. 2128 |
| PRCV-LEPP1-OR209252.1-USA .......................A......A.......................................................................................................................A. 2128 |
| PRCV-OH7269-KR270796.1-USA .......................A......A.....................C.C....................T..........................................................................A. 2128 |
| PRCV-Minnesota-46140-KY406735.1-USA .......................A....A.A........................G.T.........................................T.................C................................A. 2128 |
| PRCV-NM-PV096984.1-China .......................A......A.......................................................................................................................A. 2128 |
| PRCV-RM4-Z24675.1-France .......................................................................................G...............................................................A 2128 |
| TGEV-virulent-Purdue-DQ811789.2-USA .......................A......G..............T.........................................C................................................................ 2128 |
| TGEV-Ly23-PQ189446.1-China .......................A......G..............T...........A.............................C................................................................ 2128 |
| TGEV-SouthDakota154-KX900411.1-USA .......................A......A................................C.....G................................................................................A. 2128 |
| TGEV-H16-FJ755618.2-China .......................A......G..............T...........A.............................C................................................................ 2128 |
|  |
| Majority TACTTAGTGGCTTATATTACACATCACTATCTGGTGATTTGTTAGGTTTTAAAAATGTTAGTGATGGTGTTATCTACTCTGTAACGCCATGTGATGTTAGCGCACAAGCAGCTGTTATTGATGGTACCATAGTTGGGGCTATCACTTCCATT |
| -+---------+---------+---------+---------+---------+---------+---------+---------+---------+---------+---------+---------+---------+---------+---------+ |
| 2130 2140 2150 2160 2170 2180 2190 2200 2210 2220 2230 2240 2250 2260 2270 2280 |
| -+---------+---------+---------+---------+---------+---------+---------+---------+---------+---------+---------+---------+---------+---------+---------+ |
| PRCV-137isolate-86135308-OM830320.1-UK .................................................................................................................A...................................... 2280 |
| PRCV-GXBS-B8-2023-PQ204803 ......................................................................................................................C................................. 2280 |
| PRCV-GXHZ-B1-2023-PQ204802 ......................................................................................................................C................................. 2280 |
| PRCV-GXHZ-K5-2023-PQ204804 ......................................................................................................................C................................. 2280 |
| PRCV-GXHZ-K17-2023-PQ204805 ..................................................................................................................G...C................................. 2280 |
| PRCV-GXLB-M66-2023-PQ204806 ........................................................................................................................................................ 2280 |
| PRCV-GXLB-M67-2023-PQ204807 ......................................................................CG................................................................................ 2280 |
| PRCV-GXLB-M68-2023-PQ204808 ........................................................................................................................................................ 2280 |
| PRCV-GXNN-W2-2023-PQ204800 ...C.................................................................................AA................................................................. 2280 |
| PRCV-GXNN-W4-2023-PQ204801 ...C.................................................................................................................................................... 2280 |
| PRCV-GXNN-X35-2024-PQ204809 ......................................................................................................................C................................. 2280 |
| PRCV-GXNN-X83-2022-PQ204794 .............G.......................................................................................................................................... 2280 |
| PRCV-GXNN-X103-2022-PQ204795 .............G.......................................................................................................................................... 2280 |
| PRCV-GXNN-X168-2022-PQ204796 .............G.......................................................................................................................................... 2280 |
| PRCV-GXNN-X227-2022-PQ204797 .............G.......................................................................................................................................... 2280 |
| PRCV-GXNN-X232-2022-PQ204798 .............G.......................................................................................................................................... 2280 |
| PRCV-GXNN-X238-2024-PQ204810 ......................................................................................................................C................................. 2280 |
| PRCV-GXNN-X259-2022-PQ204799 .............G.......................................................................................................................................... 2280 |
| PRCV-90-DK-OK078898.1-Denmark .................................................................................................................A.C.................................... 2280 |
| PRCV-91V44-OR689864.1-Belgium .................................................................................................................A......................T............... 2280 |
| PRCV-135isolate-86135308-OM830318.1-UK .................................................................................................................A...................................... 2280 |
| PRCV-310isolate-AR310-OM830319.1-USA ........................................................................................G........A..................................................T... 2280 |
| PRCV-1894X-OR209253.1-USA .................................................................................................A..................................................T... 2280 |
| PRCV-1508712-III-NPTV-Parma-OR689863.1-Italy.................................................................................................................A.C.................................... 2280 |
| PRCV-86137004-X60089.1-UK .................................................................................................................A...........G.......................... 2280 |
| PRCV-AR310-OR209251.1-USA ........................................................................................G........A..................................................T... 2280 |
| PRCV-HOL87-M94097-Netherlands ........................................................................................................................................................ 2280 |
| PRCV-ISU-1-DQ811787.1-USA ........................................................................................G........A..................................................T... 2280 |
| PRCV-ISU-1-OM830321.1-USA ........................................................................................G........A..................................................T... 2280 |
| PRCV-ISU20-92330-OR209254.1-USA ............................................................................T....................A.....................A................A...........T... 2280 |
| PRCV-KPRCV2401-PP781501.1-Korea ..........T......................................C...............................................................A...........G...................C..T... 2280 |
| PRCV-KPRCV2402-PP781502.1-Korea ..........T......................................C...............................................................A...........G...................C..T... 2280 |
| PRCV-KPRCV2403-PP781503.1-Korea ..T.......T......................................C...............................................................A...........G......................T... 2280 |
| PRCV-LEPP1-OR209252.1-USA ........................................................................................G........A..................................................T... 2280 |
| PRCV-OH7269-KR270796.1-USA ............................................................................T....................A.....................A................T...........T... 2280 |
| PRCV-Minnesota-46140-KY406735.1-USA .................................................C.............................C........G........A.........................A.........A..............T... 2280 |
| PRCV-NM-PV096984.1-China ....................................................................................................................................................T... 2280 |
| PRCV-RM4-Z24675.1-France ...................................................C.............................................................A...................................... 2280 |
| TGEV-virulent-Purdue-DQ811789.2-USA ...............................A......................................C..........................A...................................................... 2280 |
| TGEV-Ly23-PQ189446.1-China ...............................A......................................C..........................A...................................................... 2280 |
| TGEV-SouthDakota154-KX900411.1-USA .................................................C.................C..C.................G........A..................................................T... 2280 |
| TGEV-H16-FJ755618.2-China ...............................A......................................C..........................A.T.................................................... 2280 |
|  |
| Majority AACAGTGAATTGTTAGGTCTAACACATTGGACAACAACACCTAATTTTTATTACTACTCTATATATAATTACACAAATGATAAGACTCGTGGCACTCCAATTGGCAGTAATGACGTTGATTGTGAACCTGTCATAACCTATTCTAACATAGG |
| ---------+---------+---------+---------+---------+---------+---------+---------+---------+---------+---------+---------+---------+---------+---------+-- |
| 2290 2300 2310 2320 2330 2340 2350 2360 2370 2380 2390 2400 2410 2420 2430 |
| ---------+---------+---------+---------+---------+---------+---------+---------+---------+---------+---------+---------+---------+---------+---------+-- |
| PRCV-137isolate-86135308-OM830320.1-UK ..................................T..................................................................................................................... 2432 |
| PRCV-GXBS-B8-2023-PQ204803 .....C..................................................T..........................................T.C....T...............C............................. 2432 |
| PRCV-GXHZ-B1-2023-PQ204802 ........................................................T..........................................T.C....T...............C............................. 2432 |
| PRCV-GXHZ-K5-2023-PQ204804 ........................................................T..........................................T.C....T...............C............................. 2432 |
| PRCV-GXHZ-K17-2023-PQ204805 ........................................................T..........................................T.C....T...............C............................. 2432 |
| PRCV-GXLB-M66-2023-PQ204806 ........................................................T............................A.............T......T...............C............................. 2432 |
| PRCV-GXLB-M67-2023-PQ204807 .......................G................................T............................A.............T......T...............C............................. 2432 |
| PRCV-GXLB-M68-2023-PQ204808 ........................................................T............................A.............T......T...............C............................. 2432 |
| PRCV-GXNN-W2-2023-PQ204800 ............................................C...........T............................A.............T......T...............C............................. 2432 |
| PRCV-GXNN-W4-2023-PQ204801 ............................................C...........T............................A.............T......T...............C............................. 2432 |
| PRCV-GXNN-X35-2024-PQ204809 ..................................T.....................T..........................................T.C....T...............C............................. 2432 |
| PRCV-GXNN-X83-2022-PQ204794 ........................................................T..........................................T......T...............C.......................T..... 2432 |
| PRCV-GXNN-X103-2022-PQ204795 ........................................................T..........................................T......T...............C.......................T..... 2432 |
| PRCV-GXNN-X168-2022-PQ204796 ........................................................T..........................................T......T...............C.......................T..... 2432 |
| PRCV-GXNN-X227-2022-PQ204797 ........................................................T..........................................T......T...............C.......................T..... 2432 |
| PRCV-GXNN-X232-2022-PQ204798 ........................................................T..........................................T......T...............C.......................T..... 2432 |
| PRCV-GXNN-X238-2024-PQ204810 ........................................................T..........................................T.C....T...............C............................. 2432 |
| PRCV-GXNN-X259-2022-PQ204799 ......................................G.................T..........................................T......T...............C.......................T..... 2432 |
| PRCV-90-DK-OK078898.1-Denmark ..................................T...........................T........................................A................................................ 2432 |
| PRCV-91V44-OR689864.1-Belgium ..................................T....................................................................A................................................ 2432 |
| PRCV-135isolate-86135308-OM830318.1-UK ..................................T..................................................................................................................... 2432 |
| PRCV-310isolate-AR310-OM830319.1-USA ................................G.................................................G..T..........G......A................................................ 2432 |
| PRCV-1894X-OR209253.1-USA ..................T..................................T............................G.............G......A................................................ 2432 |
| PRCV-1508712-III-NPTV-Parma-OR689863.1-Italy..................................T...........................T........................................A................................................ 2432 |
| PRCV-86137004-X60089.1-UK ................C.................T..................................................................................................................... 2432 |
| PRCV-AR310-OR209251.1-USA ................................G.................................................G..T..........G......A................................................ 2432 |
| PRCV-HOL87-M94097-Netherlands ..................................T....................................................................A..............G................................. 2432 |
| PRCV-ISU-1-DQ811787.1-USA ................................G.................................................G..T..........G......A................................................ 2432 |
| PRCV-ISU-1-OM830321.1-USA ................................G.................................................G..T..........G......A................................................ 2432 |
| PRCV-ISU20-92330-OR209254.1-USA ..................T....G..C.....G..T..............................................GA.T..........G......A.........T...................................... 2432 |
| PRCV-KPRCV2401-PP781501.1-Korea ..................................T..................T.............................A...................A...........C.................................... 2432 |
| PRCV-KPRCV2402-PP781502.1-Korea ..................................T..................T.............................A...................A...........C.................................... 2432 |
| PRCV-KPRCV2403-PP781503.1-Korea ..................................T..................T.............................A.......A...........A...........C.................................... 2432 |
| PRCV-LEPP1-OR209252.1-USA ................................G.................................................G..T..........G......A................................................ 2432 |
| PRCV-OH7269-KR270796.1-USA ..................T....G........G..T...........C.......................T..........GA.T..........GT.....A.........T...................................... 2432 |
| PRCV-Minnesota-46140-KY406735.1-USA ........G.......................GC.....................................T......T...GA.T..........G......A.........T...................................... 2432 |
| PRCV-NM-PV096984.1-China ................................G.................................................G..T..........G......A................................................ 2432 |
| PRCV-RM4-Z24675.1-France ........................................................................................................................................................ 2432 |
| TGEV-virulent-Purdue-DQ811789.2-USA .........C........................................................................G.............G......A.........T...................................... 2432 |
| TGEV-Ly23-PQ189446.1-China .........C........................................................................G.............G......A.........---.................................... 2432 |
| TGEV-SouthDakota154-KX900411.1-USA .........C.............G........C.................................................G..T..........G......A......C.T....................................... 2432 |
| TGEV-H16-FJ755618.2-China .........C........................................................................G.............G......A.........---.................................... 2432 |
|  |
| Majority TGTTTGTAAAAATGGTGCTTTGGTTTTTATTAACGTCACACATTCTGATGGAGACGTGCAACCAATTAGCACTGGTAACGTCACGATACCTACTAATTTTACTATATCCGTGCAAGTCGAATACATTCAGGTTTACACTACACCAGTGTCAA |
| -------+---------+---------+---------+---------+---------+---------+---------+---------+---------+---------+---------+---------+---------+---------+---- |
| 2440 2450 2460 2470 2480 2490 2500 2510 2520 2530 2540 2550 2560 2570 2580 |
| -------+---------+---------+---------+---------+---------+---------+---------+---------+---------+---------+---------+---------+---------+---------+---- |
| PRCV-137isolate-86135308-OM830320.1-UK ................................................................................................C....................................................... 2584 |
| PRCV-GXBS-B8-2023-PQ204803 .........T...................................G................................T..........................C.............................................. 2584 |
| PRCV-GXHZ-B1-2023-PQ204802 .........T.........................A.........G................................T..........................C.............................................. 2584 |
| PRCV-GXHZ-K5-2023-PQ204804 .........T...................................G................................T..........................C.............................................. 2584 |
| PRCV-GXHZ-K17-2023-PQ204805 .........T...................................G................................T..........................C......................C....................... 2584 |
| PRCV-GXLB-M66-2023-PQ204806 .........T...................................G................................T..........................C.........................................T.... 2584 |
| PRCV-GXLB-M67-2023-PQ204807 .........T...................................G................................T..........................C.........................................T.... 2584 |
| PRCV-GXLB-M68-2023-PQ204808 .........T...................................G................................T..........................C.........................................T.... 2584 |
| PRCV-GXNN-W2-2023-PQ204800 .........T...................................G................................T..........................C.............................................. 2584 |
| PRCV-GXNN-W4-2023-PQ204801 .........T...................................G................................T..........................C.............................................. 2584 |
| PRCV-GXNN-X35-2024-PQ204809 .........T...................................G................................T..........................C.............................................. 2584 |
| PRCV-GXNN-X83-2022-PQ204794 .........T...................................G...........................................................C.............................................. 2584 |
| PRCV-GXNN-X103-2022-PQ204795 .........T...................................G...........................................................C.............................................. 2584 |
| PRCV-GXNN-X168-2022-PQ204796 .........T...................................G...........................................................C.............................................. 2584 |
| PRCV-GXNN-X227-2022-PQ204797 .........T...................................G...........................................................C.............................................. 2584 |
| PRCV-GXNN-X232-2022-PQ204798 .........T...................................G...........................................................C.............................................. 2584 |
| PRCV-GXNN-X238-2024-PQ204810 .........T...................................G................................T..........................C.............................................. 2584 |
| PRCV-GXNN-X259-2022-PQ204799 .........T...................................G...........................................................C.............................................. 2584 |
| PRCV-90-DK-OK078898.1-Denmark ................................................................................................C....................................................... 2584 |
| PRCV-91V44-OR689864.1-Belgium .A..............................................................................................C....................................................... 2584 |
| PRCV-135isolate-86135308-OM830318.1-UK ................................................................................................C....................................................... 2584 |
| PRCV-310isolate-AR310-OM830319.1-USA ............C................................................................................A.......................................................... 2584 |
| PRCV-1894X-OR209253.1-USA ............C..................................C.............................................A..............T........................................... 2584 |
| PRCV-1508712-III-NPTV-Parma-OR689863.1-Italy...............................................T.....................T..........................C....................................................... 2584 |
| PRCV-86137004-X60089.1-UK ................................................................................................C....................................................... 2584 |
| PRCV-AR310-OR209251.1-USA ............C................................................................................A.......................................................... 2584 |
| PRCV-HOL87-M94097-Netherlands ..................................................................A...........T.................C....................................................... 2584 |
| PRCV-ISU-1-DQ811787.1-USA ............C.......................T........................................................A.......................................................... 2584 |
| PRCV-ISU-1-OM830321.1-USA ............C.......................T........................................................A.......................................................... 2584 |
| PRCV-ISU20-92330-OR209254.1-USA .........................................................C............G.......T..T..A........A..........................G............................... 2584 |
| PRCV-KPRCV2401-PP781501.1-Korea .A..............................................A................................A..............C................................A...................... 2584 |
| PRCV-KPRCV2402-PP781502.1-Korea .A..............................................A................................A..............C................................A...................... 2584 |
| PRCV-KPRCV2403-PP781503.1-Korea .A............................................C.A.............................T..A..............C................................A...................... 2584 |
| PRCV-LEPP1-OR209252.1-USA ............C................................................................................A.......................................................... 2584 |
| PRCV-OH7269-KR270796.1-USA ............C............................................T............G.......T.....A........A..........................G............................... 2584 |
| PRCV-Minnesota-46140-KY406735.1-USA ............C......................................G.....T............G.......T.....A........A..........................G............................... 2584 |
| PRCV-NM-PV096984.1-China ............C................................................................................A.......................................................... 2584 |
| PRCV-RM4-Z24675.1-France ................................................................................................C....................................................... 2584 |
| TGEV-virulent-Purdue-DQ811789.2-USA .....................T........................................................T..............A..C.....C....................T............................ 2584 |
| TGEV-Ly23-PQ189446.1-China .............................................................................................A..C..........................T............................ 2584 |
| TGEV-SouthDakota154-KX900411.1-USA ............C.................C..........................T...................................A.......................T.................................. 2584 |
| TGEV-H16-FJ755618.2-China .............................................................................................A..C..........................T............................ 2584 |
|  |
| Majority TAGACTGTTCAAGATATGTTTGTAATGGCAACCCTAGGTGTAACAAACTGTTAACACAATACGTTTCTGCATGTCAAACTATTGAGCAAGCACTTGCAATGGGTGCCAGACTTGAAAACATGGAAGTTGATTCCATGTTATTTGTTTCTGAA |
| -----+---------+---------+---------+---------+---------+---------+---------+---------+---------+---------+---------+---------+---------+---------+------ |
| 2590 2600 2610 2620 2630 2640 2650 2660 2670 2680 2690 2700 2710 2720 2730 |
| -----+---------+---------+---------+---------+---------+---------+---------+---------+---------+---------+---------+---------+---------+---------+------ |
| PRCV-137isolate-86135308-OM830320.1-UK ........................................................................................................................................................ 2736 |
| PRCV-GXBS-B8-2023-PQ204803 ...........................................T.................T..........................G.................T.................G........................... 2736 |
| PRCV-GXHZ-B1-2023-PQ204802 ...........................................T.................T..........................G.................T.................G........................... 2736 |
| PRCV-GXHZ-K5-2023-PQ204804 ...........................................T.................T..........................G.................T.................G........................... 2736 |
| PRCV-GXHZ-K17-2023-PQ204805 ...........................................T.................T..........................G.................T.................G........................... 2736 |
| PRCV-GXLB-M66-2023-PQ204806 ...........................................T............................................G.................T.................G........................... 2736 |
| PRCV-GXLB-M67-2023-PQ204807 ...........................................T............................................G.................T.................G........................... 2736 |
| PRCV-GXLB-M68-2023-PQ204808 ...........................................T............................................G.................T.................G........................... 2736 |
| PRCV-GXNN-W2-2023-PQ204800 ...........................................T............................................G...................................G........................... 2736 |
| PRCV-GXNN-W4-2023-PQ204801 ...........................................T............................................G...................................G........................... 2736 |
| PRCV-GXNN-X35-2024-PQ204809 ...........................................T.................T..........................G.................T.................G........................... 2736 |
| PRCV-GXNN-X83-2022-PQ204794 ........................................................................................G.................T.................G........................... 2736 |
| PRCV-GXNN-X103-2022-PQ204795 ..................................................C.....................................G.................T.................G........................... 2736 |
| PRCV-GXNN-X168-2022-PQ204796 ........................................................................................G.................T.................G........................... 2736 |
| PRCV-GXNN-X227-2022-PQ204797 ..................................................C.....................................G.................T.................G........................... 2736 |
| PRCV-GXNN-X232-2022-PQ204798 ..................................................C.....................................G.................T.................G........................... 2736 |
| PRCV-GXNN-X238-2024-PQ204810 ...........................................T.................T..........................G.................T.................G........................... 2736 |
| PRCV-GXNN-X259-2022-PQ204799 ........................................................................................G.................T.................G........................... 2736 |
| PRCV-90-DK-OK078898.1-Denmark ........................................................................................................................................................ 2736 |
| PRCV-91V44-OR689864.1-Belgium ...............................T........................................................................................................................ 2736 |
| PRCV-135isolate-86135308-OM830318.1-UK ........................................................................................................................................................ 2736 |
| PRCV-310isolate-AR310-OM830319.1-USA ...............................................T.A...........T............................................T...........T................................. 2736 |
| PRCV-1894X-OR209253.1-USA .................................................A....................................................................T................................. 2736 |
| PRCV-1508712-III-NPTV-Parma-OR689863.1-Italy...............................T...............T..............................A......................................................................... 2736 |
| PRCV-86137004-X60089.1-UK ........................................................................................................................................................ 2736 |
| PRCV-AR310-OR209251.1-USA ...............................................T.A...........T............................................T...........T................................. 2736 |
| PRCV-HOL87-M94097-Netherlands ........................................................................................................................................................ 2736 |
| PRCV-ISU-1-DQ811787.1-USA .................................................A...........T.......................................A................T................................. 2736 |
| PRCV-ISU-1-OM830321.1-USA .................................................A...........T.......................................A................T................................. 2736 |
| PRCV-ISU20-92330-OR209254.1-USA .................................................A...........TA.......................................................T..............T.................. 2736 |
| PRCV-KPRCV2401-PP781501.1-Korea ...............................T...............T.............T............................................A............................................. 2736 |
| PRCV-KPRCV2402-PP781502.1-Korea ...............................T...............T.............T............................................A............................................. 2736 |
| PRCV-KPRCV2403-PP781503.1-Korea .......C.......................T...............T.............T............................................A............................................. 2736 |
| PRCV-LEPP1-OR209252.1-USA ...............................................T.A...........T............................................T...........T................................. 2736 |
| PRCV-OH7269-KR270796.1-USA .................................................A...........TA.......................................................T................................. 2736 |
| PRCV-Minnesota-46140-KY406735.1-USA .................................................A...........T........................................................T................................. 2736 |
| PRCV-NM-PV096984.1-China ...............................................T.A...........T............................................T...........T................................. 2736 |
| PRCV-RM4-Z24675.1-France ........................................................................................................................................................ 2736 |
| TGEV-virulent-Purdue-DQ811789.2-USA ............................T..................T............................................................................G..............G............ 2736 |
| TGEV-Ly23-PQ189446.1-China ...............................................T........................................................................................................ 2736 |
| TGEV-SouthDakota154-KX900411.1-USA .................................................A...........T........................................................T................................. 2736 |
| TGEV-H16-FJ755618.2-China ...............................................T........................................................................................................ 2736 |
|  |
| Majority AATGCCCTTAAATTGGCTTCTGTTGAAGCATTCAATAGTTCAGAAACTTTAGATCCTATTTACAAAGAATGGCCTAATATAGGTGGCTTTTGGCTAGAAGGTCTAAAATACATACTCCCGTCCGATAATAGCAAACGTAAGTATCGTTCAGC |
| ---+---------+---------+---------+---------+---------+---------+---------+---------+---------+---------+---------+---------+---------+---------+-------- |
| 2740 2750 2760 2770 2780 2790 2800 2810 2820 2830 2840 2850 2860 2870 2880 |
| ---+---------+---------+---------+---------+---------+---------+---------+---------+---------+---------+---------+---------+---------+---------+-------- |
| PRCV-137isolate-86135308-OM830320.1-UK .......................C............................................................................................T................................... 2888 |
| PRCV-GXBS-B8-2023-PQ204803 .......................................................................................................................T................................ 2888 |
| PRCV-GXHZ-B1-2023-PQ204802 .......................................................................................................................T................................ 2888 |
| PRCV-GXHZ-K5-2023-PQ204804 .......................................................................................................................T................................ 2888 |
| PRCV-GXHZ-K17-2023-PQ204805 .......................................................................................................................T................................ 2888 |
| PRCV-GXLB-M66-2023-PQ204806 ..............................................................T........................................................T..T............................. 2888 |
| PRCV-GXLB-M67-2023-PQ204807 ..............................................................T........................................................T..T............................. 2888 |
| PRCV-GXLB-M68-2023-PQ204808 ..............................................................T........................................................T..T............................. 2888 |
| PRCV-GXNN-W2-2023-PQ204800 ..........................G............................................................................................T..T............................. 2888 |
| PRCV-GXNN-W4-2023-PQ204801 .......................................................................................................................T..T............................. 2888 |
| PRCV-GXNN-X35-2024-PQ204809 .......................................................................................................................T................................ 2888 |
| PRCV-GXNN-X83-2022-PQ204794 .........................................G..C....................T..........................................C..........T................................ 2888 |
| PRCV-GXNN-X103-2022-PQ204795 ............................................C....................T..........................................C..........T................................ 2888 |
| PRCV-GXNN-X168-2022-PQ204796 ............................................C....................T..........................................C..........T................................ 2888 |
| PRCV-GXNN-X227-2022-PQ204797 ............................................C....................T..........................................C..........T................................ 2888 |
| PRCV-GXNN-X232-2022-PQ204798 ........................................T...C....................T..........................................C..........T................................ 2888 |
| PRCV-GXNN-X238-2024-PQ204810 .......................................................................................................................T................................ 2888 |
| PRCV-GXNN-X259-2022-PQ204799 ............................................C....................T..........................................C..........T................................ 2888 |
| PRCV-90-DK-OK078898.1-Denmark .......................C..........................................C.................................................T................................... 2888 |
| PRCV-91V44-OR689864.1-Belgium ..................................................................C.................................................T................................... 2888 |
| PRCV-135isolate-86135308-OM830318.1-UK .......................C............................................................................................T.......................T........... 2888 |
| PRCV-310isolate-AR310-OM830319.1-USA ....................................................................C...................C.....................T.....G.....................C............. 2888 |
| PRCV-1894X-OR209253.1-USA ..........................G.......................G.................C...................C.....................T.....G.....................T.TC.......... 2888 |
| PRCV-1508712-III-NPTV-Parma-OR689863.1-Italy..C...............................................................C.....................C.............T.............T......A............................ 2888 |
| PRCV-86137004-X60089.1-UK .......................C............................................................................................T................................... 2888 |
| PRCV-AR310-OR209251.1-USA ....................................................................C...................C.....................T.....G.....................C............. 2888 |
| PRCV-HOL87-M94097-Netherlands ................................................................C.C.................................................T................................... 2888 |
| PRCV-ISU-1-DQ811787.1-USA ....................................................................C...................C.....................T.....G.....................C............. 2888 |
| PRCV-ISU-1-OM830321.1-USA ....................................................................C...................C.....................T.....G.....................C............. 2888 |
| PRCV-ISU20-92330-OR209254.1-USA ................................T...................................C...................C.........G...........T.....G...................T.G---G......... 2888 |
| PRCV-KPRCV2401-PP781501.1-Korea ....................................................G.............C.................................................T..T...A............................ 2888 |
| PRCV-KPRCV2402-PP781502.1-Korea ....................................................G.............C.................................................T..T...A............................ 2888 |
| PRCV-KPRCV2403-PP781503.1-Korea ..C...........................................T.....G...C.........C..........................T......................T..T...A............................ 2888 |
| PRCV-LEPP1-OR209252.1-USA ....................................................................C...................C.....................T.....G.....................C............. 2888 |
| PRCV-OH7269-KR270796.1-USA ....................................................................C...................C.........G...........T...T.G.....................---CG......... 2888 |
| PRCV-Minnesota-46140-KY406735.1-USA ....................................................................C...................C.........G...........T...T.G................G....---C.......... 2888 |
| PRCV-NM-PV096984.1-China ....................................................................C...................C.....................T.....G.....................C............. 2888 |
| PRCV-RM4-Z24675.1-France .......................C............................................................................................T................................... 2888 |
| TGEV-virulent-Purdue-DQ811789.2-USA .................A...................................C................................T.C........G..................T......C............................ 2888 |
| TGEV-Ly23-PQ189446.1-China .......................C................................................................C...........................T................................... 2888 |
| TGEV-SouthDakota154-KX900411.1-USA ..........................T..................G......................C...................C.....................T.....G......T..............C............. 2888 |
| TGEV-H16-FJ755618.2-China .......................C................................................................C...........................T................................... 2888 |
|  |
| Majority TATAGAGGACTTGCTTTTTTCTAAGGTTGTAACATCTGGTTTAGGTACAGTTGATGAAGATTACAAACGTTGTACAGGTGGTTATGACATAGCTGACTTAGTATGTGCTCAATACTATAATGGCATCATGGTGCTACCTGGTGTGGCTAATG |
| -+---------+---------+---------+---------+---------+---------+---------+---------+---------+---------+---------+---------+---------+---------+---------+ |
| 2890 2900 2910 2920 2930 2940 2950 2960 2970 2980 2990 3000 3010 3020 3030 3040 |
| -+---------+---------+---------+---------+---------+---------+---------+---------+---------+---------+---------+---------+---------+---------+---------+ |
| PRCV-137isolate-86135308-OM830320.1-UK ........................................................................................................................................................ 3040 |
| PRCV-GXBS-B8-2023-PQ204803 ........................................................................................................................................................ 3040 |
| PRCV-GXHZ-B1-2023-PQ204802 .......................................C...................................................................................T............................ 3040 |
| PRCV-GXHZ-K5-2023-PQ204804 ........................................................................................................................................................ 3040 |
| PRCV-GXHZ-K17-2023-PQ204805 ...........................................................................................................................T............................ 3040 |
| PRCV-GXLB-M66-2023-PQ204806 ........................................................................................................................................................ 3040 |
| PRCV-GXLB-M67-2023-PQ204807 ........................................................................................................................................................ 3040 |
| PRCV-GXLB-M68-2023-PQ204808 ........................................................................................................................................................ 3040 |
| PRCV-GXNN-W2-2023-PQ204800 ........................................................................................................................................................ 3040 |
| PRCV-GXNN-W4-2023-PQ204801 .........T.............................................................................................................................................. 3040 |
| PRCV-GXNN-X35-2024-PQ204809 ........................................................................................................................................................ 3040 |
| PRCV-GXNN-X83-2022-PQ204794 ........................................................................................................................................................ 3040 |
| PRCV-GXNN-X103-2022-PQ204795 ..........C............................................................................................................................................. 3040 |
| PRCV-GXNN-X168-2022-PQ204796 ........................................................................................................................................................ 3040 |
| PRCV-GXNN-X227-2022-PQ204797 ........................................................................................................................................................ 3040 |
| PRCV-GXNN-X232-2022-PQ204798 ........................................................................................................................................................ 3040 |
| PRCV-GXNN-X238-2024-PQ204810 ........................................................................................................................................................ 3040 |
| PRCV-GXNN-X259-2022-PQ204799 ...................................................................................................................................................C.... 3040 |
| PRCV-90-DK-OK078898.1-Denmark ........................................................................................................................................................ 3040 |
| PRCV-91V44-OR689864.1-Belgium ..........................................................................................................T.........................T................... 3040 |
| PRCV-135isolate-86135308-OM830318.1-UK ........................................................................................................................................................ 3040 |
| PRCV-310isolate-AR310-OM830319.1-USA ...............................................................T......................................G...........T..................................... 3040 |
| PRCV-1894X-OR209253.1-USA ...............................................................T................................T....................................................... 3040 |
| PRCV-1508712-III-NPTV-Parma-OR689863.1-Italy....................................................................................................................................T................... 3040 |
| PRCV-86137004-X60089.1-UK ........................................................................................................................................................ 3040 |
| PRCV-AR310-OR209251.1-USA ...............................................................T......................................G...........T..................................... 3040 |
| PRCV-HOL87-M94097-Netherlands ........................................................................................................................................................ 3040 |
| PRCV-ISU-1-DQ811787.1-USA ...............................................................T......................................G................................................. 3040 |
| PRCV-ISU-1-OM830321.1-USA ...............................................................T......................................G................................................. 3040 |
| PRCV-ISU20-92330-OR209254.1-USA ............A..................................................T......................................G.................C............................... 3040 |
| PRCV-KPRCV2401-PP781501.1-Korea C.................................................................................................................T.................T..............C.... 3040 |
| PRCV-KPRCV2402-PP781502.1-Korea C.................................................................................................................T.................T..............C.... 3040 |
| PRCV-KPRCV2403-PP781503.1-Korea C.................................................................................................................T.................T................... 3040 |
| PRCV-LEPP1-OR209252.1-USA ...............................................................T......................................G...........T..................................... 3040 |
| PRCV-OH7269-KR270796.1-USA ............A..................................................T......................................G.................C..T............................ 3040 |
| PRCV-Minnesota-46140-KY406735.1-USA ............A..................................................T......................................G.................C............................... 3040 |
| PRCV-NM-PV096984.1-China ...............................................................T......................................G...........T..................................... 3040 |
| PRCV-RM4-Z24675.1-France ..............................................................................................................................T......................... 3040 |
| TGEV-virulent-Purdue-DQ811789.2-USA ...................GA..........................................T........................................................................................ 3040 |
| TGEV-Ly23-PQ189446.1-China ...................G...........................................T.....................................................C.................................. 3040 |
| TGEV-SouthDakota154-KX900411.1-USA .............T...................T.............................T.......................T..............G...........T........T............................ 3040 |
| TGEV-H16-FJ755618.2-China ...................G...........................................T.....................................................C.................................. 3040 |
|  |
| Majority CTGACAAAATGACTATGTACACAGCATCCCTCGCAGGTGGTATAACATTAGGTGCACTTGGTGGAGGCGCCGTGGCTATACCTTTTGCAGTAGCAGTTCAGGCTAGACTTAATTATGTTGCTCTACAAACTGATGTATTGAACAAAAACCAG |
| ---------+---------+---------+---------+---------+---------+---------+---------+---------+---------+---------+---------+---------+---------+---------+-- |
| 3050 3060 3070 3080 3090 3100 3110 3120 3130 3140 3150 3160 3170 3180 3190 |
| ---------+---------+---------+---------+---------+---------+---------+---------+---------+---------+---------+---------+---------+---------+---------+-- |
| PRCV-137isolate-86135308-OM830320.1-UK ........................................................................................................................................................ 3192 |
| PRCV-GXBS-B8-2023-PQ204803 A.....................T.....T..T........................................................................................................................ 3192 |
| PRCV-GXHZ-B1-2023-PQ204802 A.....................T.....T..T........................................................................................................................ 3192 |
| PRCV-GXHZ-K5-2023-PQ204804 A.....................T.....T..T........................................................................................................................ 3192 |
| PRCV-GXHZ-K17-2023-PQ204805 A.....................T.....T..T........................................................................................................................ 3192 |
| PRCV-GXLB-M66-2023-PQ204806 A.....................T.....T..T........................................................................................................................ 3192 |
| PRCV-GXLB-M67-2023-PQ204807 A.....................T.....T..T........................................................................................................................ 3192 |
| PRCV-GXLB-M68-2023-PQ204808 A.....................T.....T..T........................................................................................................................ 3192 |
| PRCV-GXNN-W2-2023-PQ204800 A.....................T.....T..T.........................................T............................................A.............................T... 3192 |
| PRCV-GXNN-W4-2023-PQ204801 A.....................T.....T..T.........G...............................T............................................A.............................T... 3192 |
| PRCV-GXNN-X35-2024-PQ204809 A..................T..T.....T..T........................................................................................................................ 3192 |
| PRCV-GXNN-X83-2022-PQ204794 A.....................T.....T..T.......................G................................................................................................ 3192 |
| PRCV-GXNN-X103-2022-PQ204795 A.....................T.....T..T.......................G................................................................................................ 3192 |
| PRCV-GXNN-X168-2022-PQ204796 A.....................T.....T..T.......................G................................................................................................ 3192 |
| PRCV-GXNN-X227-2022-PQ204797 A.....................T.....T..T.......................G................................................................................................ 3192 |
| PRCV-GXNN-X232-2022-PQ204798 A.....................T.....T..T.......................G................................................................................................ 3192 |
| PRCV-GXNN-X238-2024-PQ204810 A..................T..T.....T..T........................................................................................................................ 3192 |
| PRCV-GXNN-X259-2022-PQ204799 A.....................T.....T..T.......................G................................................................................................ 3192 |
| PRCV-90-DK-OK078898.1-Denmark ...................T....................................................................................C............................................... 3192 |
| PRCV-91V44-OR689864.1-Belgium ...................T............T.............................................................T.........C............................................... 3192 |
| PRCV-135isolate-86135308-OM830318.1-UK ........................................................................................................................................................ 3192 |
| PRCV-310isolate-AR310-OM830319.1-USA .....................................................................T.....................................................................A............ 3192 |
| PRCV-1894X-OR209253.1-USA ......................................................................................T....................................................A............ 3192 |
| PRCV-1508712-III-NPTV-Parma-OR689863.1-Italy...................T..............................................................C.....................C............................................... 3192 |
| PRCV-86137004-X60089.1-UK ........................................................................................................................................................ 3192 |
| PRCV-AR310-OR209251.1-USA .....................................................................T.....................................................................A............ 3192 |
| PRCV-HOL87-M94097-Netherlands ...................T....................................T.................T.............................C............................................... 3192 |
| PRCV-ISU-1-DQ811787.1-USA ...........................................................................................................................................A............ 3192 |
| PRCV-ISU-1-OM830321.1-USA ...........................................................................................................................................A............ 3192 |
| PRCV-ISU20-92330-OR209254.1-USA ...................................................................T.....................................................................C.A............ 3192 |
| PRCV-KPRCV2401-PP781501.1-Korea ...........G.......T........T..A..................................................C.................A...C...........................................T... 3192 |
| PRCV-KPRCV2402-PP781502.1-Korea ...........G.......T........T..A..................................................C.................A...C...........................................T... 3192 |
| PRCV-KPRCV2403-PP781503.1-Korea ...................T........T..A..................................................C.................A...C...........................................T... 3192 |
| PRCV-LEPP1-OR209252.1-USA .....................................................................T.....................................................................A............ 3192 |
| PRCV-OH7269-KR270796.1-USA ...................................................................T.......................................................................A............ 3192 |
| PRCV-Minnesota-46140-KY406735.1-USA ...................................................................T.................................................................C...C.A............ 3192 |
| PRCV-NM-PV096984.1-China .....................................................................T.................................................................................. 3192 |
| PRCV-RM4-Z24675.1-France ........................................................................................................................................................ 3192 |
| TGEV-virulent-Purdue-DQ811789.2-USA ...............................T........................................................................................................................ 3192 |
| TGEV-Ly23-PQ189446.1-China ........................................................................................................................................................ 3192 |
| TGEV-SouthDakota154-KX900411.1-USA ........................................C..........................T.....................T............................A..................C.A............ 3192 |
| TGEV-H16-FJ755618.2-China ........................................................................................................................................................ 3192 |
|  |
| Majority CAGATCCTGGCTAGTGCTTTTAATCAAGCTATTGGTAACATTACACAGTCATTTGGTAAGGTTAATGATGCTATACATCAAACATCACGAGGTCTTACAACTGTTGCTAAAGCATTGGCAAAAGTGCAAGATGTTGTCAACACACAAGGTCA |
| -------+---------+---------+---------+---------+---------+---------+---------+---------+---------+---------+---------+---------+---------+---------+---- |
| 3200 3210 3220 3230 3240 3250 3260 3270 3280 3290 3300 3310 3320 3330 3340 |
| -------+---------+---------+---------+---------+---------+---------+---------+---------+---------+---------+---------+---------+---------+---------+---- |
| PRCV-137isolate-86135308-OM830320.1-UK ........................................................................................................................................................ 3344 |
| PRCV-GXBS-B8-2023-PQ204803 .....T.....C..........................T........................................................C.......................T................................ 3344 |
| PRCV-GXHZ-B1-2023-PQ204802 .....T..C..C..........................T........................................................C.......................T................................ 3344 |
| PRCV-GXHZ-K5-2023-PQ204804 .....T.....C..........................T........................................................C.......................T................................ 3344 |
| PRCV-GXHZ-K17-2023-PQ204805 .....T..C..C..........................T........................................................C.......................T................................ 3344 |
| PRCV-GXLB-M66-2023-PQ204806 .....TT....C..........................T................................................................................................................. 3344 |
| PRCV-GXLB-M67-2023-PQ204807 .....TT....C..........................T................................................................................................................. 3344 |
| PRCV-GXLB-M68-2023-PQ204808 .....TT....C..........................T................................................................................................................. 3344 |
| PRCV-GXNN-W2-2023-PQ204800 .....TT....C..........................T........................G........................................................................................ 3344 |
| PRCV-GXNN-W4-2023-PQ204801 .....TT....C..........................T................................................................................................................. 3344 |
| PRCV-GXNN-X35-2024-PQ204809 ....CT.....C..........................T........................................................C.......................T................................ 3344 |
| PRCV-GXNN-X83-2022-PQ204794 .....T.....C..........................T........................G...............................C.......................T................................ 3344 |
| PRCV-GXNN-X103-2022-PQ204795 .....T.....C..........................T........................................................C.......................T................................ 3344 |
| PRCV-GXNN-X168-2022-PQ204796 .....T.....C..........................T........................................................C.......................T................................ 3344 |
| PRCV-GXNN-X227-2022-PQ204797 .....T.....C..........................T........................................................C.......................T................................ 3344 |
| PRCV-GXNN-X232-2022-PQ204798 .....T.....C..........................T........................................................C.......................T................................ 3344 |
| PRCV-GXNN-X238-2024-PQ204810 ....CT.....C..........................T........................................................C.......................T................................ 3344 |
| PRCV-GXNN-X259-2022-PQ204799 .....T.....C..........................T........................................................C.......................T................................ 3344 |
| PRCV-90-DK-OK078898.1-Denmark ........................................................................................................................................................ 3344 |
| PRCV-91V44-OR689864.1-Belgium ......................................................................................................................G................................. 3344 |
| PRCV-135isolate-86135308-OM830318.1-UK ........................................................................................................................................................ 3344 |
| PRCV-310isolate-AR310-OM830319.1-USA ...................................................................................G............G........................................T..T........G.. 3344 |
| PRCV-1894X-OR209253.1-USA ...................................................................................G............G........................................T..T.....G..G.. 3344 |
| PRCV-1508712-III-NPTV-Parma-OR689863.1-Italy.......................................................C..............................................................G......T.......................... 3344 |
| PRCV-86137004-X60089.1-UK ........................................................................................................................................................ 3344 |
| PRCV-AR310-OR209251.1-USA ...................................................................................G............G........................................T..T........G.. 3344 |
| PRCV-HOL87-M94097-Netherlands ........................................................................................................................................................ 3344 |
| PRCV-ISU-1-DQ811787.1-USA ...................................................................................G............G........................................T..T........G.. 3344 |
| PRCV-ISU-1-OM830321.1-USA ...................................................................................G............G........................................T..T........G.. 3344 |
| PRCV-ISU20-92330-OR209254.1-USA ......T............................................................................G............G........................................T..T........G.. 3344 |
| PRCV-KPRCV2401-PP781501.1-Korea .....T................................T................C..............................................................G......T.................G........ 3344 |
| PRCV-KPRCV2402-PP781502.1-Korea .....T................................T................C..............................................................G......T.................G........ 3344 |
| PRCV-KPRCV2403-PP781503.1-Korea ......................................T................C..............................................................G......T............C............. 3344 |
| PRCV-LEPP1-OR209252.1-USA ...................................................................................G............G........................................T..T........G.. 3344 |
| PRCV-OH7269-KR270796.1-USA ......T............................................................................G............G................................A.......T..T........G.. 3344 |
| PRCV-Minnesota-46140-KY406735.1-USA ...................................................................................G............G...............................G........T..T........G.. 3344 |
| PRCV-NM-PV096984.1-China ...................................................................................G............G........................................T..T........G.. 3344 |
| PRCV-RM4-Z24675.1-France ........................................................................................................................................................ 3344 |
| TGEV-virulent-Purdue-DQ811789.2-USA .....T..............C...........................................................................G.T..................................................G.. 3344 |
| TGEV-Ly23-PQ189446.1-China ....................C........................................................................................................................G.......G.. 3344 |
| TGEV-SouthDakota154-KX900411.1-USA ...................................................................................G............G......................T.................T..T........A.. 3344 |
| TGEV-H16-FJ755618.2-China ....................C...........................................................................G............................................G.......G.. 3344 |
|  |
| Majority AGCTTTAAGACACCTAACAGTACAATTGCAAAATAATTTCCAAGCCATTAGTAGTTCTATTAGTGACATTTATAATAGGCTTGATGAATTGAGTGCTGATGCACAAGTCGACAGGCTGATCACAGGAAGACTTACAGCACTTAATGCATTTG |
| -----+---------+---------+---------+---------+---------+---------+---------+---------+---------+---------+---------+---------+---------+---------+------ |
| 3350 3360 3370 3380 3390 3400 3410 3420 3430 3440 3450 3460 3470 3480 3490 |
| -----+---------+---------+---------+---------+---------+---------+---------+---------+---------+---------+---------+---------+---------+---------+------ |
| PRCV-137isolate-86135308-OM830320.1-UK ........................................................................................................................................................ 3496 |
| PRCV-GXBS-B8-2023-PQ204803 .................................C...........T..................................................................................................C....... 3496 |
| PRCV-GXHZ-B1-2023-PQ204802 .................................C...........T..................................................................................................C....... 3496 |
| PRCV-GXHZ-K5-2023-PQ204804 ............T....................C...........T..................................................................................................C....... 3496 |
| PRCV-GXHZ-K17-2023-PQ204805 .................................C...........T..................................................................................................C....... 3496 |
| PRCV-GXLB-M66-2023-PQ204806 .................................C...........T.................................................................T........T.......................C....... 3496 |
| PRCV-GXLB-M67-2023-PQ204807 .................................C...........T.................................................................T........T.......................C....... 3496 |
| PRCV-GXLB-M68-2023-PQ204808 .................................C...........T.................................................................T........T.......................C....... 3496 |
| PRCV-GXNN-W2-2023-PQ204800 .................................C...........T.................................................................T........T.......................C....... 3496 |
| PRCV-GXNN-W4-2023-PQ204801 .................................C...........T.................................................................T........T.......................C....... 3496 |
| PRCV-GXNN-X35-2024-PQ204809 .................................C...........T..................................................................................................C....... 3496 |
| PRCV-GXNN-X83-2022-PQ204794 .................................C...........T................................................................................G.................C....... 3496 |
| PRCV-GXNN-X103-2022-PQ204795 .................................C...........T................................................................................G.................C....... 3496 |
| PRCV-GXNN-X168-2022-PQ204796 .................................C...........T................................................................................G.................C....... 3496 |
| PRCV-GXNN-X227-2022-PQ204797 .................................C...........T................................................................................G.................C....... 3496 |
| PRCV-GXNN-X232-2022-PQ204798 .................................C...........T................................................................................G.................C....... 3496 |
| PRCV-GXNN-X238-2024-PQ204810 .................................C...........T..................................................................................................C....... 3496 |
| PRCV-GXNN-X259-2022-PQ204799 .................................C...........T................................................................................G.................C....... 3496 |
| PRCV-90-DK-OK078898.1-Denmark ........................................................................................................................................................ 3496 |
| PRCV-91V44-OR689864.1-Belgium ........................................................................................................................................................ 3496 |
| PRCV-135isolate-86135308-OM830318.1-UK ........................................................................................................................................................ 3496 |
| PRCV-310isolate-AR310-OM830319.1-USA .........C............................................C.....................................................T........................................... 3496 |
| PRCV-1894X-OR209253.1-USA C........C.........T........................................................................................T........................................... 3496 |
| PRCV-1508712-III-NPTV-Parma-OR689863.1-Italy............................................................................................................T........................................... 3496 |
| PRCV-86137004-X60089.1-UK ........................................................................................................................................................ 3496 |
| PRCV-AR310-OR209251.1-USA .........C............................................C.....................................................T........................................... 3496 |
| PRCV-HOL87-M94097-Netherlands ........................................................................................................................................................ 3496 |
| PRCV-ISU-1-DQ811787.1-USA .........C............................................C.....................................................T........................................... 3496 |
| PRCV-ISU-1-OM830321.1-USA .........C............................................C.....................................................T........................................... 3496 |
| PRCV-ISU20-92330-OR209254.1-USA .........C............................................C.....................................................T........................................... 3496 |
| PRCV-KPRCV2401-PP781501.1-Korea .....................T............................................T.........................................T........T.................................. 3496 |
| PRCV-KPRCV2402-PP781502.1-Korea .....................T............................................T.........................................T........T.................................. 3496 |
| PRCV-KPRCV2403-PP781503.1-Korea ........A............T......................................................................................T........................................... 3496 |
| PRCV-LEPP1-OR209252.1-USA .........C............................................C.....................................................T........................................... 3496 |
| PRCV-OH7269-KR270796.1-USA .........C.............................T.....T........C.....................................T...............T........................................... 3496 |
| PRCV-Minnesota-46140-KY406735.1-USA .........C............................................C.....................................................T........................................... 3496 |
| PRCV-NM-PV096984.1-China .........C............................................C.....................................................T........................................... 3496 |
| PRCV-RM4-Z24675.1-France ........................................................................................................................................................ 3496 |
| TGEV-virulent-Purdue-DQ811789.2-USA .........C..........................................................................C.......................T........................................... 3496 |
| TGEV-Ly23-PQ189446.1-China .........C..................................................................................................T........................................... 3496 |
| TGEV-SouthDakota154-KX900411.1-USA .........C..........................C.................C...........T.........................................T........................................... 3496 |
| TGEV-H16-FJ755618.2-China .........C..................................................................................................T........................................... 3496 |
|  |
| Majority TTTCTCAGACTCTAACCAGACAAGCTGAGGTTAGGGCTAGTAGACAACTTGCTAAAGACAAGGTTAATGAATGCGTTAGGTCTCAGTCTCAGAGATTCGGCTTCTGTGGTAATGGTACACATTTGTTTTCACTCGCAAATGCAGCACCAAAT |
| ---+---------+---------+---------+---------+---------+---------+---------+---------+---------+---------+---------+---------+---------+---------+-------- |
| 3500 3510 3520 3530 3540 3550 3560 3570 3580 3590 3600 3610 3620 3630 3640 |
| ---+---------+---------+---------+---------+---------+---------+---------+---------+---------+---------+---------+---------+---------+---------+-------- |
| PRCV-137isolate-86135308-OM830320.1-UK .G.......................C.................................................................T............................................................ 3648 |
| PRCV-GXBS-B8-2023-PQ204803 ...........T..................................................................................G..T.................C.................................... 3648 |
| PRCV-GXHZ-B1-2023-PQ204802 ...........T..................................................................................G..T.........A.......C.................................... 3648 |
| PRCV-GXHZ-K5-2023-PQ204804 ...........T..................................................................................G..T.................C.................................... 3648 |
| PRCV-GXHZ-K17-2023-PQ204805 ...........T..................................................................................G..T.................C.................................... 3648 |
| PRCV-GXLB-M66-2023-PQ204806 .............................................................A.................A..............G..T...................................................... 3648 |
| PRCV-GXLB-M67-2023-PQ204807 ...................G.........................................A.................A........C.....G..T...................................................... 3648 |
| PRCV-GXLB-M68-2023-PQ204808 .............................................................A.................A..............G..T...................................................... 3648 |
| PRCV-GXNN-W2-2023-PQ204800 .............................................................A.................A..............G..T...................................................... 3648 |
| PRCV-GXNN-W4-2023-PQ204801 .............................................................A.................A..............G..T...................................................... 3648 |
| PRCV-GXNN-X35-2024-PQ204809 ...........T..................................................................................G..T.................C.................................... 3648 |
| PRCV-GXNN-X83-2022-PQ204794 ..............................................................................................G..T.................C.................................... 3648 |
| PRCV-GXNN-X103-2022-PQ204795 ..............................................................................................G..T.................C.................................... 3648 |
| PRCV-GXNN-X168-2022-PQ204796 ..............................................................................................G..T.................C.................................... 3648 |
| PRCV-GXNN-X227-2022-PQ204797 ..............................................................................................G..T.................C.................................... 3648 |
| PRCV-GXNN-X232-2022-PQ204798 ..............................................................................................G..T.................C.................................... 3648 |
| PRCV-GXNN-X238-2024-PQ204810 ...........T..................................................................................G..T.................C.................................... 3648 |
| PRCV-GXNN-X259-2022-PQ204799 ..............................................................................................G..T.................C.................................... 3648 |
| PRCV-90-DK-OK078898.1-Denmark .G.......................C.............................................................................................................................. 3648 |
| PRCV-91V44-OR689864.1-Belgium .G.......................C.............................................................................................................................. 3648 |
| PRCV-135isolate-86135308-OM830318.1-UK .G.......................C.............................................................................................................................. 3648 |
| PRCV-310isolate-AR310-OM830319.1-USA .A.......................C..........................C................................................................................................... 3648 |
| PRCV-1894X-OR209253.1-USA .A.......................C..........................C....................T.............................................................................. 3648 |
| PRCV-1508712-III-NPTV-Parma-OR689863.1-Italy.G.......................A.............................................................................................................................. 3648 |
| PRCV-86137004-X60089.1-UK .G.......................C....................................................A............T............................................................ 3648 |
| PRCV-AR310-OR209251.1-USA .A.......................C..........................C................................................................................................... 3648 |
| PRCV-HOL87-M94097-Netherlands .G.......................C.............................................................................................................................. 3648 |
| PRCV-ISU-1-DQ811787.1-USA .A.......................C..........................C................................................................................................... 3648 |
| PRCV-ISU-1-OM830321.1-USA .A.......................C..........................C................................................................................................... 3648 |
| PRCV-ISU20-92330-OR209254.1-USA .A..................................................C...................................C.....G........................................................C 3648 |
| PRCV-KPRCV2401-PP781501.1-Korea .G.......................C...............................................T.............................T................................................ 3648 |
| PRCV-KPRCV2402-PP781502.1-Korea .G.......................C...............................................T.............................T................................................ 3648 |
| PRCV-KPRCV2403-PP781503.1-Korea .G.......................C...............................................T.............................T.......................C........................ 3648 |
| PRCV-LEPP1-OR209252.1-USA .A.......................C..........................C................................................................................................... 3648 |
| PRCV-OH7269-KR270796.1-USA .A..................................................C..................................................T................................................ 3648 |
| PRCV-Minnesota-46140-KY406735.1-USA .A..........................A............C..........C...................................C............................................................... 3648 |
| PRCV-NM-PV096984.1-China .A.......................C..........................C................................................................................................... 3648 |
| PRCV-RM4-Z24675.1-France .G.......................C.............................................................................................................................. 3648 |
| TGEV-virulent-Purdue-DQ811789.2-USA .G.......................G..........................C...............................................A................................................... 3648 |
| TGEV-Ly23-PQ189446.1-China .G.......................C..........................C...............................................A................................................... 3648 |
| TGEV-SouthDakota154-KX900411.1-USA .A..................................................C...................................C........T...................................................... 3648 |
| TGEV-H16-FJ755618.2-China .G.......................C..........................C...............................................A................................................... 3648 |
|  |
| Majority GGCATGATTTTCTTTCACACAGTGTTATTACCAACGGCTTATGAAACTGTGACTGCTTGGTCAGGTATTTGTGCTTTAGATGGTGATCGCACTTTTGGACTTGTCGTTAAAGATGTCCAGTTGACTTTATTTCGTAATCTAGATGACAAGTT |
| -+---------+---------+---------+---------+---------+---------+---------+---------+---------+---------+---------+---------+---------+---------+---------+ |
| 3650 3660 3670 3680 3690 3700 3710 3720 3730 3740 3750 3760 3770 3780 3790 3800 |
| -+---------+---------+---------+---------+---------+---------+---------+---------+---------+---------+---------+---------+---------+---------+---------+ |
| PRCV-137isolate-86135308-OM830320.1-UK ........C...............C............................................................................................................................... 3800 |
| PRCV-GXBS-B8-2023-PQ204803 .................................................................C..................A...............................T................................... 3800 |
| PRCV-GXHZ-B1-2023-PQ204802 .................................................................C..................A...............................T................................... 3800 |
| PRCV-GXHZ-K5-2023-PQ204804 .................................................................C..................A...............................T................................... 3800 |
| PRCV-GXHZ-K17-2023-PQ204805 .................................................................C..................A...............................T................................... 3800 |
| PRCV-GXLB-M66-2023-PQ204806 ........C........................................................C..................A...............................T................................... 3800 |
| PRCV-GXLB-M67-2023-PQ204807 ........C........................................................C..................A................................................................... 3800 |
| PRCV-GXLB-M68-2023-PQ204808 ........C...................................G....................C.C................A...............................T................................... 3800 |
| PRCV-GXNN-W2-2023-PQ204800 ........C........................................................C..................A...............................T................................... 3800 |
| PRCV-GXNN-W4-2023-PQ204801 ........C........................................................C..................A...............................T................................... 3800 |
| PRCV-GXNN-X35-2024-PQ204809 .................................................................C..................A...............................T................................... 3800 |
| PRCV-GXNN-X83-2022-PQ204794 .................................................................C..................A...............................T................................... 3800 |
| PRCV-GXNN-X103-2022-PQ204795 .................................................................C..................A...............................T................................... 3800 |
| PRCV-GXNN-X168-2022-PQ204796 .................................................................C..................A...............................T................................... 3800 |
| PRCV-GXNN-X227-2022-PQ204797 .................................................................C..................A...............................T................................... 3800 |
| PRCV-GXNN-X232-2022-PQ204798 .................................................................C..................A...............................T................................... 3800 |
| PRCV-GXNN-X238-2024-PQ204810 .................................................................C..................A.......C........................................................... 3800 |
| PRCV-GXNN-X259-2022-PQ204799 .................................................................C..................A...............................T................................... 3800 |
| PRCV-90-DK-OK078898.1-Denmark ........C...............C............................................................................................................................... 3800 |
| PRCV-91V44-OR689864.1-Belgium ........C...............C............................................................................C.................................................. 3800 |
| PRCV-135isolate-86135308-OM830318.1-UK ........C...............C............................................................................................................................... 3800 |
| PRCV-310isolate-AR310-OM830319.1-USA ........................C.............C.....................G...........................................................C.......G....................... 3800 |
| PRCV-1894X-OR209253.1-USA ........C...............C.............C.....................G.....................................C.....................C.......G....................... 3800 |
| PRCV-1508712-III-NPTV-Parma-OR689863.1-Italy........C...............C............................................................................C..T............................................... 3800 |
| PRCV-86137004-X60089.1-UK ........C...............C............................................................................................................................T.. 3800 |
| PRCV-AR310-OR209251.1-USA ........................C.............C.....................G...........................................................C.......G....................... 3800 |
| PRCV-HOL87-M94097-Netherlands ........C...............C............................................................................................................................... 3800 |
| PRCV-ISU-1-DQ811787.1-USA ........................C.............C.....................G.........................C.................................C.......G....................... 3800 |
| PRCV-ISU-1-OM830321.1-USA ........................C.............C.....................G.........................C.................................C.......G....................... 3800 |
| PRCV-ISU20-92330-OR209254.1-USA ......C...............................C.........................................................................................G....................... 3800 |
| PRCV-KPRCV2401-PP781501.1-Korea ........................C................................................T...........................C..T.........................................T..... 3800 |
| PRCV-KPRCV2402-PP781502.1-Korea ........................C................................................T...........................C..T.........................................T..... 3800 |
| PRCV-KPRCV2403-PP781503.1-Korea ........................C................................................T...................C.......C..T.........................................T..... 3800 |
| PRCV-LEPP1-OR209252.1-USA ........................C.............C.....................G...........................................................C.......G....................... 3800 |
| PRCV-OH7269-KR270796.1-USA ........C.............................C..C......................................................................................G....................... 3800 |
| PRCV-Minnesota-46140-KY406735.1-USA ......................................C.....................G...................................................................G....................... 3800 |
| PRCV-NM-PV096984.1-China ........................C.............C.....................G...........................................................C............................... 3800 |
| PRCV-RM4-Z24675.1-France ........C...............C.............G...........................................T..................................................................... 3800 |
| TGEV-virulent-Purdue-DQ811789.2-USA ........................C...................................G...............C...................................................G....................... 3800 |
| TGEV-Ly23-PQ189446.1-China ........C............T..C...................................G...................................................................G....................... 3800 |
| TGEV-SouthDakota154-KX900411.1-USA ........C.............................C.....................G.......................................................T...........G....................... 3800 |
| TGEV-H16-FJ755618.2-China ........C............T..C...................................G...................................................................G....................... 3800 |
|  |
| Majority CTATTTGACACCCAGAACTATGTATCAGCCTAGAGTGGCAACTAGTTCTGATTTTGTTCAAATTGAAGGGTGCGATGTGCTGTTTGTTAATACAACTGTAAGTGATTTGCCTAGTATTATACCTGATTATATTGATATTAATCAGACTGTTC |
| ---------+---------+---------+---------+---------+---------+---------+---------+---------+---------+---------+---------+---------+---------+---------+-- |
| 3810 3820 3830 3840 3850 3860 3870 3880 3890 3900 3910 3920 3930 3940 3950 |
| ---------+---------+---------+---------+---------+---------+---------+---------+---------+---------+---------+---------+---------+---------+---------+-- |
| PRCV-137isolate-86135308-OM830320.1-UK ........................................................................................................................................................ 3952 |
| PRCV-GXBS-B8-2023-PQ204803 .......................................................A....................................................A........................................... 3952 |
| PRCV-GXHZ-B1-2023-PQ204802 .......................................................A....................................................A........................................... 3952 |
| PRCV-GXHZ-K5-2023-PQ204804 .......................................................A....................................................A........................................... 3952 |
| PRCV-GXHZ-K17-2023-PQ204805 .......................................................A....................................................A........................................... 3952 |
| PRCV-GXLB-M66-2023-PQ204806 .......................................................A................................................................................................ 3952 |
| PRCV-GXLB-M67-2023-PQ204807 ........................C..........C...................A................................................................................................ 3952 |
| PRCV-GXLB-M68-2023-PQ204808 .......................................................A................................................................................................ 3952 |
| PRCV-GXNN-W2-2023-PQ204800 .......................................................A......................T......................................................................... 3952 |
| PRCV-GXNN-W4-2023-PQ204801 .......................................................A......................T......................................................................... 3952 |
| PRCV-GXNN-X35-2024-PQ204809 .......................................................A....................................................A........................................... 3952 |
| PRCV-GXNN-X83-2022-PQ204794 .......................................................A................................................................................................ 3952 |
| PRCV-GXNN-X103-2022-PQ204795 .......................................................A................................................................................................ 3952 |
| PRCV-GXNN-X168-2022-PQ204796 .......................................................A................................................................................................ 3952 |
| PRCV-GXNN-X227-2022-PQ204797 .......................................................A................................................................................................ 3952 |
| PRCV-GXNN-X232-2022-PQ204798 .......................................................A................................................................................................ 3952 |
| PRCV-GXNN-X238-2024-PQ204810 .....C.............................C...................A....................................................A........................................... 3952 |
| PRCV-GXNN-X259-2022-PQ204799 .....................................A.................A................................................................................................ 3952 |
| PRCV-90-DK-OK078898.1-Denmark ...........................................................................................................................C............................ 3952 |
| PRCV-91V44-OR689864.1-Belgium ....................................T...................................................................C............................................... 3952 |
| PRCV-135isolate-86135308-OM830318.1-UK ........................................................................................................................................................ 3952 |
| PRCV-310isolate-AR310-OM830319.1-USA .........C.................................................................................G....................................................A....... 3952 |
| PRCV-1894X-OR209253.1-USA .........C..............................................................................C..G.....T..............................................A....... 3952 |
| PRCV-1508712-III-NPTV-Parma-OR689863.1-Italy....................................T..................................................................TCG.............................................. 3952 |
| PRCV-86137004-X60089.1-UK ........................................................................................................................................................ 3952 |
| PRCV-AR310-OR209251.1-USA .........C.................................................................................G....................................................A....... 3952 |
| PRCV-HOL87-M94097-Netherlands ........................................................................................................................................................ 3952 |
| PRCV-ISU-1-DQ811787.1-USA .........C.................................................................................G....................................................A....... 3952 |
| PRCV-ISU-1-OM830321.1-USA .........C.................................................................................G....................................................A....... 3952 |
| PRCV-ISU20-92330-OR209254.1-USA T........C....................................................................T............G...............................C....................A....... 3952 |
| PRCV-KPRCV2401-PP781501.1-Korea ....................................T................................T...............................T.TCG.............................................. 3952 |
| PRCV-KPRCV2402-PP781502.1-Korea ....................................T................................T...............................T.TCG.............................................. 3952 |
| PRCV-KPRCV2403-PP781503.1-Korea ....C..............................CT................................T.................................TCG.............................................. 3952 |
| PRCV-LEPP1-OR209252.1-USA .........C.................................................................................G....................................................A....... 3952 |
| PRCV-OH7269-KR270796.1-USA T.....A..C....................................................................T............G...............................C....................A....... 3952 |
| PRCV-Minnesota-46140-KY406735.1-USA T........C....................................................................T............G...............................C....................A....... 3952 |
| PRCV-NM-PV096984.1-China ...........................................................................................G....................................................A....... 3952 |
| PRCV-RM4-Z24675.1-France ........................................................................................................................................................ 3952 |
| TGEV-virulent-Purdue-DQ811789.2-USA .........C..........................T..............C.......................................G............................................................ 3952 |
| TGEV-Ly23-PQ189446.1-China .........C.................................................................................G............................................................ 3952 |
| TGEV-SouthDakota154-KX900411.1-USA T........C.................................................................................G...............................C....................A....... 3952 |
| TGEV-H16-FJ755618.2-China .........C.................................................................................G............................................................ 3952 |
|  |
| Majority AAGACATATTAGAAAATTTTAGACCAAATTGGACTGTACCTGAGCTGACATTGGACGTTTTTAACGCAACCTATTTAAACCTGACTGGTGAAATTGATGACTTAGAGTTTAGGTCAGAAAAGCTACATAACACTACTGTAGAACTTGCCATT |
| -------+---------+---------+---------+---------+---------+---------+---------+---------+---------+---------+---------+---------+---------+---------+---- |
| 3960 3970 3980 3990 4000 4010 4020 4030 4040 4050 4060 4070 4080 4090 4100 |
| -------+---------+---------+---------+---------+---------+---------+---------+---------+---------+---------+---------+---------+---------+---------+---- |
| PRCV-137isolate-86135308-OM830320.1-UK ........................................................................................................................................................ 4104 |
| PRCV-GXBS-B8-2023-PQ204803 ........................T..............................T.........A..............T.........................................T...........G................. 4104 |
| PRCV-GXHZ-B1-2023-PQ204802 ........................T..............................T.........A..............T.........................................T............................. 4104 |
| PRCV-GXHZ-K5-2023-PQ204804 ........................T..............................T.........A..............T.........................................T............................. 4104 |
| PRCV-GXHZ-K17-2023-PQ204805 ........................T..............................T.........A..............T.........................................T............................. 4104 |
| PRCV-GXLB-M66-2023-PQ204806 ........................T..............................T.........A..............T.........................................T............................. 4104 |
| PRCV-GXLB-M67-2023-PQ204807 ........................T..............................T.........A..............T.........................................T............................. 4104 |
| PRCV-GXLB-M68-2023-PQ204808 ........................T..............................T.........A..............T.........................................T............................. 4104 |
| PRCV-GXNN-W2-2023-PQ204800 ........................T..............................T.........A..............T.........................................T............................. 4104 |
| PRCV-GXNN-W4-2023-PQ204801 ........................T..............................T.........A..............T.........................................T............................. 4104 |
| PRCV-GXNN-X35-2024-PQ204809 ........................T..............................T.........A..............T.........................................T............................. 4104 |
| PRCV-GXNN-X83-2022-PQ204794 ........................T..............................T.........A..............T...................A....................AT............................. 4104 |
| PRCV-GXNN-X103-2022-PQ204795 ........................T..............................T.........A..............T...................A....................AT............................. 4104 |
| PRCV-GXNN-X168-2022-PQ204796 ........................T..............................T.........A..............T...................A....................AT............................. 4104 |
| PRCV-GXNN-X227-2022-PQ204797 ........................T..............................T.........A..............T...................A....................AT............................. 4104 |
| PRCV-GXNN-X232-2022-PQ204798 ........................T..............................T.........A..............T...................A....................AT............................. 4104 |
| PRCV-GXNN-X238-2024-PQ204810 ........................T..............................T.........A..............T.........................................T............................. 4104 |
| PRCV-GXNN-X259-2022-PQ204799 ........................T..............................T.........A..............T...................A....................AT............................. 4104 |
| PRCV-90-DK-OK078898.1-Denmark ........................................................................................................................................................ 4104 |
| PRCV-91V44-OR689864.1-Belgium ...........................................T............................................................................................................ 4104 |
| PRCV-135isolate-86135308-OM830318.1-UK ........................................................................................................................................................ 4104 |
| PRCV-310isolate-AR310-OM830319.1-USA ........C..........C.......................TT.......T...A......................T.......T..................A............................................. 4104 |
| PRCV-1894X-OR209253.1-USA ................C..........................TT.......T...A..............................T..................A............................................. 4104 |
| PRCV-1508712-III-NPTV-Parma-OR689863.1-Italy........................T..................T..T......................................................................................C.................. 4104 |
| PRCV-86137004-X60089.1-UK .................................................TA..................................................................................................... 4104 |
| PRCV-AR310-OR209251.1-USA ........C..........C.......................TT.......T...A......................T.......T..................A............................................. 4104 |
| PRCV-HOL87-M94097-Netherlands ........................................................................................................................................................ 4104 |
| PRCV-ISU-1-DQ811787.1-USA ........C..........C.......................TT.......T...A..............................T..................A............................................. 4104 |
| PRCV-ISU-1-OM830321.1-USA ........C..........C.......................TT.......T...A..............................T..................A............................................. 4104 |
| PRCV-ISU20-92330-OR209254.1-USA ........C..................................TT.......T...A.......T..............T.......T..................A...............T................G............ 4104 |
| PRCV-KPRCV2401-PP781501.1-Korea ...........................................T...........T..............T.........T....................................................................... 4104 |
| PRCV-KPRCV2402-PP781502.1-Korea ...........................................T...........T..............T.........T....................................................................... 4104 |
| PRCV-KPRCV2403-PP781503.1-Korea ...........................................T...........T..............T...................................................T............................. 4104 |
| PRCV-LEPP1-OR209252.1-USA ........C..........C.......................TT.......T...A......................T.......T..................A............................................. 4104 |
| PRCV-OH7269-KR270796.1-USA ........C..........C........C..............TT.......T...A.......T..............T.......T..................A...............T................G............ 4104 |
| PRCV-Minnesota-46140-KY406735.1-USA ........C..........C...T....C.......C......TT.......T...A.......T..............T.......T..................A............................................. 4104 |
| PRCV-NM-PV096984.1-China ........C..........C.......................TT.......T...A......................T.......T..................A............................................. 4104 |
| PRCV-RM4-Z24675.1-France ........................................................................................................................................................ 4104 |
| TGEV-virulent-Purdue-DQ811789.2-USA ............................................T.......T...A.................................................A..........................C.................. 4104 |
| TGEV-Ly23-PQ189446.1-China ............................................T.......T...A.................................................A............................................. 4104 |
| TGEV-SouthDakota154-KX900411.1-USA ...................C.......................TT.......T...A.......T..............T.......T..................A.......................T..................... 4104 |
| TGEV-H16-FJ755618.2-China ............................................T.......T...A.................................................A............................................. 4104 |
|  |
| Majority CTTATTGACAACATTAACAATACATTAGTCAATCTTGAATGGCTTAATAGAATTGAAACTTATGTAAAATGGCCTTGGTATGTGTGGCTACTAATAGGCTTAGTAGTAATATTTTGCATACCATTAATGCTATTTTGCTGTTGTAGTACAGG |
| -----+---------+---------+---------+---------+---------+---------+---------+---------+---------+---------+---------+---------+---------+---------+------ |
| 4110 4120 4130 4140 4150 4160 4170 4180 4190 4200 4210 4220 4230 4240 4250 |
| -----+---------+---------+---------+---------+---------+---------+---------+---------+---------+---------+---------+---------+---------+---------+------ |
| PRCV-137isolate-86135308-OM830320.1-UK ..C...........................................................................................................................C......................... 4256 |
| PRCV-GXBS-B8-2023-PQ204803 ...........T.....T......................................G.......................C........G......................................T....................... 4256 |
| PRCV-GXHZ-B1-2023-PQ204802 ...........T.....T......................................G.......................C........G......................................T....................... 4256 |
| PRCV-GXHZ-K5-2023-PQ204804 ...........T.....T......................................G................................G......................................T....................... 4256 |
| PRCV-GXHZ-K17-2023-PQ204805 ...........T.....T......................................G.......................C........G......................................T....................... 4256 |
| PRCV-GXLB-M66-2023-PQ204806 ..C..............T......................................G................................G.........................T.................................... 4256 |
| PRCV-GXLB-M67-2023-PQ204807 ..C..............T......................................G................................G.........................T.................................... 4256 |
| PRCV-GXLB-M68-2023-PQ204808 ..C..............T......................................G................................G.........................T.................................... 4256 |
| PRCV-GXNN-W2-2023-PQ204800 ..C..............T......................................G.....................A..........G.........................T.................................... 4256 |
| PRCV-GXNN-W4-2023-PQ204801 ..C..............T......................................G................................G.........................T.................................... 4256 |
| PRCV-GXNN-X35-2024-PQ204809 ..C..............T......................................G.......................C........G......................................T..G.................... 4256 |
| PRCV-GXNN-X83-2022-PQ204794 .................T......................................G................................G.............................................................. 4256 |
| PRCV-GXNN-X103-2022-PQ204795 .................T......................................G................................G.............................................................. 4256 |
| PRCV-GXNN-X168-2022-PQ204796 .................T......................................G................................G.............................................................. 4256 |
| PRCV-GXNN-X227-2022-PQ204797 .................T......................................G................................G.............................................................. 4256 |
| PRCV-GXNN-X232-2022-PQ204798 .................T......................................G................................G.............................................................. 4256 |
| PRCV-GXNN-X238-2024-PQ204810 .................T......................................G.......................C........G......................................T..G.................... 4256 |
| PRCV-GXNN-X259-2022-PQ204799 .................T......................................G................................G.............................................................. 4256 |
| PRCV-90-DK-OK078898.1-Denmark ..C...........................................................................................................................C...............T......... 4256 |
| PRCV-91V44-OR689864.1-Belgium ..C...........................................................................................................................T............T..T......... 4256 |
| PRCV-135isolate-86135308-OM830318.1-UK ..C...........................................................................................................................C...............T......... 4256 |
| PRCV-310isolate-AR310-OM830319.1-USA ...........................................................C......................................T...........................T......................... 4256 |
| PRCV-1894X-OR209253.1-USA ..................................................................................................T...........................T......................... 4256 |
| PRCV-1508712-III-NPTV-Parma-OR689863.1-Italy..C...........................................................................................................................C...............T......... 4256 |
| PRCV-86137004-X60089.1-UK ..C...........................................................................................................................C......................... 4256 |
| PRCV-AR310-OR209251.1-USA ...........................................................C......................................T...........................T......................... 4256 |
| PRCV-HOL87-M94097-Netherlands ..C........................................................C..................................................................C......................... 4256 |
| PRCV-ISU-1-DQ811787.1-USA ...........................................................C......................................T...........................T......................... 4256 |
| PRCV-ISU-1-OM830321.1-USA ...........................................................C......................................T...........................T......................... 4256 |
| PRCV-ISU20-92330-OR209254.1-USA .......CT..............T...................................C.....G..............................A.T...........G...............T...................C..... 4256 |
| PRCV-KPRCV2401-PP781501.1-Korea .................T................................G........C......................................T.......................T...T......................... 4256 |
| PRCV-KPRCV2402-PP781502.1-Korea .................T................................G........C......................................T.......................T...T......................... 4256 |
| PRCV-KPRCV2403-PP781503.1-Korea .................T................................G........C......................................T........................C..T......................... 4256 |
| PRCV-LEPP1-OR209252.1-USA ...........................................................C......................................T...........................T......................... 4256 |
| PRCV-OH7269-KR270796.1-USA .......GT...............C..................................C.....T................................T...........................T...................C..... 4256 |
| PRCV-Minnesota-46140-KY406735.1-USA .......GT...............C....T.............................C....................................TTT...........................T...................C..... 4256 |
| PRCV-NM-PV096984.1-China ...........................................................C......................................T...........................T......................... 4256 |
| PRCV-RM4-Z24675.1-France ..C.....................G.....................................................................................................C......................... 4256 |
| TGEV-virulent-Purdue-DQ811789.2-USA ..C.........................................C..............C..................................................................C......................... 4256 |
| TGEV-Ly23-PQ189446.1-China ............................................C..............C..................................................................C......................... 4256 |
| TGEV-SouthDakota154-KX900411.1-USA .......GT...............C.......G.................................................................T.................T.........T...................C..... 4256 |
| TGEV-H16-FJ755618.2-China ............................................C..............C..................................................................C......................... 4256 |
|  |
| Majority TTGCTGTGGATGCATAGGTTGTTTAGGAAGTTGTTGTCACTCTATATTCAGTAGAAGACAATTTGAAAATTATGAACCTATTGAAAAAGTGCACGTCCATTAA |
| ---+---------+---------+---------+---------+---------+---------+---------+---------+---------+--------- |
| 4260 4270 4280 4290 4300 4310 4320 4330 4340 4350 |
| ---+---------+---------+---------+---------+---------+---------+---------+---------+---------+--------- |
| PRCV-137isolate-86135308-OM830320.1-UK ....................................................................................................... 4359 |
| PRCV-GXBS-B8-2023-PQ204803 ..........CT.........................................................C.T.....T......................... 4359 |
| PRCV-GXHZ-B1-2023-PQ204802 ..........CT.........................................................C.T.....T......................... 4359 |
| PRCV-GXHZ-K5-2023-PQ204804 ..........CT.........................................................C.T.....T......................... 4359 |
| PRCV-GXHZ-K17-2023-PQ204805 ..........CT.........................................................C.T.....T......................... 4359 |
| PRCV-GXLB-M66-2023-PQ204806 ...........T.........................................................C.T.....T......................... 4359 |
| PRCV-GXLB-M67-2023-PQ204807 ...........T.........................................................C.T.....T......................... 4359 |
| PRCV-GXLB-M68-2023-PQ204808 ...........T.........................................................C.T.....T......................... 4359 |
| PRCV-GXNN-W2-2023-PQ204800 ...........T.........................................................C.T.....T......................... 4359 |
| PRCV-GXNN-W4-2023-PQ204801 ...........T.........................................................C.T.....T......................... 4359 |
| PRCV-GXNN-X35-2024-PQ204809 ..........CT.........................................................C.T.....T......................... 4359 |
| PRCV-GXNN-X83-2022-PQ204794 ...........T.........................................................C.T.....T......................... 4359 |
| PRCV-GXNN-X103-2022-PQ204795 ...........T.........................................................C.T.....T......................... 4359 |
| PRCV-GXNN-X168-2022-PQ204796 ...........T.........................................................C.T.....T......................... 4359 |
| PRCV-GXNN-X227-2022-PQ204797 ...........T.........................................................C.T.....T......................... 4359 |
| PRCV-GXNN-X232-2022-PQ204798 ...........T.........................................................C.T.....T......................... 4359 |
| PRCV-GXNN-X238-2024-PQ204810 ..........CT...............................G.........................C.T.....T..C...................... 4359 |
| PRCV-GXNN-X259-2022-PQ204799 ...........T.........................................................C.T.....T......................... 4359 |
| PRCV-90-DK-OK078898.1-Denmark ............T..........................T......................................................A........ 4359 |
| PRCV-91V44-OR689864.1-Belgium ............T.................................................................................A........ 4359 |
| PRCV-135isolate-86135308-OM830318.1-UK ....................................................................................................... 4359 |
| PRCV-310isolate-AR310-OM830319.1-USA ...............................................G....................................................... 4359 |
| PRCV-1894X-OR209253.1-USA ...............................................G....................................................... 4359 |
| PRCV-1508712-III-NPTV-Parma-OR689863.1-Italy............TG................................................................................A........ 4359 |
| PRCV-86137004-X60089.1-UK ....................................................................................................... 4359 |
| PRCV-AR310-OR209251.1-USA ...............................................G....................................................... 4359 |
| PRCV-HOL87-M94097-Netherlands ............T...............................................G.......................................... 4359 |
| PRCV-ISU-1-DQ811787.1-USA ...............................................G....................................................... 4359 |
| PRCV-ISU-1-OM830321.1-USA ...............................................G....................................................... 4359 |
| PRCV-ISU20-92330-OR209254.1-USA .......................................T.......G...................................C................... 4359 |
| PRCV-KPRCV2401-PP781501.1-Korea .............................A.........T..................................T............................ 4359 |
| PRCV-KPRCV2402-PP781502.1-Korea .............................A.........T..................................T............................ 4359 |
| PRCV-KPRCV2403-PP781503.1-Korea .............................A.........T..................................T............................ 4359 |
| PRCV-LEPP1-OR209252.1-USA ...............................................G....................................................... 4359 |
| PRCV-OH7269-KR270796.1-USA .........G................T............T.......G....................................................... 4359 |
| PRCV-Minnesota-46140-KY406735.1-USA ...........T...........................T.......G....................................................... 4359 |
| PRCV-NM-PV096984.1-China ...............................................G....................................................... 4359 |
| PRCV-RM4-Z24675.1-France ....................................................................................................... 4359 |
| TGEV-virulent-Purdue-DQ811789.2-USA ...............................................GT.......................C.....A........................ 4359 |
| TGEV-Ly23-PQ189446.1-China .......................................T.......G........................C.............................. 4359 |
| TGEV-SouthDakota154-KX900411.1-USA .......................................T.......G....................................................... 4359 |
| TGEV-H16-FJ755618.2-China ...............................................G........................C.............................. 4359 |

**Figure S1** Nucleotide sequence analysis of PRCV S gene.
