# Supplementary figures and images for: Phylogenetic analysis and genetic evolution of porcine respiratory coronavirus in Guangxi province, Southern China from 2022 to 2024
Source: Front Microbiol. 2025 Jul 10;16:1625343. doi: 10.3389/fmicb.2025.1625343 (PMC12287051; doi:10.3389/fmicb.2025.1625343)

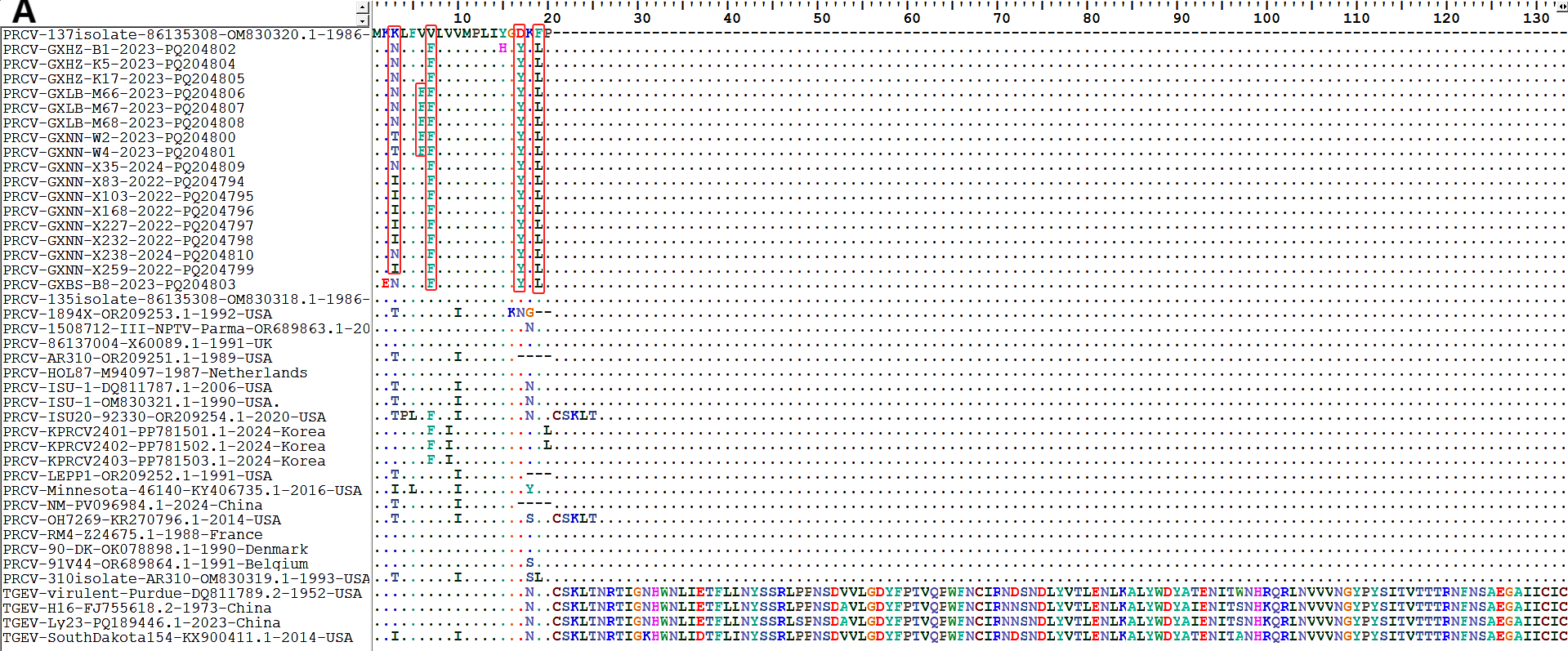


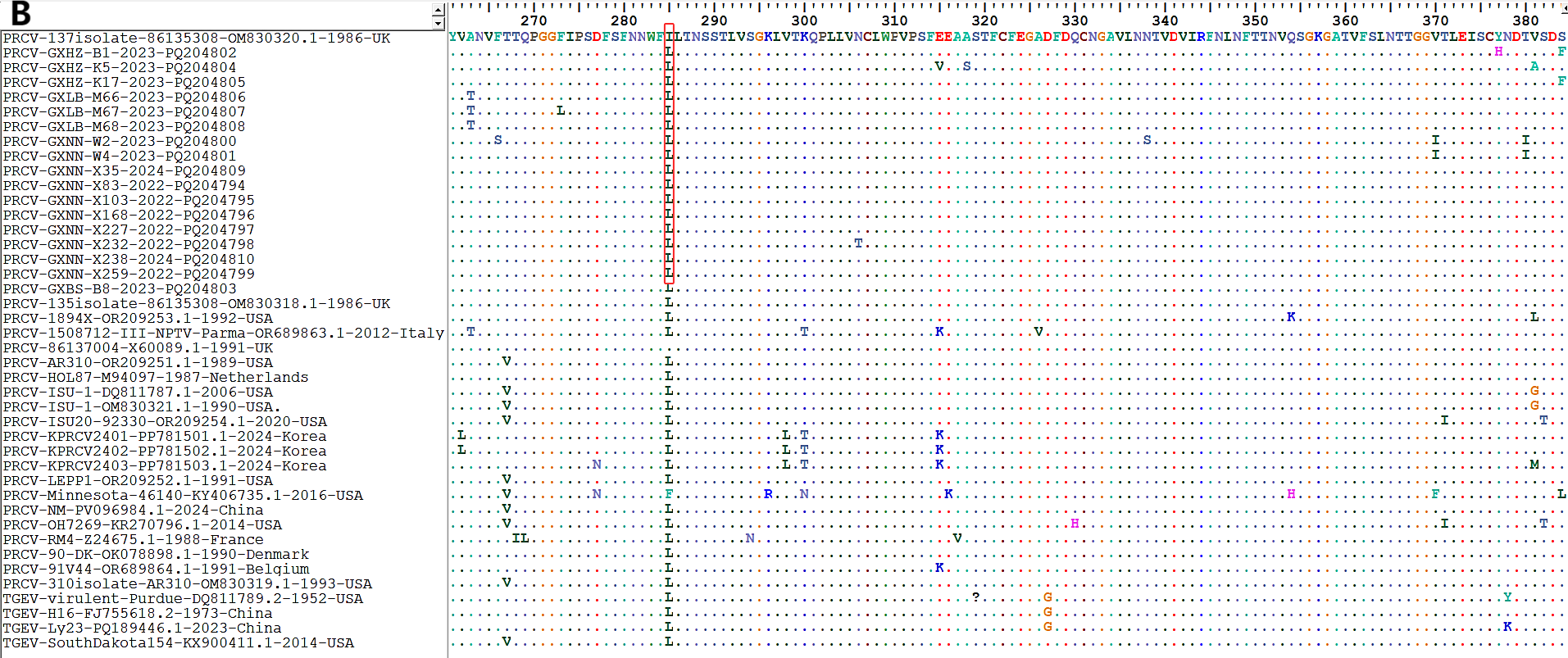


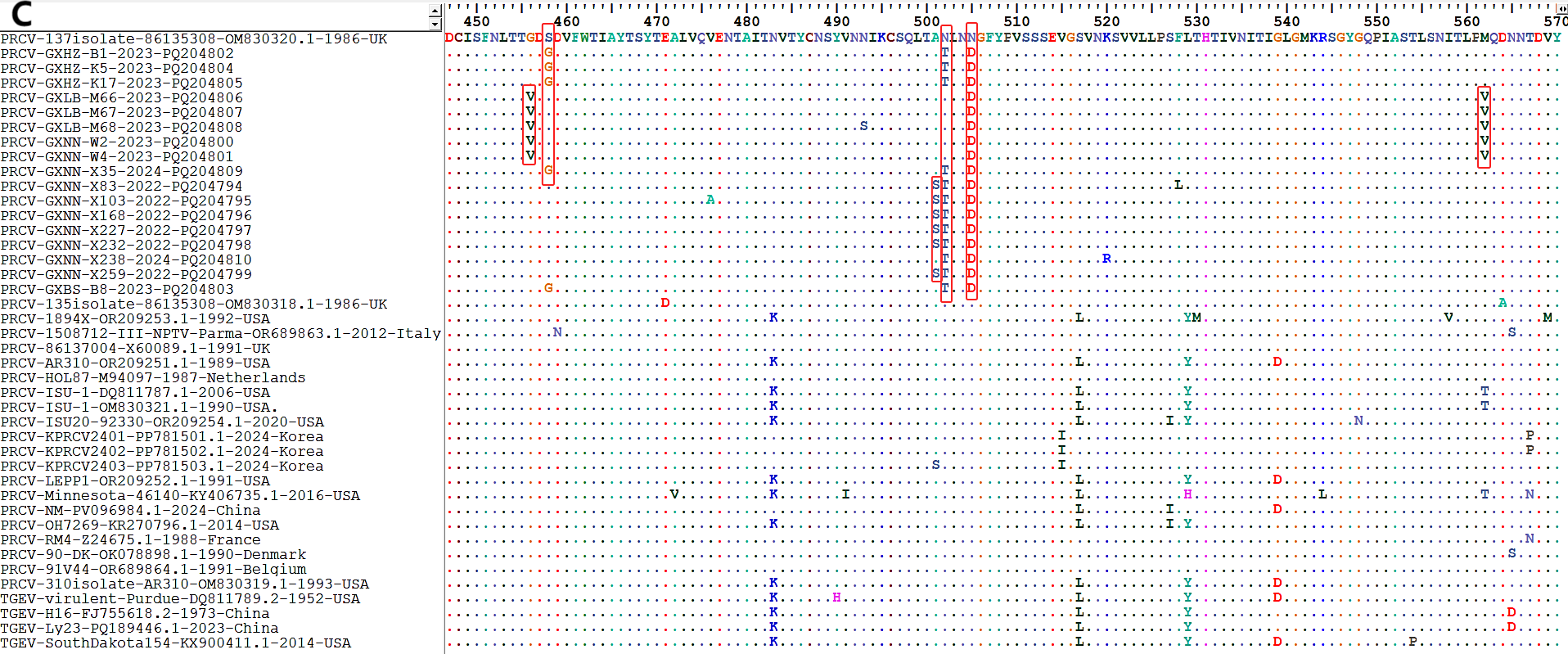


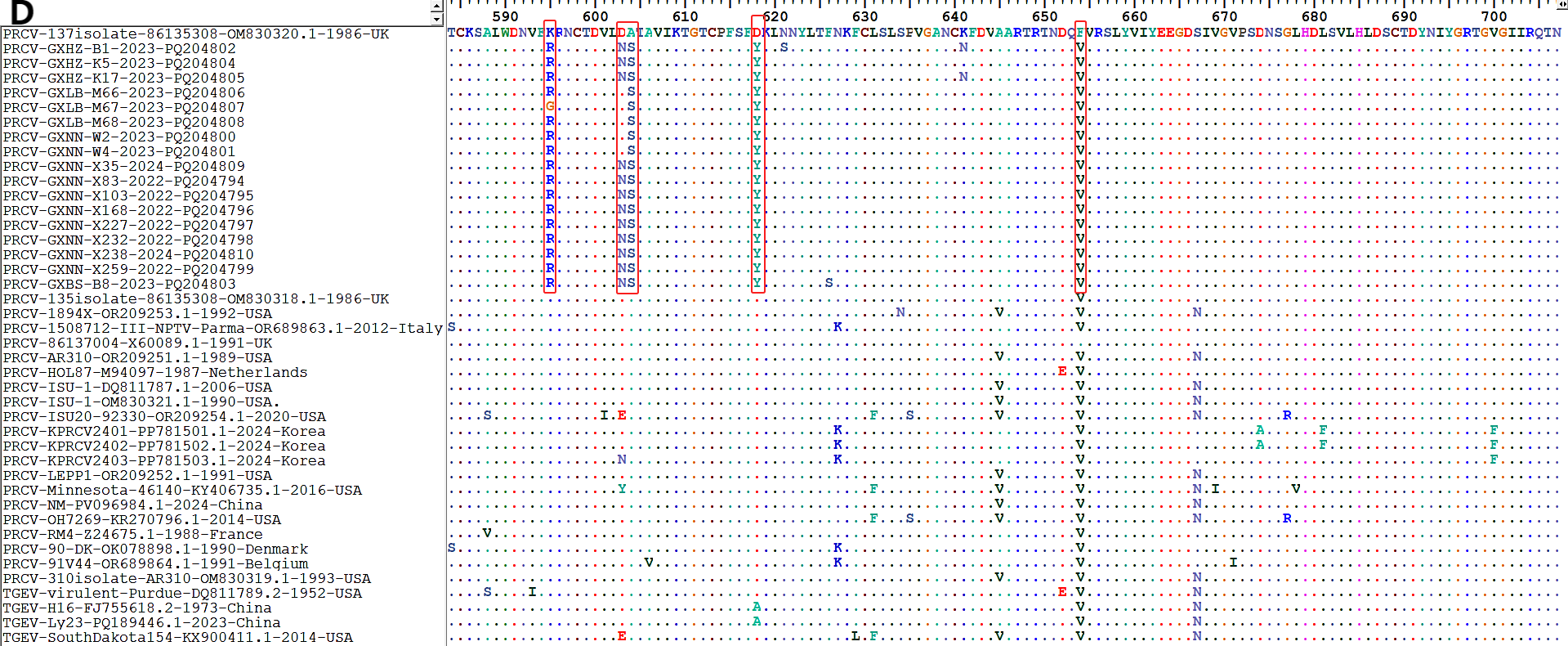


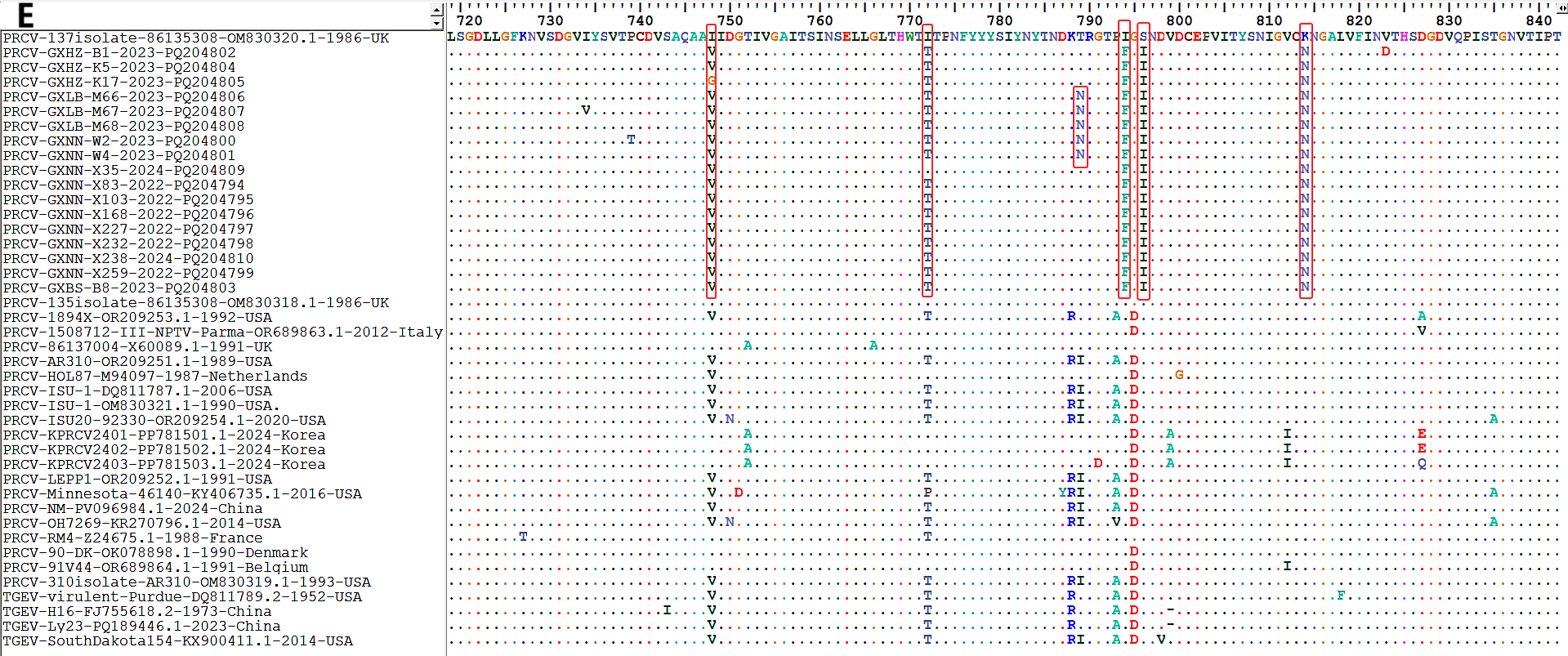


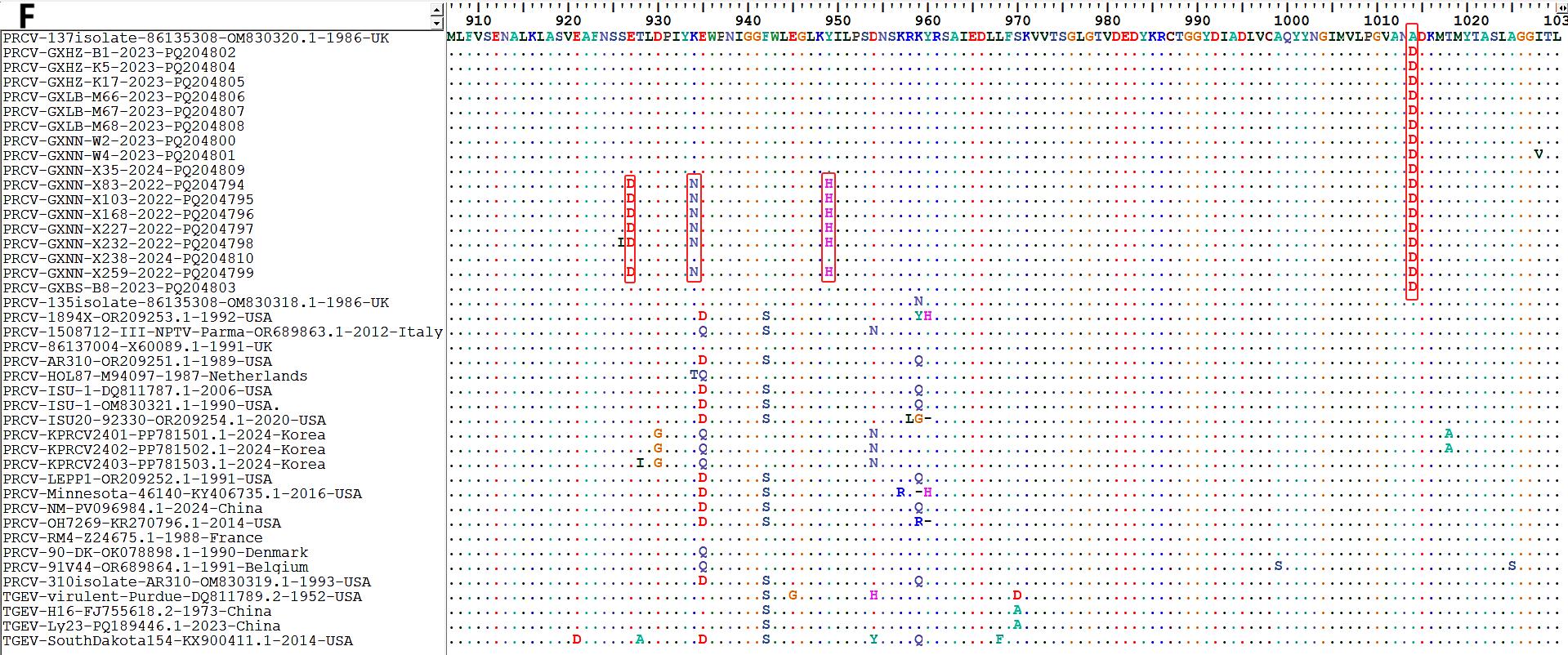


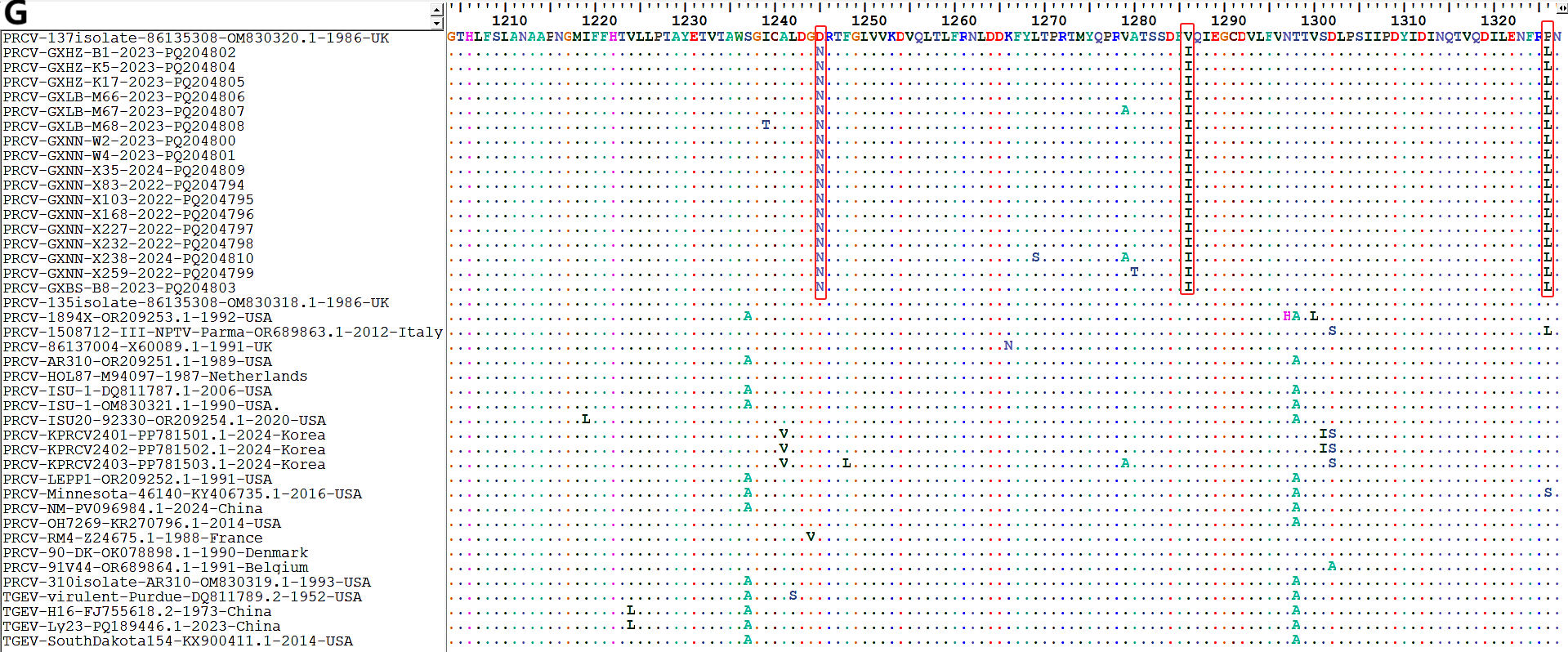


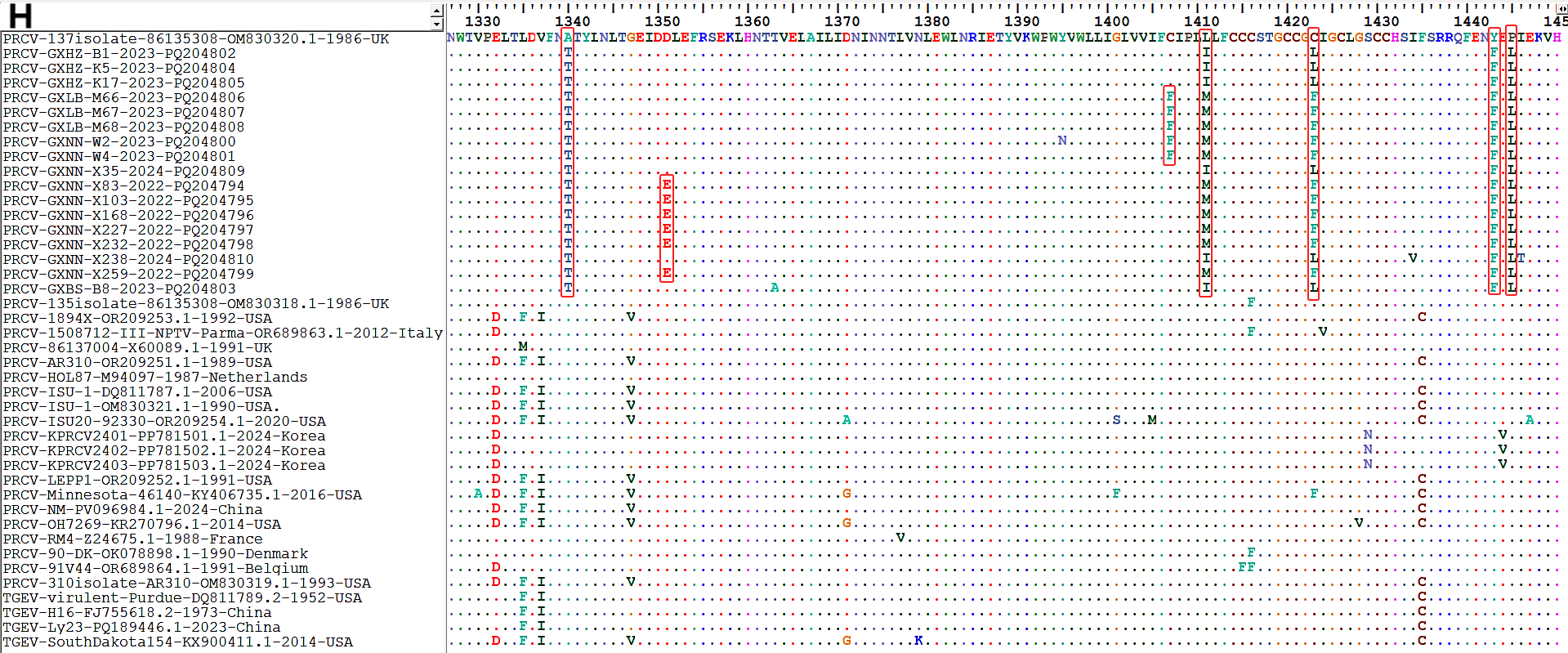


**FIGURE S2**

Amino acid sequence analysis of PRCV S gene. (A–H) show the mutation sites of amino acids.

Supplement: Supplementary file 2 [file Data_Sheet_2.docx]
