# Supplementary material for: Phylogenetic analysis and genetic evolution of porcine respiratory coronavirus in Guangxi province, Southern China from 2022 to 2024
Source: Front Microbiol. 2025 Jul 10;16:1625343. doi: 10.3389/fmicb.2025.1625343 (PMC12287051; doi:10.3389/fmicb.2025.1625343)
Supplement: Supplementary file 3 [file Table_1.docx]

**Supplementary Materials**

**TABLE S1** The PRCV reference strains used in this study.

| **Strain** | **Accession Number** | **Source** | **Year** | **Gene** |
| --- | --- | --- | --- | --- |
| 135 isolate 86/135308 | OM830318.1 | United Kingdom | 1986 | S/M/N |
| 137 isolate 86/135308 | OM830320.1 | United Kingdom | 1986 | S/M/N |
| PRCV/USA/AR310/1989_ISU | OR209251.1 | USA | 1989 | S/M/N |
| ISU-1 | OM830321.1 | USA | 1990 | S/M/N |
| PRCV/USA/LEPP1/1991_ISU | OR209252.1 | USA | 1991 | S/M/N |
| PRCV/USA/1894X/1992_ISU | OR209253.1 | USA | 1992 | S/M/N |
| AR310 | OM830319.1 | USA | 1993 | S/M/N |
| PRCV ISU-1 | DQ811787.1 | USA | 2006 | S/M/N |
| OH7269 | KR270796.1 | USA | 2014 | S/M/N |
| PRCV/USA/Minnesota-46140/2016 | KY406735.1 | USA | 2016 | S/M/N |
| PRCV/USA/ISU20-92330/2020 | OR209254.1 | USA | 2020 | S/M/N |
| RM4 | Z24675.1 | France | 1988 | S/M/N |
| PRCV-1/90-DK | OK078898.1 | Denmark | 1990 | S/M/N |
| 91V44 | OR689864.1 | Belgium | 1991 | S/M/N |
| 1508712 III NPTV Parma | OR689863.1 | Italy | 2012 | S/M/N |
| PRCV/NM | PV096984.1 | China | 2024 | S/M/N |
| HOL87 | M94097.1 | Netherlands | 1987 | S |
| 86/137004 | X60089.1 | United Kingdom | 1991 | S |
| HOL87 | M94097.1 | Netherlands | 1987 | S |
| KPRCV2401 | PP781501.1 | Korea | 2024 | S |
| KPRCV2402 | PP781502.1 | Korea | 2024 | S |
| KPRCV2403 | PP781503.1 | Korea | 2024 | S |
| 86/137004 | X60056.1 | United Kingdom | 1991 | M/N |
| PRCV/DK /23/ 1 | PQ274257.1 | Denmark | 2023 | N |
| PRCV/DK /23/ 2 | PQ274258.1 | Denmark | 2023 | N |
| PRCV/DK /23/ 3 | PQ274262.1 | Denmark | 2023 | N |
| PRCV/DK /23/ 4 | PQ274259.1 | Denmark | 2023 | N |
| PRCV/DK /23/ 5 | PQ274264.1 | Denmark | 2023 | N |
| PRCV/DK /23/ 6 | PQ274266.1 | Denmark | 2023 | N |
| PRCV/DK /23/ 7 | PQ274268.1 | Denmark | 2023 | N |
| PRCV/DK /23/ 8 | PQ274270.1 | Denmark | 2023 | N |
| PRCV/DK /23/ 9 | PQ274272.1 | Denmark | 2023 | N |
| PRCV/DK /23/ 10 | PQ274274.1 | Denmark | 2023 | N |
| PRCV/DK /23/11 | PQ274260.1 | Denmark | 2023 | N |
| PRCV/DK /23/ 12 | PQ274261.1 | Denmark | 2023 | N |
| PRCV/DK /23/ 15 | PQ274277.1 | Denmark | 2023 | N |
| PRCV/DK /23/ 16 | PQ274279.1 | Denmark | 2023 | N |
| PRCV/DK /23/ 17 | PQ274281.1 | Denmark | 2023 | N |
| PRCV/DK /23/ 18 | PQ274283.1 | Denmark | 2023 | N |
| PRCV/DK /23/19 | PQ274285.1 | Denmark | 2023 | N |
| PRCV/DK /23/ 20 | PQ274287.1 | Denmark | 2023 | N |
| PRCV/DK /23/ 22 | PQ274288.1 | Denmark | 2023 | N |

**TABLE S2** The information on the 17 PRCV strains obtained in this study.

| Strain | Accession Number | | | Date | Origin | Sample Type | Source |
| --- | --- | --- | --- | --- | --- | --- | --- |
|  | S | M | N |  |  |  |  |
| GXNN-X83-2022 | PQ204794 | PQ204811 | PQ204828 | Dec, 2022 | Nanning, China | Nasal swab | Abattoir |
| GXNN-X103-2022 | PQ204795 | PQ204812 | PQ204829 | Dec, 2022 | Nanning, China | Nasal swab | Abattoir |
| GXNN-X168-2022 | PQ204796 | PQ204813 | PQ204830 | Dec, 2022 | Nanning, China | Nasal swab | Abattoir |
| GXNN-X227-2022 | PQ204797 | PQ204814 | PQ204831 | Dec, 2022 | Nanning, China | Nasal swab | Abattoir |
| GXNN-X232-2022 | PQ204798 | PQ204815 | PQ204832 | Dec, 2022 | Nanning, China | Nasal swab | Abattoir |
| GXNN-X259-2022 | PQ204799 | PQ204816 | PQ204833 | Dec, 2022 | Nanning, China | Nasal swab | Abattoir |
| GXBS-B8-2023 | PQ204803 | PQ204819 | PQ204836 | Jan, 2023 | Baise, China | tissue | Abattoir |
| GXLB-M66-2023 | PQ204806 | PQ204823 | PQ204840 | May, 2023 | Laibin, China | Nasal swab | Pig farm |
| GXLB-M67-2023 | PQ204807 | PQ204824 | PQ204841 | May, 2023 | Laibin, China | Nasal swab | Pig farm |
| GXLB-M68-2023 | PQ204808 | PQ204825 | PQ204842 | May, 2023 | Laibin, China | Nasal swab | Pig farm |
| GXHZ-K5-2023 | PQ204804 | PQ204821 | PQ204838 | Jun, 2023 | Hezhou, China | Nasal swab | Pig farm |
| GXHZ-K17-2023 | PQ204805 | PQ204822 | PQ204839 | Jun, 2023 | Hezhou, China | Nasal swab | Pig farm |
| GXHZ-B1-2023 | PQ204802 | PQ204820 | PQ204837 | Jun, 2023 | Hezhou, China | Nasal swab | Harmless treatment plant |
| GXNN-W2-2023 | PQ204800 | PQ204817 | PQ204834 | Sep, 2023 | Nanning, China | Nasal swab | Harmless treatment plant |
| GXNN-W4-2023 | PQ204801 | PQ204818 | PQ204835 | Sep, 2023 | Nanning, China | Nasal swab | Harmless treatment plant |
| GXNN-X35-2024 | PQ204809 | PQ204826 | PQ204843 | Jan, 2024 | Nanning, China | Nasal swab | Abattoir |
| GXNN-X238-2024 | PQ204810 | PQ204827 | PQ204844 | Jan, 2024 | Naning, China | Nasal swab | Abattoir |

**TABLE S3** The TGEV reference strains used in this study.

| Strain | Accession Number | Group | Source | Year | Gene |
| --- | --- | --- | --- | --- | --- |
| TGEV virulent Purdue | DQ811789.2 | Ia | USA | 1952 | S/M/N |
| SC-Y | DQ443743.1 | Ia | China | 2006 | S/M/N |
| H16 | FJ755618.2 | Ib | China | 1973 | S/M/N |
| SC2021 | ON858825.1 | Ib | China | 2021 | S/M/N |
| Ly23 | PQ189446.1 | Ib | China | 2023 | S/M/N |
| TGEV/USA/Minnesota138/2006 | KX900395.1 | II | USA | 2006 | S/M/N |
| TGEV/USA/Iowa143/2008 | KX900400.1 | II | USA | 2008 | S/M/N |
| TGEV/USA/Oklahoma147/2012 | KX900404.1 | II | USA | 2012 | S/M/N |
| TGEV/USA/Minnesota150/2013 | KX900407.1 | II | USA | 2013 | S/M/N |
| TGEV/USA/Wisconsin151/2014 | KX900408.1 | II | USA | 2014 | S/M/N |
| TGEV/USA/SouthDakota154/2014 | KX900411.1 | II | USA | 2014 | S/M/N |
| SX413 | PQ603017.1 | Ia | China | 2023 | S |
| HB-1 | MZ368889.1 | Ia | China | 2020 | M |
| TGEV/USA/HB/1988 | KX900394.1 | Ia | USA | 1988 | N |

**TABLE S4** The nucleotide mutation information on S gene of the 17 obtained PRCV strain.

| **Point** | **Mutation** | **Point** | **Mutation** | **Point** | **Mutation** |
| --- | --- | --- | --- | --- | --- |
| 8 | A→T/C | 1585 | C→T | 3072 | C→T |
| 9 | A→T | 1605 | C→T | 3095 | A→G |
| 16 | G→T | 1641 | T→C | 3198 | C→T |
| 18 | G→C | 1659 | A→T | 3199 | C→T |
| 19 | G→T | 1684 | A→G | 3204 | T→C |
| 21 | C→T | 1722 | T→C | 3231 | C→T |
| 49 | G→T | 1784 | A→G | 3288 | T→C |
| 51 | C→T | 1786 | C→A | 3312 | A→T |
| 55 | T→C | 1807 | G→A | 3378 | T→C |
| 738 | C→T | 1810 | G→T | 3390 | C→T |
| 748 | C→T | 1830 | T→C | 3456 | C→T |
| 807 | G→A | 1836 | T→C | 3465 | C→T |
| 813 | A→G | 1852 | G→T | 3471 | A→G |
| 851 | C→T | 1905 | C→T | 3489 | T→C |
| 852 | A→C | 1935 | T→C | 3598 | G→T |
| 855 | C→T | 1960 | T→G | 3508 | C→T |
| 856 | C→T | 2016 | G→T | 3522 | C→T |
| 867 | C→T | 2019 | T→C | 3558 | G→A |
| 873 | G→T | 2022 | C→T | 3576 | G→A |
| 885 | C→T | 2064 | G→A | 3588 | T→G |
| 894 | T→C | 2142 | A→G | 3591 | A→G |
| 909 | A→T | 2242 | A→G | 3594 | C→T |
| 948 | A→G | 2247 | T→C | 3612 | T→C |
| 960 | G→A | 2315 | T→C | 3657 | C→T |
| 981 | C→T | 2337 | C→T | 3673 | C→T |
| 1014 | C→T | 2366 | C→A | 3714 | T→C |
| 1020 | A→C | 2380 | A→T | 3733 | G→A |
| 1023 | C→T | 2382 | T→C | 3765 | C→T |
| 1026 | C→T | 2387 | G→T | 3856 | G→A |
| 1038 | C→T | 2403 | T→C | 3909 | G→A |
| 1092 | G→A | 2427 | C→T | 3977 | C→T |
| 1095 | C→T | 2442 | A→T | 4008 | C→T |
| 1123 | C→T | 2478 | T→G | 4018 | G→A |
| 1137 | T→C | 2511 | C→T | 4033 | C→T |
| 1152 | G→T | 2529 | C→T | 4053 | C→A |
| 1155 | C→T | 2538 | A→C | 4074 | G→A |
| 1167 | C→T | 2628 | C→T | 4075 | C→T |
| 1185 | C→T | 2646 | C→T | 4107 | C→T |
| 1203 | G→T | 2673 | A→G | 4116 | C→T |
| 1218 | C→T | 2691 | C→T | 4122 | C→T |
| 1367 | G→T | 2709 | A→G | 4161 | A→G |
| 1372 | A→G | 2760 | C→T | 4186 | T→C |
| 1404 | G→T | 2781 | A→C | 4194 | A→G |
| 1407 | C→T | 2802 | A→T | 4220 | G→T |
| 1416 | A→G | 2845 | T→C | 4231 | C→A |
| 1488 | C→T | 2853 | C→T | 4233 | G→T |
| 1501 | G→T | 2856 | C→T | 4267 | T→C |
| 1505 | A→C | 2859 | C→T | 4268 | G→T |
| 1513 | A→G | 3041 | C→A | 4326 | T→C |
| 1557 | T→C | 3063 | A→T | 4328 | A→T |
| 1573 | C→T | 3069 | C→T | 4334 | C→T |
